# CATI: an efficient gene integration method for rodent and primate embryos by MMEJ suppression

**DOI:** 10.1186/s13059-023-02987-w

**Published:** 2023-06-23

**Authors:** Hongyu Chen, Xingchen Liu, Lanxin Li, Qingtong Tan, Shiyan Li, Li Li, Chunyang Li, Jiqiang Fu, Yong Lu, Yan Wang, Yidi Sun, Zhen-Ge Luo, Zongyang Lu, Qiang Sun, Zhen Liu

**Affiliations:** 1 Institute of Neuroscience, State Key Laboratory of Neuroscience, Center for Excellence in Brain Science & Intelligence Technology, Chinese Academy of Sciences, 320 Yue-Yang Road, Shanghai, 200031 China; 2 University of Chinese Academy of Sciences, 19A Yu-Quan Road, Beijing, 100049 China; 3 School of Life Science and Technology, ShanghaiTech University, Shanghai, 201210 China; 4 Shanghai Center for Brain Science and Brain-Inspired Intelligence Technology, Shanghai, 201210 China

## Abstract

The efficiency of homology-directed repair (HDR) plays a crucial role in the development of animal models and gene therapy. We demonstrate that microhomology-mediated end-joining (MMEJ) constitutes a substantial proportion of DNA repair during CRISPR-mediated gene editing. Using CasRx to downregulate a key MMEJ factor, Polymerase Q (*Polq*), we improve the targeted integration efficiency of linearized DNA fragments and single-strand oligonucleotides (ssODN) in mouse embryos and offspring. CasRX-assisted targeted integration (CATI) also leads to substantial improvements in HDR efficiency during the CRISPR/Cas9 editing of monkey embryos. We present a promising tool for generating monkey models and developing gene therapies for clinical trials.

**Supplementary Information:**

The online version contains supplementary material available at 10.1186/s13059-023-02987-w.

## Background

Genome editing using the CRISPR/Cas9 system is dependent upon the rectification of DNA double-strand breaks (DSBs), which are instigated by the sgRNA-guided Cas9 endonuclease [1, 2]. The process of homology-directed repair (HDR) facilitates the accurate integration of donor DNA, allowing for the introduction of specific genomic alterations such as sequence substitution, insertion, and deletion [1, 2]. Consequently, HDR has become a widely employed method for the creation of gene-modified animal models and the correction of germ-cell genes through zygotic injection involving CRISPR/Cas9 reagents and exogenous DNA donors possessing homologous arms [3–5]. Despite the advances in genome editing, the efficiency of precise DNA fragment integration through HDR remains suboptimal. Recently, several methods have been reported to enhance gene knock-in efficiency in zygotes via Cas9 (mRNA or ribonucleoprotein) injection. These approaches include the utilization of transgene templates cleavable into 800 bp homology arms in vivo (HMEJ strategy) [6], linearized templates derived from PCR amplification (Tild-CRISPR) [7], long single-stranded DNA (ssDNA) templates (Easi-CRISPR) [8], short ssODN templates [9], templates that activate the MMEJ or NHEJ pathways [10–12], and templates modified with biotin-avidin system (CAB system) [13, 14]. Furthermore, chemically modified single-guide RNAs (sgRNA) [15] and commercially available, chemically modified tracrRNAs and crRNAs [16, 17] have also been employed to augment gene knock-in efficiency. However, only a limited number of successfully targeted loci have been documented thus far [8, 12, 18]. Certain methods, such as Easi-CRISPR and ssODN, are constrained by the size limitations of DNA fragments for insertion (typically < 1 kb). Other approaches, including those based on MMEJ or NHEJ templates, are restricted by the generation of imprecise junctions at the editing sites. Additionally, methods involving modified templates or sgRNAs may be hindered by cytotoxicity. Furthermore, techniques employing long ssDNA-based Easi-CRISPR are constrained by challenges in obtaining templates and elevated costs.

The overall efficiency of precise DNA integration through HDR has been relatively low, due to competition from other repair pathways such as NHEJ [19], MMEJ [20], and single-strand annealing (SSA) [21]. Consequently, obstructing these competing repair pathways has emerged as a prevalent strategy to enhance HDR efficiency. Inhibition of the NHEJ repair pathway has been previously employed to improve HDR efficacy [22, 23]; however, this approach proved ineffective in several investigations [24, 25]. Recent studies have indicated that MMEJ is a significant DSB repair pathway and may cooperate with other repair pathways or function independently in specific types of DNA damage [26]. Moreover, both MMEJ and HDR necessitate DNA end resection and may directly compete with one another [27, 28]. As a result, we explored the potential of augmenting HDR efficiency by modulating MMEJ repair-associated proteins.

In this study, we initially investigated the DNA repair pathways and the expression of genes associated with DNA repair during CRISPR/Cas9 editing in mouse embryos. We discovered that MMEJ constitutes a substantial proportion of DNA repair during CRISPR-mediated embryonic gene editing and, in some loci, even surpasses the efficiency of NHEJ repair. Additionally, we observed an upregulation in the expression of DNA polymerase *Polq*, known to facilitate MMEJ during DSB repair [29], during CRISPR/Cas9 editing in mouse embryos. Subsequently, we found that silencing *Polq* expression through RNA editor CasRX led to a significant increase in HDR-mediated DNA integration efficiency in mouse embryos. Notably, this CasRX-assisted targeted integration (CATI) method also enhanced HDR efficiency in monkey embryos. Therefore, the CATI approach could be a preferred option for developing gene-edited monkey models and human germ-cell gene therapies.

## Results

### The MMEJ repair pathway holds a significant proportion in the repair of CRISPR-mediated embryonic gene damage

**Fig. 1:**
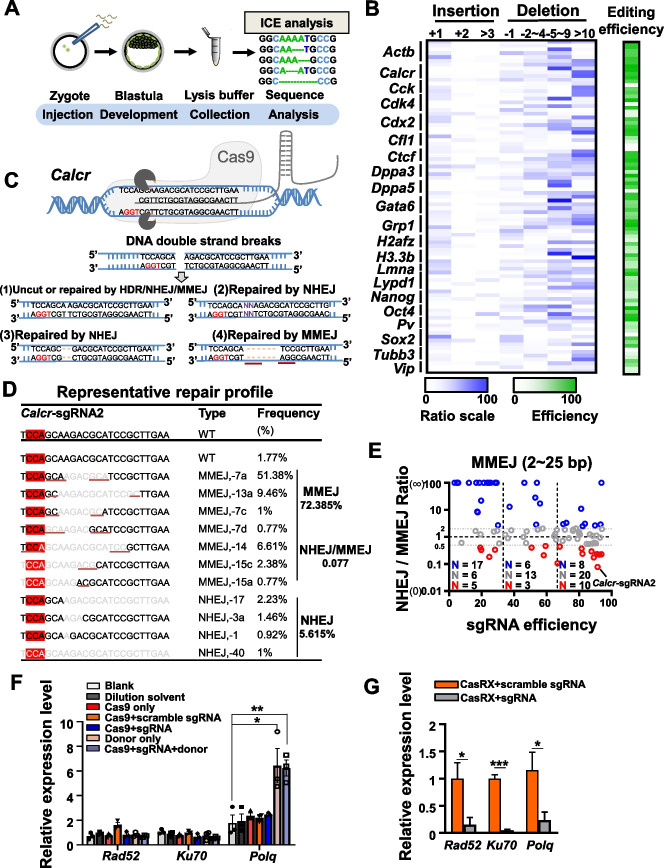
Analysis of DSB repair patterns in CRISPR/Cas9-mediated embryo editing. **A** Schematic representation of the analysis of DSB repair patterns in CRISPR/Cas9-mediated embryo gene editing. **B** A summary of the editing efficiency for the tested sgRNAs. The blue scale represents the editing efficiency of insertions and deletions, while the green scale illustrates the overall editing efficiency. **C** CRISPR-Cas9 cleavage and its subsequent repair processes are depicted, where the PAM sequence is denoted by red bases, the insertion sequence by purple bases, the deletion sequence by a yellow dotted line, and the micro-homologous arm by a red underline. **D** The repair profile of *Calcr*-sgRNA2 is presented, wherein the PAM sequence (NGG) is highlighted in red, the missing sequence is represented by a gray base region, and the micro-homologous sequence is underlined in red. **E** The relationship between sgRNA editing efficiency and the NHEJ/MMEJ ratio. Each circle corresponds to a specific sgRNA, with *Calcr*-sg2 highlighted. Data are presented as mean ± standard error of the mean (s.e.m.). **F** The relative expression levels of the indicated genes (*Rad52*, *Ku70*, and *Polq*). The “Blank” group refers to embryos without any treatment, the “Dilution solvent” group corresponds to embryos injected with dilution buffer, the “Cas9 only” group represents embryos injected with Cas9 mRNA only, and the “Donor only” group signifies embryos injected with donor DNA only. A two-sided Student’s *t*-test was used for statistical analysis, with *p < 0.05 and **p < 0.01 considered significant. Data are presented as mean ± standard error of the mean (s.e.m.). Each bar includes at least two biological replicates. **G** Gene knockdown via CasRX. RT-PCR analysis revealed the expression levels of *Rad52*, *Ku70*, and *Polq* in both control and CasRX-treated embryos. The orange bar denotes the Cas9/scramble gRNA group, which serves as a control. A two-sided Student’s *t*-test was used for statistical analysis, with *p < 0.05 and ***p < 0.001 considered significant. Data are presented as mean ± standard error of the mean (s.e.m.). Each bar includes at least three biological replicates

Numerous prior studies have demonstrated that embryonic HDR efficiency can be enhanced through the modification of DNA repair pathways [22, 23]. Nonetheless, the exact repair pathway responsible for endogenous DNA damage following CRISPR/Cas9 editing in embryos has yet to be thoroughly characterized. Consequently, we conducted a large-scale indel analysis of embryo editing outcomes, guided by 88 individual sgRNAs. These sgRNAs were designed to target 21 genes, encompassing housekeeping, pluripotency, and neuron-specific genes. We microinjected individual sgRNA and Cas9 mRNA into mouse zygotes and collected the blastocysts for genotyping analysis, utilizing the ICE v2 CRISPR analysis tool (ice.synthego.com) (Fig. 1A and Additional file 1: Fig. S1A). In total, approximately 1500 mouse embryos were injected with 88 individual sgRNA/Cas9 mRNA combinations (> 10 embryos per sgRNA). The overall editing frequencies for various sgRNAs ranged from 1.68 to 94%, with nucleotide deletion more prevalent than insertion (Fig. 1B and Additional file 1: Fig. S1B).

Subsequently, we investigated the repair pattern of targeted genes in mouse embryos following genetic modification utilizing the CRISPR/Cas9 system. In accordance with prior research [24, 26, 30, 31], we classified the DNA repair pattern as NHEJ when insertion occurred or in cases where microhomology sequences were not detected in the vicinity of the deletion segment, or when the deletion involved only a single base. Conversely, deletion events accompanied by microhomology sequences (2 ~ 25 nt) were classified as MMEJ repair pattern (Fig. 1C). In our analysis of each individual sgRNA-guided embryonic modification, we generally observed the presence of both NHEJ and MMEJ repair pattern (Fig. 1D and Additional file 2: Table S1). Subsequently, we determined the frequency ratio of NHEJ to MMEJ in all embryos subjected to each sgRNA-guided modification, where we discovered that 18 out of 88 (20.45%) sgRNA-guided modifications exhibited a MMEJ-biased repair pattern, characterized by a NHEJ/MMEJ frequency ratio of less than 0.5. For instance, *Calcr*-sgRNA2 editing exhibited a pronounced MMEJ-biased repair, with NHEJ and MMEJ frequencies of 5.615% and 72.385% (ratio 0.077), respectively (Fig. 1D). Among other 70 sgRNAs, 44.29% (31/70) showed a 0.5 ~ 2 NHEJ/MMEJ ratio, indicating that MMEJ plays a significant role in the repair of CRISPR-mediated DSB damage in embryos (Fig. 1E). Furthermore, we observed that higher sgRNA editing efficiency corresponds to a larger proportion of MMEJ repair patterns. When sgRNA editing efficiency is less than 66.7%, 16% (8/50) sgRNA-guided modification shows a preference for the MMEJ repair pattern. Conversely, when sgRNA editing efficiency exceeds 66.7%, 26% (10/38) sgRNA-guided modification displays a preference for the MMEJ repair pattern (Fig. 1E). These findings suggest that MMEJ may have a critical function in the repair of nuclease-mediated DSBs in embryos, especially in instances of high-efficiency sgRNA-guided editing.

### *Polq* exhibits upregulation during the CRISPR/Cas9-mediated embryonic editing process

DSB damage repair is crucial for maintaining normal cellular functions [32], and several genes associated with the DSB response have been identified [19, 33, 34]. To examine the expression levels of DSB repair-related genes during embryonic gene editing, we selected the *Actb* gene as the target site and injected various editing regents into embryos. We conducted RNA-seq analysis on embryos subjected to targeted knock-out and donor DNA integration at the *Actb* gene locus. By comparing the RNA-seq data of embryos injected with Cas9 + sgRNA (for *Actb* knock-out) and those with Cas9 + sgRNA + donor DNA (for mCherry knock-in at *Actb*, the reagents merely suffice for the mCherry knock-in process), we aimed to identify genes affected during DSB repair. Our analysis revealed that only a few genes exhibited changes in the expression levels during *Actb* knock-out; however, the expression of over 200 genes was altered in the presence of donor DNA in the knock-in group (Additional file 1: Fig. S1C and S1D). We speculated that these alterations might result from the presence of abundant linearized donor DNA, which could be recognized by embryos as DNA damage.

Subsequently, we compared the expression levels of HDR, NHEJ, SSA, and MMEJ repair pathway-related genes in the RNA-seq data from the three groups of embryos (Additional file 1: Fig. S1E). Our analysis reveals that the majority of these genes did not display altered expression levels. However, *Polq*, a key MMEJ factor, exhibited an approximately 2-fold upregulation in the knock-in embryo editing group (Additional file 1: Fig. S1E). We conducted RT-PCR analysis to assess the expression levels of *Rad52*, *Ku70*, and *Polq*, which were considered key factors in SSA, NHEJ, and MMEJ repair pathways, respectively. This analysis corroborated the trend of increased *polq* expression in the knock-in embryo editing group (Fig. 1F); however, in the control group, aside from the elevated *Polq* levels in the “donor only” group, there were no significant changes. This confirmed our hypothesis that linearized DNA was recognized as damage by the DNA damage response system, resulting in the induction of increased *Polq* levels (Fig. 1F). These findings prompted us to explore the possibility of enhancing HDR efficiency by inhibiting MMEJ repair via downregulation of *Polq* expression.

### *Polq* knockdown via CasRX enhances HDR efficiency in mouse embryos

Initially, we attempted to knock down key DSB repair-related factors (*Rad52*, *Ku70*, and *Polq*) using an siRNA strategy (three random siRNAs for each gene) in mouse embryos. However, we observed that none of these three genes was downregulated in either single gene (*polq*) siRNA injection or triple gene (*Rad52*, *Ku70*, and *Polq*) siRNA injection (Additional file 1: Fig. S1F and S1G). Consequently, we opted to utilize the recently developed RNA editor, CasRX, to suppress the expression of these three factors. Three crRNAs were designed for each gene and co-injected with CasRX mRNA into mouse zygotes. RT-PCR analysis demonstrated that the expression of all three genes was substantially downregulated by CasRX, without additional interference with other indicated DNA repair genes (Fig. 1G and Additional file 1: Fig. S1H).

**Fig. 2:**
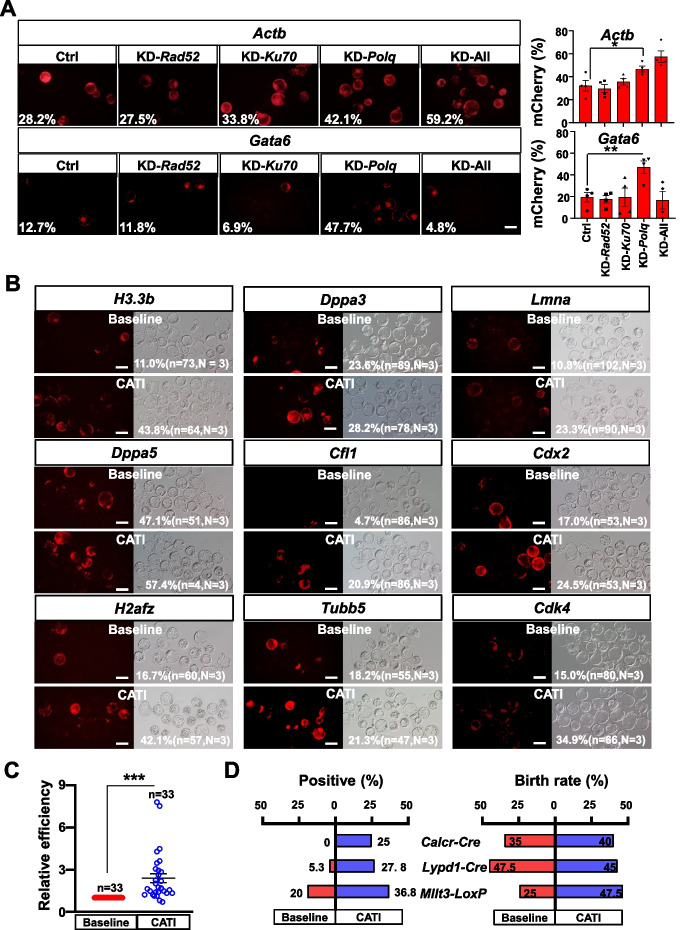
Downregulation of *Polq* enhances HDR efficiency*.* **A** Representative images display the knock-in efficiency at the *Actb* and *Gata6* loci. The ratio of mCherry-positive blastocysts indicates the knock-in efficiency. The “Ctrl” group signifies that no gene is knocked down, while “KD-*Rad52*,” “KD-*Ku70*,” “KD-*Polq*,” and “KD-All” represent the knockdown of *Rad52*, *Ku70*, *Polq* genes, and all three genes, respectively. The scale bar represents 100 μm. **B** Representative images of 9 genes integrated with mCherry using both baseline and CATI methods. HDR efficiency and the number of blastocysts are displayed. *N* denotes the number of replicates, and *n* indicates the total number of blastocysts obtained from all replicates. The scale bar is set at 100 μm. **C** A statistical analysis of the CATI method’s improvement in HDR efficiency for 11 loci in mouse embryos. A total of 33 experimental pairs were included in the analysis. A two-sided Student’s *t*-test was used for statistical evaluation, with ***p < 0.001 considered significant. Data are presented as mean ± standard error of the mean (s.e.m.). **D** A comparative analysis of targeting efficiency (left) and birth rate (right) for *Cre* or *LoxP* at 3 loci in mouse offspring, employing both baseline and CATI methods

Subsequently, we explored the impact of knocking down DNA repair-related factors on enhancing HDR efficiency. A well-established linearized donor DNA strategy was employed as baseline control [6, 7]. We designed the *Actb* and *Gata6* loci for mCherry integration by zygote injection of Cas9/sgRNA/donor DNA, with or without *Polq*-targeted crRNAs/CasRX. We discovered that the HDR efficiency significantly improved at both loci when *Polq* was knocked down. However, inconsistent results were observed in *Ku70*, *Rad52*, or triple knockdown groups between the *Actb* and *Gata6* loci (Fig. 2A). Interestingly, the triple knockdown exhibited distinct results at the two loci. The lower HDR efficiency at the *Gata6* locus was probably caused by impairments in embryo development. To verify this, we tested the triple knockdown while simultaneously conducting knock-in tests at two additional loci, *Dppa3* and *Cdx2*. As the results show, triple knockdown groups displayed a large reduction of blastocyst ratio (Additional file 1: Fig. S1I). These findings suggest that the triple knockdown approach carries inherent risks due to its apparent embryotoxicity. Consequently, we provided initial evidence that HDR efficiency could be enhanced in mouse embryos through *Polq* knockdown via CasRX. We termed this strategy CATI (CasRX-assisted targeted integration by homology-dependent repair). Although it is plausible that transient *Polq* knockdown might induce minimal adverse effects, we still conducted a *Polq* knockdown test to evaluate whether embryos develop normally. The results demonstrated that embryos with transient *Polq* knockdown developed into blastocysts comparably to controls (Additional file 1: Fig. S1J) and exhibited intact nuclear morphology without evident micronuclei formation (Additional file 1: Fig. S1K).

Subsequently, we selected 9 additional cleavage-stage expressed loci for mCherry integration to further examine the impact of *Polq* knockdown on HDR efficiency improvement (Fig. 2B). We conducted at least three independent replicates for each locus. Overall, all targets displayed an increased gene integration efficiency to varying degrees (Fig. 2B). Standardization analysis of all trials in the baseline group and CATI group across 11 loci revealed a 2.4-fold HDR efficiency increase in the CATI group with *Polq* knockdown (Fig. 2C). In addition to fluorescence reporter gene integration, we also designed *Cre* gene integration in two neuron-specific expressed genes (*Calcr*, *Lypd1*) and *LoxP* integration in one leukemia-related gene (*Mllt3*) (Additional file 1: Fig. S2A). Control and CATI group were designed as above; embryos were transferred into surrogates following zygote injection, and tail tissue from the resulting infants was used for genotypic analysis. Compared to the control group, the integration efficiency of these three loci was substantially increased in the CATI group (0 to 25% in *Calcr-cre*, 5.2 to 27.8% in *Lypd1-Cre*, and 20 to 36.8% in *Mllt3-LoxP*) (Fig. 2D and Additional file 3: Table S2). Furthermore, no significant differences were observed between the birth rates of the control and CATI groups (Fig. 2D and Additional file 1: Fig. S2D and S2F), indicating that transient *Polq* knockdown via CasRX is not detrimental to embryonic development. Germline transmission analysis revealed that the integrated DNA fragments were successfully transmitted to the next generation in all 7 tested founders (Additional file 1: Fig. S2B, S2C, S2E, and S2F and Fig. 2D and Additional file 3: Table S2). Southern blot and western blot analysis demonstrated the precise integration and expression of the *Cre* fragment (Additional file 1: Fig. S2G and S2H).

### Systematic evaluation of various methods for improving HDR efficiency

**Fig. 3:**
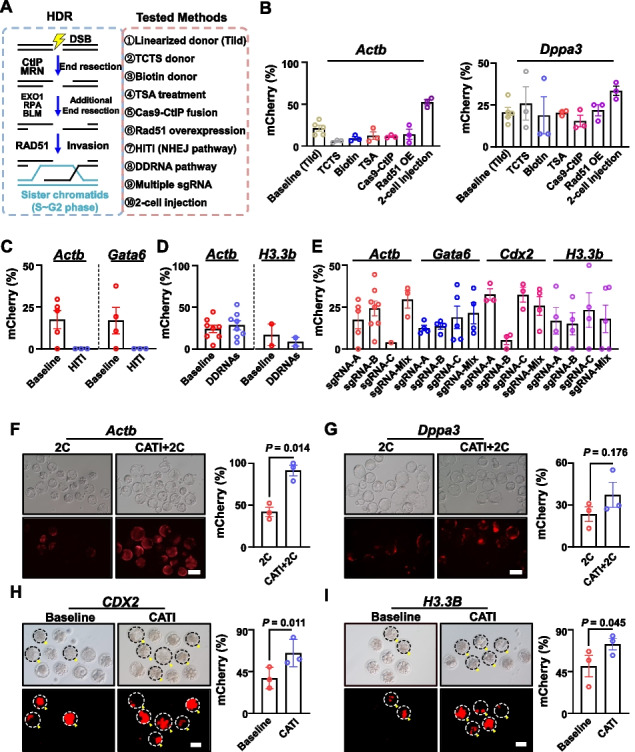
The CATI method demonstrates universality in enhancing HDR efficiency. **A** A schematic representation of HDR progression and the experimental approaches evaluated in this study. **B** Assessment of seven methods to improve knock-in efficiency at *Actb* locus (left) and *Dppa3* locus (right). The Tild method is performed as the baseline. TCTS indicates truncated Cas9-targeted sequence; Biotin indicates biotin modification on donors; TSA indicates the addition of TSA (Trichostatin A, HDAC inhibitor); Cas9-CtIP indicates fusion of Cas9 protein with *CtIP* functional domain; *Rad51* OE indicates the overexpression of Rad51 protein; 2-cell injection indicates the injection of regents at the 2-cell stage. Each dot indicates one biological replicate. **C** The HITI method failed to improve knock-in efficiency at the *Actb* and *Gata6* loci. Each dot indicates one biological replicate. **D** The DDRNA-based method failed to improve knock-in efficiency at the *Actb* and *H3.3b* loci. Each dot indicates one biological replicate. **E** Assessment of multiple sgRNA strategies to improve knock-in efficiency at the *Actb*, *Gata6*, *Cdx2*, and *H3.3* loci. Each dot indicates one biological replicate. **F** Representative images of *Actb* genes treated using the CATI + 2C approach. The scale bar represents 100 μm. Each dot corresponds to one biological replicate. **G** Representative images of *Dppa3* genes treated using the CATI + 2C approach. The scale bar represents 100 μm. Each dot corresponds to one biological replicate. **H** CATI enhances knock-in efficiency at the monkey *CDX2* locus. Left: representative images displaying mCherry-positive embryos. Right: a summary of experiments conducted at the *CDX2* locus. Paired *t*-test; *n* = 3 technical replicates per sample; error bars represent the standard error of the mean (SEM). The scale bar is set at 100 μm. Each dot corresponds to one biological replicate. **I** CATI enhances the knock-in efficiency at the monkey *H3.3* locus. Left: representative images displaying mCherry-positive embryos. Right: a summary of experiments conducted at the *H3.3* locus. Paired *t*-test; *n* = 3 technical replicates per sample; error bars represent the standard error of the mean (SEM). The scale bar is set at 100 μm. Each dot corresponds to one biological replicate

A series of strategies have been reported to enhance CRISPR/Cas9-based HDR efficiency in cells and embryos across different organisms. We have summarized 10 distinct methods reported for HDR efficiency improvement in cultured cells or embryos (Fig. 3A). These methods include using donors with truncated Cas9-targeted sequence (referred to as TCTS) [35], using donors with biotin modification [14], Trichostatin A (TSA) treatment [24], fusion of CtIP functional domain to Cas9 protein [36], overexpression of Rad51 [25], microinjection at 2-cell stage [14], NHEJ-based homology-independent targeted integration (HITI) [11], co-injection of DDRNAs (DNA damage response RNAs) [37, 38], and utilization of multiple sgRNAs [39] (Additional file 1: Fig. S3A).

We performed a comprehensive assessment of the repeatability or the universality of these methods in mouse embryos. For each method, we performed 3 individual repeats for mCherry integration in at least 2 loci. Microinjection of Cas9/sgRNA/DNA donor in zygotes is still referred to as the baseline control group. We discovered that most of these methods did not exhibit efficiency improvement beyond the baseline control group, except for the 2-cell injection approach, which demonstrated a significant increase in HDR efficiency from 21.2 to 52.3% (*Actb*) and 20.6 to 33.5% (*Dppa3*) (Fig. 3B–E).

To explore the potential synergistic effect of combining our CATI method with the 2-cell injection method for enhancing embryonic HDR efficiency, we carried out 2-cell stage injections at the *Actb* and *Dppa3* loci, with or without *Polq* knockdown via CATI. Remarkably, we further elevated the HDR efficiency at the *Actb* locus from 42.2 to 91.3% (Fig. 3F). The HDR efficiency at the *Dppa3* locus was also increased from 23.5 to 37.4% (Fig. 3G). Concurrently, HDR efficiency did not improve when combining 2-cell stage injection with *Rad52* or *Ku70* knockdown (Additional file 1: Fig. S3B). Together, we believe that this CATI + 2C (CATI in conjunction with 2-cell injection) approach demonstrates the highest efficiency to date and recommend it as a superior strategy for knock-in experiments in mouse embryos.

### CATI is effective at improving HDR efficiency in monkey embryo editing

Non-human primate models are of great value in neuroscience and biomedical studies [40–45]. However, HDR efficiency improvement in monkey embryos has been rarely studied. We believe a highly efficient HDR method in monkey embryo editing has significant implications for generating monkey models. Thus, we investigated whether effective knock-in methods in mouse embryos are suitable for monkey embryos. We first tested the 2-cell injection method in monkey embryos. The *CDX2* locus was designed for mCherry integration by zygote injection of Cas9/sgRNA/linearized DNA donor in monkey zygotes and 2-cell embryos. We found that the mCherry fluorescence-positive rate was much lower in the 2-cell injection group compared with the zygote injection group (Additional file 1: Fig. S3C and S3D). Considering that monkey zygotic genome activation occurs in the 4–8 cell stage [46–48], we further tested whether 4-cell injection would benefit monkey HDR efficiency. Surprisingly, we found that the HDR efficiency in the 4-cell injection group is the lowest among the zygote, 2-cell, and 4-cell injection groups (Additional file 1: Fig. S3C and S3D). One potential explanation for this observation could be that the 4-cell monkey embryo exhibits a truncated G2 phase, during which HDR takes place. Additionally, it is important to note that zygotic genome activation occurs at the 2-cell stage in mice, whereas in primates, it transpires at the 8-cell stage. Consequently, in 4-cell stage monkey embryos, the chromatin accessibility might not yet be fully established. These results indicate that the mouse HDR effective strategy of 2-cell injection is not suitable for monkeys.

Next, we assessed the efficacy of our developed CATI strategy for enhancing HDR efficiency in monkey embryos. We selected the *CDX2* locus for mCherry integration, and monkey zygote injections were performed with Cas9/sgRNA/linearized DNA donor, either with or without *Polq*-targeted crRNA/CasRX, to create the control and CATI groups. We conducted three independent trials to gather statistical data for analysis. Excitingly, the CATI group exhibited a substantial improvement in HDR efficiency at the *CDX2* locus (37.9% vs. 64.9%) (Fig. 3H). Further tests at another locus, *H3.3B*, also confirmed the effectiveness of the CATI strategy in monkey embryo editing (50.8% vs. 74.5%) (Fig. 3I). Consequently, we demonstrated that our MMEJ regulation-based CATI strategy is universally applicable for enhancing HDR efficiency in both rodent and primate embryo editing. Although we only tested the CATI strategy in monkey early embryonic expressed genes, we believed that CATI will be effective in other post-implantation expressed genes and play a crucial role in future monkey model generation.

### CATI enhances the efficiency of ssODN-mediated nucleotide replacement

**Fig. 4:**
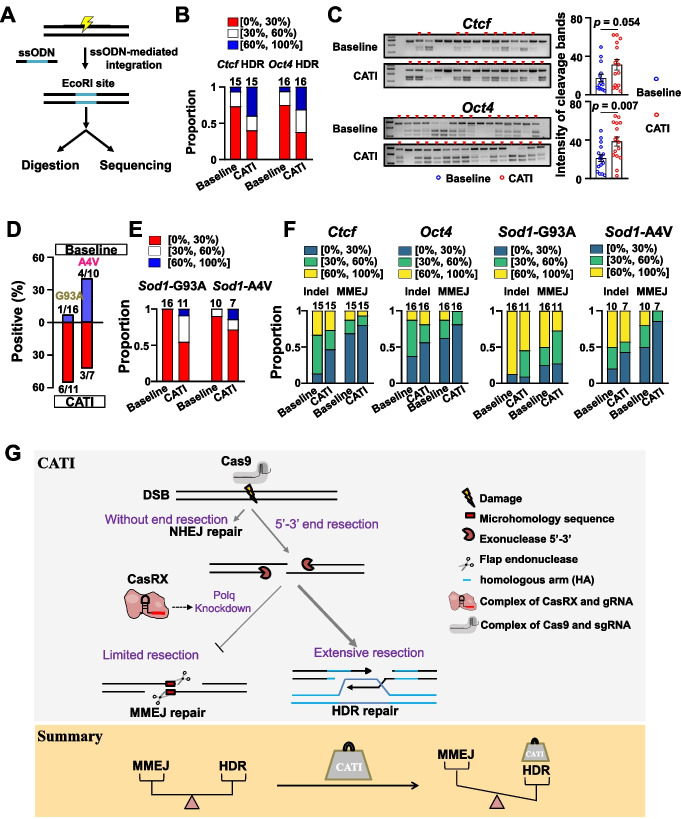
The CATI method enhances the efficiency of nucleotide replacement. **A** Experimental flow diagram illustrating the steps involved. Following ssODN-mediated integration of the EcoRI site, we conducted restriction enzyme site analysis and Sanger sequencing tests. The light symbol represents CRISPR/Cas9-mediated DSBs, while blue lines indicate the EcoRI site. **B** Distribution of HDR efficiency percentages for the *Oct4* and *Ctcf* loci using baseline and CATI methods. Red rectangles represent low efficiency (0–30%), white rectangles denote intermediate efficiency (30–60%), and blue rectangles signify high efficiency (60–100%). The “number” refers to the total embryos sequenced and analyzed. **C** Restriction enzyme site analysis demonstrated an increased HDR efficiency using the CATI method. Gel images (left) and quantitative analysis (right) reveal that the CATI approach is more effective than the baseline method at the Oct4 and Ctcf loci. A two-sided Student’s *t*-test was employed for statistical analysis. Data are presented as mean ± standard error of the mean (s.e.m.). **D** Comparison of the positive rate in mouse offspring for G93A and A4V mutation mimicry using both baseline and CATI methods. **E** Distribution of HDR efficiency percentages for G93A and A4V mutations at Sod1 locus using baseline and CATI methods. Pink rectangles represent low efficiency (0–30%), green rectangles denote intermediate efficiency (30–60%), and yellow rectangles signify high efficiency (60–100%). The number refers to the total number of embryos sequenced and analyzed. **F** Proportion of indel and MMEJ events across the four tested loci. Blue rectangles represent low efficiency (0–30%), green rectangles represent middle efficiency (30–60%), and yellow rectangles represent high efficiency (60–100%). The number refers to the total number of embryos or mice sequenced and analyzed. **G** Schematic diagram of CATI strategy enhancing gene knock-in efficiency

Single-strand oligonucleotide (ssODN) mediated HDR for repairing CRISPR/Cas9-induced DSBs has been regarded as a promising approach in germ-cell gene therapy. We explored the potential improvement in ssODN-mediated HDR using the CATI strategy. Initially, EcoRI restriction enzyme sites were designed in the *Oct4* and *Ctcf* loci through ssODN-mediated HDR. Cas9/sgRNA/ssODN (control group) or Cas9/sgRNA/ssODN/crRNA/CasRX (CATI group) were injected into mouse embryos, and the resulting blastocysts were utilized for genotyping and restriction endonuclease digestion analysis (Fig. 4A). We discovered that the HDR efficiency of both loci was significantly enhanced in the CATI group compared to the control group (*Ctcf*: 17.1% vs. 40.0%, *Oct4*: 22.3% vs. 43.1%, Fig. 4B). The cleavage bands and the quantified data in the restriction endonuclease digestion analysis further confirmed these findings (Fig. 4C).

We then proceeded to utilize CATI to simulate clinically relevant diseases in mouse offspring. We chose G93A and A4V mutations in the *Sod1* gene to model amyotrophic lateral sclerosis (ALS) disease-related mutations [49, 50]. Employing a similar strategy as above, we generated mouse offspring by transferring injected zygotes into surrogate mothers. Genotype analysis of the resulting mouse offspring revealed that 1 out of 16 (6.25%) mice was G93A positive in the control group, while 6 out of 11 (54.55%) mice were G93A positive in the CATI group. For the A4V mutation, 4 out of 10 (40%) and 3 out of 7 (42.86%) mice were genotyped as positive in the control and CATI groups, respectively (Fig. 4D). HDR efficiency analysis of the positive offspring demonstrated that both the G93A and A4V loci in the CATI group exhibited substantially higher HDR efficiency compared to the control group (G93A: 22.0% vs. 48.8%, A4V: 23.5% vs. 40.8%) (Fig. 4E). Notably, we observed a decrease in indel frequencies at the target sites in the CATI group compared to the control group, and further analysis indicated that this decrease might result from a reduction in MMEJ repair (Fig. 4F). We believe this reduction in indel frequency could benefit genome stability and integrity [51, 52].

Subsequently, we employed CATI to introduce a clinically relevant mutation in monkey embryos. We designed a clinical R178Q mutation for integration into the monkey *CDKL5* locus using an ssODN (Additional file 1: Fig. S4A). We discovered that 2/9 and 5/9 embryos were R178Q positive in the control and CATI groups, respectively (Additional file 1: Fig. S4B). The highest efficiency among the positive embryos increased from 5 to 26% when comparing the control and CATI groups (Additional file 1: Fig. S4C). A reduction in the MMEJ ratio was also observed in this monkey CATI group (Additional file 1: Fig. S4C). These findings suggest that CATI offers both efficiency and potential safety advantages for germ cell gene correction in ssODN-mediated HDR.

### The CATI approach did not exacerbate any CRISPR-induced side effects in the tested samples

Off-target effects and safety are critical concerns in the development of gene editing technology. To thoroughly assess the safety of the CATI method, we conducted a rigorous off-target analysis experiment, GOTI, at the whole genome level [53]. In brief, knock-in reagents and *Cre* mRNA were injected into one blastomere of mTmG transgenic mouse-derived 2-cell stage embryos. The injected blastomere developed into tissues expressing RFP, while the uninjected blastomere formed tissues expressing GFP. The RFP and GFP cells were sorted separately and used for whole-genome sequencing and comparative analysis (Additional file 1: Fig. S4D).

We selected the *Lypd1-Cre* locus for the GOTI experiment. Blastomere injections of Cas9/sgRNA/donor DNA/*Cre* with or without *Polq*-targeted crRNAs/CasRX were designed as the control and CATI groups. We obtained two E14.5 fetuses in each group. GFP- and RFP-labeled cells dissected from the resulting fetuses were sorted for whole-genome sequencing. We first validated the on-target effect in RFP-expressing cells using Sanger sequencing and whole-genome sequencing. We observed that both the control and CATI groups exhibited high targeting efficiency (Additional file 1: Fig. S4E and S4F). Subsequently, we examined the potential off-target effects in the control and CATI groups by analyzing the number of de novo SNPs in RFP- and GFP-labeled cells from each fetus using the GOTI analysis method [53]. We discovered 46 and 20 de novo SNPs in control samples, and there were 70 and 30 de novo SNPs in CATI samples (Additional file 1: Fig. S4G). The indel numbers also showed no significant differences between these two groups (Additional file 1: Fig. S4H). Moreover, we detected no nucleotide bias among the mutation types (Additional file 1: Fig. S4I), suggesting that *Polq* knockdown may not induce spontaneous mutations as base editors. Taking into account previous reports indicating that *Polq* inhibition leads to genomic instability, we analyzed the frequencies of genomic rearrangement between the CATI and control groups and found no statistically significant difference (Additional file 1: Fig. S4J and S4K).

Subsequently, we identified random insertion events by detecting the presence of exogenous donor sequences, such as left/right homology arms and mCherry, integrated into non-targeted genomic loci (Additional file 1: Fig. S4L). We meticulously analyzed whole-genome sequencing (WGS) data obtained from GOTI experiments. To ensure the accuracy of our findings, we applied a stringent cutoff value requiring discordant reads to map more than 10 times. The results demonstrate that the number of integration events in CATI samples is comparable to that in control samples (Additional file 1: Fig. S4M). This evidence suggests that, relative to the conventional knock-in approach, the CATI method does not introduce a higher rate of random integrations in the examined samples.

Moreover, large fragment deletions caused by CRISPR-mediated cleavage represent a significant concern that must be addressed in clinical applications. To elucidate the genetic alterations resulting from CATI treatment, we amplified approximately 5-kb regions surrounding the stop codons of the *Cdx2* and *Gata6* loci from pools of five embryos edited using both Tild and CATI methods. We subsequently sequenced the PCR products using the PacBio platform. Our analysis revealed substantial deletions in read coverage around the cut sites (stop codons) for both loci. Notably, we observed a lower proportion of lost coverage in the CATI group at both loci (Cdx2: 33.25% and 2.89% vs. 0.96% and 12.07%; Gata6: 16.14% and 6.94% vs. 0.54% and 1.10%) (Additional file 1: Fig. S4N). These findings demonstrate that the CATI method is safe for gene editing, without introducing additional mutations and genomic rearrangements.

## Discussion

The application of CRISPR/Cas9 in embryo gene editing has significantly advanced the fields of model generation and germ-cell therapy trials. Previously, NHEJ was considered as a major pathway for nuclease-mediated DNA repair in embryo gene editing [31]. As a result, several studies focused on improving HDR efficiency by inhibiting NHEJ [22, 23]. However, the repeatability of this NHEJ inhibition strategy has been challenged by subsequent research [25, 31]. Through genotyping analysis of over 1400 mouse embryos with 88 individual sgRNA/Cas9 mRNA injections, we were intrigued to find that MMEJ played a substantial role in DSB repair in mouse embryo gene editing, with some loci even exhibiting a higher proportion of MMEJ-mediated repair events compared to those mediated by NHEJ. This finding could explain why previous NHEJ regulation-based HDR efficiency improvement strategies were not repeatable across different studies and provided a crucial foundation for our MMEJ regulation-based HDR efficiency improvement method, CATI (Fig. 4G).

Numerous studies have reported HDR efficiency improvement methods across various systems and organisms. A comprehensive evaluation and comparison are essential within the mouse embryo editing system. We discovered that most of the methods exhibited no effect in enhancing mouse embryo HDR efficiency. One reason is that many of these methods were developed using cultured cells. Another significant factor is that the control baseline employed in our study already represents an improved and efficient approach. Conversely, this further highlights the effectiveness and importance of our CATI method. To our knowledge, the CATI + 2C method demonstrates higher HDR efficiency than all other methods. Consequently, HDR efficiency in mouse model generation will likely not be a major concern in the future.

Gene editing in non-human primate embryos has been investigated for several years, resulting in the recent generation of a series of knockout monkeys [45, 54–57]. However, research on HDR-mediated precise gene modification in monkey models remains limited [58, 59]. Based on our observations, the overall sgRNA targeting efficiency in monkey embryos is lower than that in mouse embryos. Additionally, transcriptome analysis of monkey embryos has demonstrated lower expression levels of HDR repair-related genes in preimplantation monkey embryos compared to their mouse counterparts [60]. Consequently, there is a significant need for an efficient HDR method in generating monkey models. Our MMEJ regulation-based CATI exhibits a clear advantage in HDR editing of monkey embryos, making it a preferred choice for future knock-in monkey model generation.

In addition to generating animal models, gene editing technology offers significant potential for both germ cell and somatic cell gene therapy [2, 61]. While base editors have seen extensive use in gene correction for mouse and human zygotes, their applicability remains limited for a substantial proportion of gene mutations [62, 63]. Recent advancements in prime editing have revealed efficiency constraints in germ cells [62, 64]. Our research indicates that the CATI method can enhance the efficiency of ssODN-mediated HDR in both mouse and primate embryos. These findings underscore the promise of CATI for germ-cell gene therapy applications. Further investigation into the correction of clinical mutations utilizing CATI is warranted in future studies.

Safety remains a paramount concern in gene editing technology, particularly in the context of gene therapy. Previous studies have shown that increased *Polq* expression can heighten the risk of random integration [65, 66], while complete *Polq* deletion may lead to genome instability in cultured cells [67]. Consequently, we posit that CasRX-mediated knockdown represents an optimal strategy for modulating *Polq* expression, given the transient nature of crRNAs/CasRX during embryo editing. Our thorough off-target analysis using the GOTI method demonstrated that the CATI group exhibited no significant impact on off-target effects and random integration of exogenous DNA compared to the control group. Additionally, the CATI method resulted in fewer large fragment deletions compared to the conventional approach at the loci we examined. As such, the CATI method holds considerable promise for applications in animal generation and germ-cell gene therapy.

## Conclusions

We would like to emphasize that current methodologies, which rely on edited sequences for differentiating repair patterns, may not be sufficiently rigorous, as they are unable to distinguish uncut DNA from a flawless DNA lesion repaired via the HDR, NHEJ, or MMEJ pathways. Nevertheless, our conclusions regarding MMEJ in embryos remain valid, as the ratio of NHEJ and MMEJ is unlikely to be significantly affected by a small number of uncut DNA events when using sgRNAs with high editing performances. However, more precise and user-friendly techniques for DNA repair identification are still desirable. Moreover, CATI offers an alternative, reproducible approach for gene integration in embryos. Yet, for some sites, gene integration frequencies have not seen substantial improvements, highlighting the complexity of the embryo DNA repair pathway. We anticipate the development of more advanced approaches for gene integration in the future.

## Methods

### Mice

B6D2F1 (C57BL/6 J × DBA/2N) mice (3 or 8 weeks old) were used for zygote collection. ICR females were used as surrogates. The use and care of animals complied with the guideline of the Center for Excellence in Brain Science and Intelligence Technology, Chinese Academy of Sciences.

### Monkey

Healthy female cynomolgus monkeys (*Macaca fascicularis*) with regular menstrual cycles were selected for this study. All animals were housed in the sunny room. The use and care of animals complied with the guideline of the Center for Excellent in Brain Science and Intelligence Technology, Chinese Academy of Science, which approved the ethics application (ION-2019022).

### Construction of linearized donors

To construct Tild donor for mouse *Actb*/*Dppa5*/*Cfl1*/*H3.3b*/*Cdx2*/*H2afZ*/*Cdk4*/*Lmna*/*Dppa3*/*Gata6*/*Tubb5* gene and monkey *CDX2*/*H3.3B* gene, we take the mouse *Actb* gene as an example to expand the detailed description. For the Tild donor (transgene DNA sandwiched by different lengths of homology arm) for the *Actb* gene, an *Actb*-HR-donor vector containing (~ 800 bp) HAL-p2A-*mCherry*-(~ 800) HAR was linearized with PCR-amplification, and PCR production was purified with PCR Extraction Kit (Magen, D2121-03). To construct a Tild donor for the mouse *Calcr*/*Lypd1* gene, we take the mouse *Calcr* gene as an example to expand the detailed description. An *Calcr*-HR-donor vector containing (~ 800 bp) HAL-p2A-Cre-(~ 800) HAR was linearized with PCR amplification, and PCR production was purified as described above. To construct a Tild donor for the mouse *Mllt3* gene, an *Mllt3*-HR-donor vector containing (~ 800 bp) HAL-*LoxP*-exon3-*LoxP*-(~ 800) HAR was linearized with PCR amplification, and PCR production was purified as described above.

### Construction of modified linearized donors and ssODN donor

For ssODN donor (Additional file 4: Table S3), 5′-phosphorylation and phosphorothioate-modified donor oligos for mouse *Oct4*/*Ctcf*/*Sod1* genes were synthesized (GenScript) and diluted to 1 μg/μl. For biotin-modified linearized donors, the primers were synthesized with 5′-biotin (GenScript). The donor was amplified by 5′-biotin-modified primers (Additional file 5: Table S4).

### Production of Cas9/Cas9-msa/CasRX/Rad51 mRNA and sgRNA

T7 promoter was added to the N terminus of the Cas9/Cas9-msa/CasRX/*Rad51* coding region by PCR amplification, using indicated primer (Additional file 4: Table S3). T7-Cas9/Cas9-msa/CasRX/*Rad51* PCR production was purified and used as the template for in vitro transcription (IVT) using the mMESSAGE mMACHINE T7 ULTRA kit (Invitrogen, AM1345). T7 promoter was added to the sgRNA template by PCR amplification, using primers listed in Additional file 4: Table S3. The T7-sgRNA PCR product was purified and used as the template for IVT using the MEGA shortscript T7 kit (Invitrogen, AM1354). Both the mRNA and the sgRNAs were purified using a MEGA clear kit (Invitrogen, AM1908) and eluted in RNase-free water.

### Preparation of injection mixtures

All injection mixtures were prepared in a final volume of 10 μl according to the following protocol. Using RNase-free water, reagents, and consumables. Cas9, Cas9-msa, Cas9-CtIP, CasRX and *Rad51* (final concentration 100 ng/μl); Cas9-sgRNA, CasRX-gRNA and DDRNA (final concentration 50 ng/μl per synthesized RNA); linearized donor described as Tild-CRISPR, linearized donor with TCTS and linearized donor with HITI (final concentration 100 ng/μl), linearized donor with 5′-biotin-modified (final concentration 20 ng/μl), and SSODN donor (final concentration 30 ng/μl). For Tild-CRISPR-mediated experiment, Cas9 mRNA, site-specific sgRNA, and Tild-CRISPR donor were mixed; for Tild-TCTS-CRISPR-mediated experiment, Cas9 mRNA, site-specific sgRNA, and linearized donor with TCTS were mixed; for Tild-HITI-CRISPR-mediated experiment, Cas9 mRNA, site-specific sgRNA, and linearized donor with HITI were mixed; for CtIP-mediated experiment, Cas9-CtIP mRNA, site-specific sgRNA, and Tild-CRISPR donor were mixed; for 5′-biotin modification-mediated experiment, Cas9-msa mRNA, site-specific sgRNA, and linearized donor with 5′-biotin-modified were mixed; for *Rad51* overexpression-mediated experiment, Cas9 mRNA, site-specific sgRNA, Tild-CRISPR donor, and *Rad51* mRNA were mixed; for multiple sgRNA-mediated experiment, Cas9 mRNA, three sgRNA for one target gene, and Tild-CRISPR donor were mixed; for CATI-mediated experiment, Cas9 mRNA, site-specific Cas9-sgRNA, Tild-CRISPR donor, CasRX mRNA, and CasRX-sgRNA were mixed (three sgRNA for mouse gene and four sgRNA for monkey gene).

### Mouse embryo injection, embryo culturing, and embryo transplantation

For mice gene editing, superovulated B6D2F1 (C57BL/6 J × DBA/2N) female mice (3 or 8 weeks old) were injected with pregnant mare serum gonadotropin (PMSG, 5 IU/mouse for 3-week-old mouse, 10 IU/mouse for 8-week-old mouse), followed by human chorionic gonadotropin (hCG, 5 IU/mouse for 3-week-old mouse, 10 IU/mouse for 8-week-old mouse) 48 h later and then paired with adult B6D2F1 males.

For zygote injection, zygotes were collected from oviducts at 20 h post-hCG injection. Mixtures were injected into the cytoplasm of fertilized eggs with well-recognized pronuclei at the volume of 1–3 pl. For 2-cell embryo injection, embryos were collected at the 1-cell stage and cultured until 45–48 h post-hCG injection, then the mixture was injected into the cytoplasm of one or two blastomeres of 2-cell embryos at the volume of 1–3 pl. Microinjection was performed in a droplet of M2 medium containing 5 μg/ml cytochalasin B (CB) using a Piezo-driven micromanipulator (Prime Tech). Then, the injected embryos were cultured in a KSOM medium with amino acids.

For Trichostatin A (TSA) treatment, the injected zygotes were cultured in KSOM medium with 10 nM TSA for 5–6 h and then transferred to fresh KSOM medium with amino acids at 37 °C in 5% CO_2_. We collected E4.5 blastocyst for fluorescence observation or genotyping analysis.

For knock-in mice generation, the injected embryos were cultured in KSOM medium with amino acids at 37 °C under 5% CO^2^ for 2 h and then transferred into the oviducts of pseudopregnant ICR females at 0.5 dpc (20 embryos/surrogate).

### Monkey oocytes collection, ICSI, embryo injection, and embryo culturing

For monkey oocyte collection, procedures of intracytoplasmic sperm injection (ICSI), embryo injection and embryo culturing, have been mentioned in a previous publication [68]. Briefly, from day 3 of the menstrual cycle, healthy female cynomolgus monkeys began to receive 25 IU recombinant human follitropin twice daily for 7–8 days. On day 11 of the menstrual cycle, 1000 IU human chorionic gonadotropin was injected, and oocytes were aspirated from the ovarian follicles 36 h later. MII-arrested oocytes were selected for ICSI, fertilization of which was confirmed 6 h later by the presence of two pronuclei. Then, the fertilized eggs were injected with the regent mixture (1 ~ 3 pl).

For 2-cell or 4-cell embryo injection, each blastomere was injected with the mixture. After the microinjection, embryos were cultured in pre-equilibrated HECM-9 at 37 °C with 5% CO_2_ until the 8-cell stage. Then, embryos were transferred to HECM-9 + 5% FBS medium for culture. Until reaching the blastocyst stage, embryos were used for fluorescence observation or genotyping analysis.

### Embryo and mouse genotyping tests

For embryo genotyping analysis, single embryos were picked up and transferred directly into PCR tubes with 5 μl lysis buffer from Mouse Direct PCR Kit. The samples were incubated at 56 °C for 30 min and heat inactivate proteinase K at 95 °C for 10 min. genome DNA amplification was performed using random primer sets (Additional file 4: Table S3). The DNA was amplified by PCR in a 30-μl reaction mixture composed of 0.5 μl rTaq, 10 μl random primer, 1.5 μl 2.5 mM dNTP mix, 3 μl 10 × buffer, and sterile distilled water added to bring the total reaction volume to 30 μl. The PCR cycling parameters were 1 cycle of (95 °C for 5 min), 50 cycles of (95 °C for 1 min; 37 °C for 2 min; 55 °C for 4 min), and 1 cycle of (55 °C for 4 min). Secondary PCR was performed using 1 μl random PCR product and indicated primers (Additional file 4: Table S3). The 50-μl reaction mixture composed of 1 μl KOD-FX DNA polymerase, 25 μl KOD Buffer, 10 μl dNTP mix, 1.5 μl 10 mM forward and reverse primers, 1 μl DNA template from random PCR product, and sterile distilled water was added to bring the total reaction volume to 50 μl. Using the touchdown PCR method, the cycling parameters were 1 cycle of (94 °C for 2 min), 10 cycles of (98 °C for 10 s; 65 °C for 15 s; 68 °C for 50 s), 34 cycles of (98 °C for 10 s; 55 °C for 15 s; 68 °C for 50 s), and 1 cycle of (68 °C for 5 min). The specific PCR products were gel-purified and sequenced. For mouse genotyping analysis, mouse genomic DNA was extracted from the samples using the Mouse Direct PCR Kit. PCR amplification was performed using primers designed to amplify the correctly targeted junctions (Additional file 4: Table S3). KOD-FX DNA polymerase was used to amplify specific DNA sequences, and PCR was carried out in the same reaction mixture as the embryo and products were gel-purified and sequenced.

### Southern blot

The 25 μg of genomic DNAs from *Lypd1*-p2A-Cre mice was digested with Nde I. The 25 μg of genomic DNAs from *Calcr*-p2A-Cre mice was digested with Pst I. The digested genomic DNA was then separated on a 0.8% agarose gel and transferred to a Nylon transfer membrane, positively charged (GE Healthcare, RPN303B). Southern blot analysis was performed using the DIG-labeled system, and the membranes were hybridized with an internal Cre probe (0.5 kb): aatgcttctgtccgtttgccggtcgtgggcggcatggtgcaagttgaataaccggaaatggtttcccgcagaacctgaagatgttcgcgattatcttctatatcttcaggcgcgcggtctggcagtaaaaactatccagcaacatttgggccagctaaacatgcttcatcgtcggtccgggctgccacgaccaagtgacagcaatgctgtttcactggttatgcggcggatccgaaaagaaaacgttgatgccggtgaacgtgcaaaacaggctctagcgttcgaacgcactgatttcgaccaggttcgttcactcatggaaaatagcgatcgctgccaggatatacgtaatctggcatttctggggattgcttataacaccctgttacgtatagccgaaattgccaggatcagggttaaagatatctcacgtactgacggtgggagaatgttaatccatattggcagaacgaaaacgctggttagcaccgcaggtgtag. The Cre probe was amplified with the PCR DIG Probe Synthesis Kit (Roche). The membranes were detected with a DIG-High Prime DNA Labeling and Detection Starter Kit II (Roche, Germany). For *Lypd1*-p2A-Cre mice, the internal Cre probe expected fragment size: WT = N/A, targeted = 2.95 kb. For *Calcr*-p2A-Cre mice, the internal Cre probe expected fragment size: WT = N/A, targeted = 5.5 kb.

### Library preparation of Smart-seq2

Six hours after injecting regents of PBS, Cas9 only, Cas9/sgRNA, and Cas9/sgRNA/donor as different groups, five embryos per group were collected into one tube for mRNA amplification which was performed following the manufacturer’s instructions (Vazyme, N712-03) with 18 amplification cycles. cDNA concentration was determined by Qubit Flex Fluorometer (Thermo Fisher Scientific) and fragment size distributions were verified by Agilent Bioanalyzer 2100. For library preparation, TruePrep DNA Library Prep Kit V2 for Illumina (Vazyme, TD503) was used following the manufacturer’s instructions.

### RT-PCR

SYBR-qPCR was performed using a ChamQ SYBR Color qPCR Master Mix (Vazyme, Q421-02). Gene expression levels were measured with Roche 480II Real-Time PCR System (Roche). Primers are listed in Additional file 4: Table S3.

### Overview of GOTI

In this study, a mixture of Cre, Cas9 mRNA, genome targeting sgRNA, CasRX mRNA, and sgRNA (targeting *Polq* as the experimental group and scramble sgRNA as the control group) into one blastomere of a 2-cell mouse embryo, derived from mTmG male mice mating with wild-type female mice. The action of Cre, injected into one of two cells, is expected to generate a chimeric embryo labeled with both GFP and RFP. When the chimeric embryo reaches embryonic day 14.5 (E14.5), it is minced into small pieces and digested into a single-cell suspension. We collected GFP^+^ and RFP^+^ cells by fluorescence-activated cell sorting (FACS), respectively. Next, the two population cells are independently processed for whole-genome sequence (WGS), which are subsequently processed by standard pipeline as reported.

### RNA-seq analysis

RNA-seq reads were quality-checked, trimmed, and aligned to reference genome mm9 using STAR. Reads were counted for feature counts, and data was normalized utilizing DEseq2 in R/Bioconductor. All other RNA-seq analyses and statistics were performed in R/Bioconductor utilizing custom R scripts.

### Analysis of editing efficiency and identification of repair patterns

Collected embryos and cells were lysed for Sanger sequencing followed by ICE v2 CRISPR Analysis, reliability of which has been confirmed by several studies [69] (https://www.synthego.com/publications). To analyze HDR efficiency, we should also provide HDR donor sequences. After the output of editing results, we analyzed all shown events per sample. The results of indel and HDR are easy to obtain. However, ICE v2 does not separate MMEJ and NHEJ from indel. To sort out NHEJ and MMEJ, we manually curated all editing patterns following the rule of previous studies as described in the manuscript. Then, the frequencies of indel, HDR, MMEJ, and NHEJ were calculated by averaging corresponding values of sequenced embryos, edited embryos, and knock-in embryos.

### GOTI pipeline

After quality checking and trimming out adapters of raw sequencing reads, qualified reads are then mapped to the reference genome (mm10). Next, the mapped alignment files are sorted and duplicates marked. The off-target SNVs and indels are identified by comparing the GFP^+^ cells with RFP^+^ cells using three variant calling algorithms (Mutect2, Lofreq, and Strelka2 for SNV detection; Mutect2, Scalpel, and Strelka2 for indel detection). We considered the overlap of the three algorithms of SNVs or indels as the true variants.

### Random integration analysis

We used WGS data from the GOTI experiment to analyze random integration. The paired-end reads were mapped to the mm10 reference by BWA, and then chimeric reads were extracted and break pointes were predicted from chimeric reads aligned to both the mm10 and the donor sequence. In our study, we used mapping quality and counts of reference-donor chimeric DNA fragments for random integration break-point calling. We defined breakpoints with a chimeric read count ≥ 10 and 20 as true signals.

### Large deletion detection

Five embryos were picked up and transferred directly into PCR tubes with 5 μl lysis Buffer from Mouse Direct PCR Kit. The samples were incubated at 56 °C for 30 min and heat inactivate proteinase K at 95 °C for 10 min. The DNA was directly amplified by KOD-FX (TOYOBO, 1,256,001) using a primer (Additional file 4: Table S3). The specific PCR products were purified using a kit (Magen, D2121-03) according to the manufacturer’s instructions and then analyzed by PacBio Sanger sequencing. Pacbio-sequenced PCR products were classified by Cutadapt (v1.18) and then aligned to references by Minimap2 (v2.24-r1122). Successful alignment results were filtered by Samtools (v1.16) according to the flag. Finally, genome reads coverage was calculated with the samtools depth command.

### Statistical analysis

All statistical analyses were performed using Prism 8 (GraphPad), except RNA-seq data analyses which were performed in R/Bioconductor. Details of individual tests are outlined within each figure and figure legend, including the number and type of replication performed (*n*). All statistics are calculated by a two-tailed Student’s *t* test, and all graphs display mean ± SEM. No statistical methods were used to predetermine the sample size. Experimenters were blind during all behavioral tests.

## Supplementary Information

**Additional file 1: Fig. S1.** Evaluation of editing efficiency, NHEJ/MMEJ ratio, DNA repair-related gene expression, and knockdown approaches in embryos. **Fig. S2.** Integration analysis of Cre knock-in at Lypd1 and Calcr loci. **Fig. S3.** Two-cell strategy test in mouse and monkey embryos. **Fig. S4.** CATI application in nucleotide replacement of monkey CDKL5 locus and GOTI experiment for off-target analysis.

**Additional file 2: Table S1.** An in-depth analysis of the DNA repair patterns associated with 88 sgRNA targets was conducted.

**Additional file 3: Table S2.** Summary of mice line generated by CATI method, Related to Fig. 2.

**Additional file 4: Table S3.** Primers/Oligos used in this study.

**Additional file 5: Table S4.** Donor DNA used in this study, related to Method.

**Additional file 6.** Review history.

## Data Availability

RNA-seq accession used in this study has been deposited in the SRA under accession number PRJNA980391 [70]; WGS accession used in this study has been deposited in the SRA under accession number PRJNA980392 [70]; PacBio accession used in this study has been deposited in the SRA under accession number PRJNA980897 [70].
