# Supplementary material for: CATI: an efficient gene integration method for rodent and primate embryos by MMEJ suppression
Source: Genome Biol. 2023 Jun 23;24:146. doi: 10.1186/s13059-023-02987-w (PMC10288798; doi:10.1186/s13059-023-02987-w)
Supplement: Supplementary file 1 — Additional file 1: Fig. S1. Evaluation of editing efficiency, NHEJ/MMEJ ratio, DNA repair-related gene expression, and knockdown approaches in embryos. Fig. S2. Integration analysis of Cre knock-in at Lypd1 and Calcr loci. Fig. S3. Two-cell strategy test in mouse and monkey embryos. Fig. S4. CATI application in nucleotide replacement of monkey CDKL5 locus and GOTI experiment for off-target analysis. [file 13059_2023_2987_MOESM1_ESM.docx]

**SUPPLEMENTARY INFORMATION**

**An Efficient Gene Integration Method in Rodent and Primate Embryos**

**by MMEJ suppression**

Hongyu Chen, Xingchen Liu, Lanxin Li, Qingtong Tan, Shiyan Li, Li Li, Chunyang Li, Jiqiang Fu, Yong Lu, Yan Wang, Yidi Sun, Zhen-Ge Luo, Zongyang Lu, Qiang Sun, Zhen Liu

**Summary of the supplementary information**

| Additional file 1 Fig. S1 | Evaluation of editing efficiency, NHEJ/MMEJ ratio, DNA repair-related gene expression, and knockdown approaches in embryos. |
| --- | --- |
| Additional file 1 Fig. S2 | Integration analysis of *Cre* knock-in at Lypd1 and *Calcr* loci. |
| Additional file 1 Fig. S3 | Two-cell strategy test in mouse and monkey embryos. |
| Additional file 1 Fig. S4 | CATI application in nucleotide replacement of monkey *CDKL5* locus and GOTI experiment for off-target analysis. |
| Additional file 2 Table S1 | An in-depth analysis of the DNA repair patterns associated with 88 sgRNA targets was conducted. |
| Additional file 3 Table S2 | Summary of mice line generated by CATI method, Related to Figure 2 |
| Additional file 4 Table S3 | Primers/Oligos used in this study |
| Additional file 5 Table S4 | Donor DNA used in this study, related to Method |
| Additional file 6 | Review history |


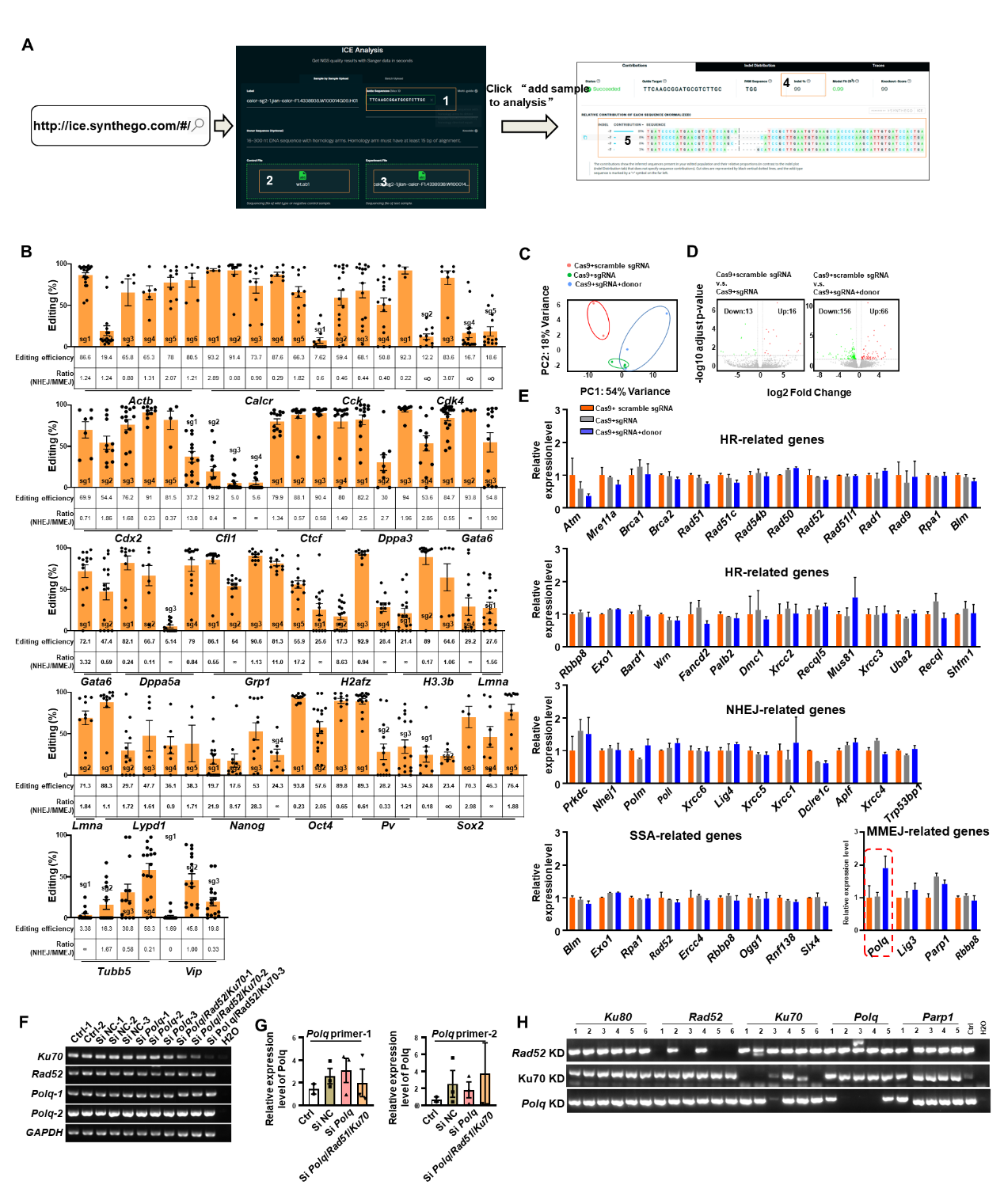


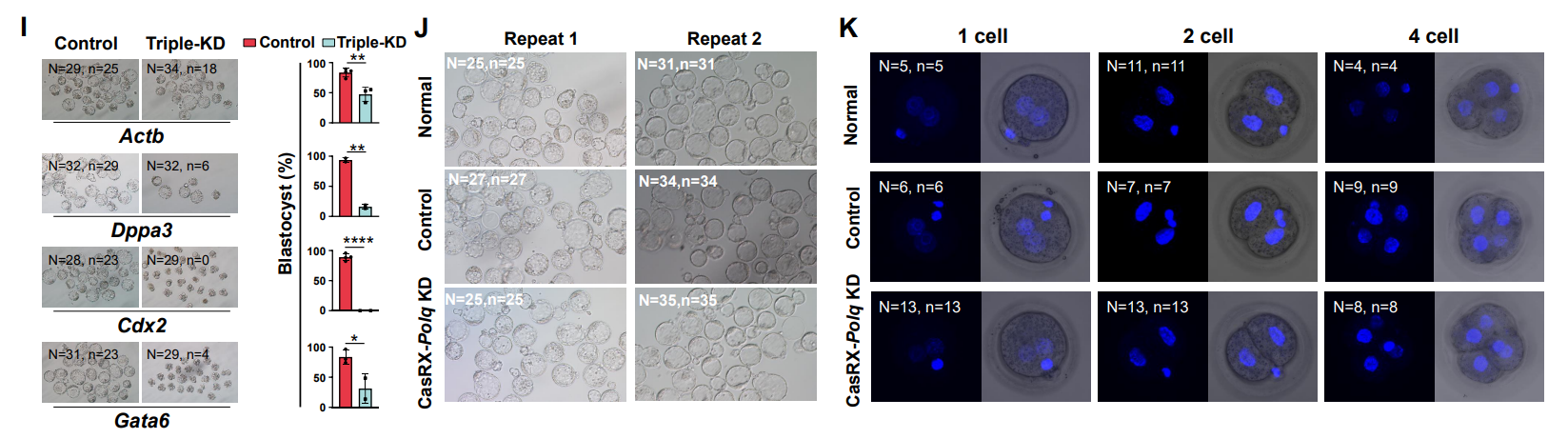


**Figure S1. Evaluation of editing efficiency, NHEJ/MMEJ ratio, DNA repair-related gene expression, and knockdown approaches in embryos.**

1. Workflow diagram for sequence analysis using the ICE website. The number 1 represents the sgRNA sequence without the PAM region, number 2 represents the normal ab1 sequencing file of the target site prior to editing, number 3 represents the ab1 sequencing file after editing, number 4 represents the overall indel ratio at the target site, and number 5 represents the possible sequence types and their respective proportions at the target site after editing.
2. Editing efficiency and NHEJ/MMEJ ratio in mouse embryos are presented in this figure. The orange bars depict the editing efficiency of individual sgRNA, while the corresponding values for both editing efficiency and NHEJ/MMEJ ratio for each sgRNA are provided in the accompanying box below.
3. Principal component analysis of transcriptomes from three group samples. The samples within the same group were circled together. Each dot represents one sample and each sample contains five embryos.
4. The volcano plots illustrate the differentially expressed genes, with up-regulated and down-regulated genes indicated on the plots.
5. The expression of analyzed DNA repair-related genes from three groups. DNA repair pathway includes HR, NHEJ, SSA and MMEJ.
6. RT-PCR result for siRNA strategy in gene suppression.
7. Quantification analysis of siRNA-mediated gene suppression. *Polq* primer-1 and primer-2 indicate two different pairs of primers used in the analysis.
8. RT-PCR analysis of CasRX strategy in suppressing endogenous gene expression in embryos. For each gene, we tested 5 or 6 samples and each sample contain 5 embryos. Sample without specific band indicates the related gene is down-regulated successfully.
9. Embryo development ratio of Control group and Triple-KD group. “N” indicates total number of embryos for micro-injection; “n” indicates number of embryos developing to blastocyst.
10. Imaging showing CasRX-mediated *Polq* knockdown have no obvious influence on embryonic development. “N” indicates total number of embryos for imaging; “n” indicate number of embryos developed to blastocyst.
11. Imaging showing CasRX-mediated *Polq* knockdown have no obvious influence on nuclear morphology at multiple developmental stages. “N” indicates total number of embryos for imaging; “n” indicates number of embryos displaying complete nuclear morphology.





**Figure S2. Integration analysis of *Cre* knock-in at *Lypd1* and *Calcr* loc.**

1. The schematic overview of the strategy to generate *Lypd1*-*Cre, Calcr-Cre* and *Mllt3-loxp-Exon3-loxp* knock-in allele. The sgRNA targeting site is highlighted in cyan followed with a PAM in yellow. The stop codon is highlighted in red. Primers for genotyping and probe for southern blot are marked out.
2. Genotyping analysis for F1 generation of three knock-in lines.
3. Sanger sequencing at knock-in junction sites in 3 different lines.
4. The birth information of F0 mice carrying a gene knock-in is provided.
5. The birth information of F1 mice carrying a gene knock-in is provided.
6. Quantitative analysis was performed for the data presented in (D) and (E).
7. Southern blot analysis was conducted to confirm precise integration at the *Lypd1* and *Calcr* loci, with the desired southern bands highlighted by red rectangles.
8. Detection of Cre protein expression in the hippocampus (Hip), prefrontal cortex (PFC), and cerebellum (Cere) tissues of *Lypd1*-Cre mice was performed using Western blot analysis.


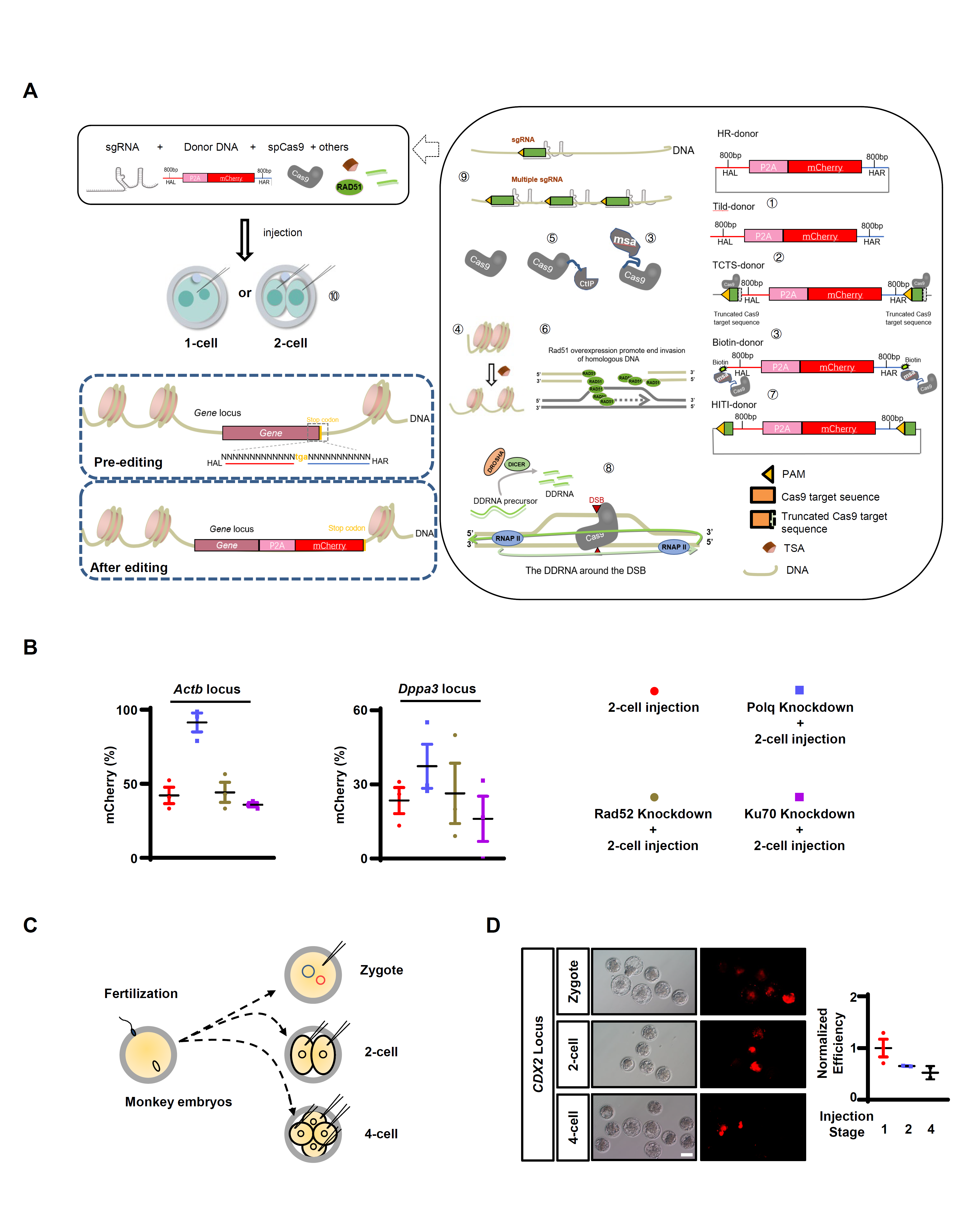


**Figure S3. Two-cell strategy test in mouse and monkey embryos.**

1. The diagram illustrates the injection of a mixture of gene-editing agents into 1-cell or 2-cell embryos to facilitate the integration of a foreign P2A-mCherry DNA fragment into a specific genomic site. Ten different strategies aimed at improving gene knock-in efficiency were presented, which include the “Tild donor”, “TCTS donor”, “Biotin donor”, “TSA treatment”, “Cas9-CtIP fusion”, “HITI donor”, “DDRNA around the DSB”, “Multiple sgRNA”, and “2-cell injection”. The blue dashed box displays the sequence changes of the target site before and after gene editing.
2. HDR efficiency of 2-cell injection strategy together with *Polq, Rad52* or *Ku70* knockdown at *Actb* and *Dppa3* loci.
3. This figure provides an illustration of the gene-editing strategy that involves the injection of zygotes, 2-cell, and 4-cell stage monkey embryos.
4. This figure presents a comparison of knock-in efficiency at the *CDX2* locus using zygote, 2-cell, and 4-cell injection methods. The left panel shows representative images of mCherry-positive embryos, while the right panel presents a quantitative comparison of knock-in efficiency across the different injection stages. Scale bar is 100 μm.


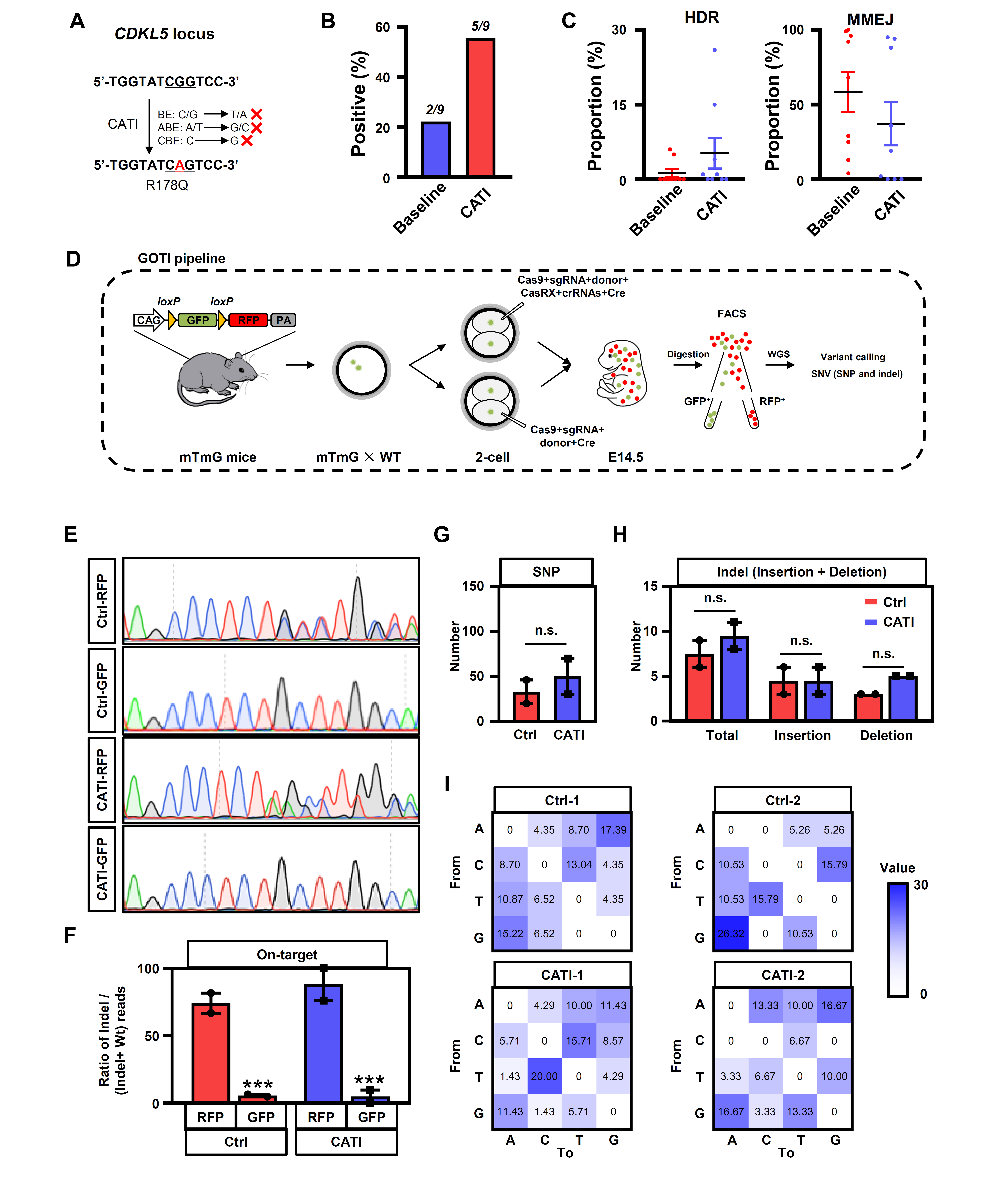


Figure S4-next page


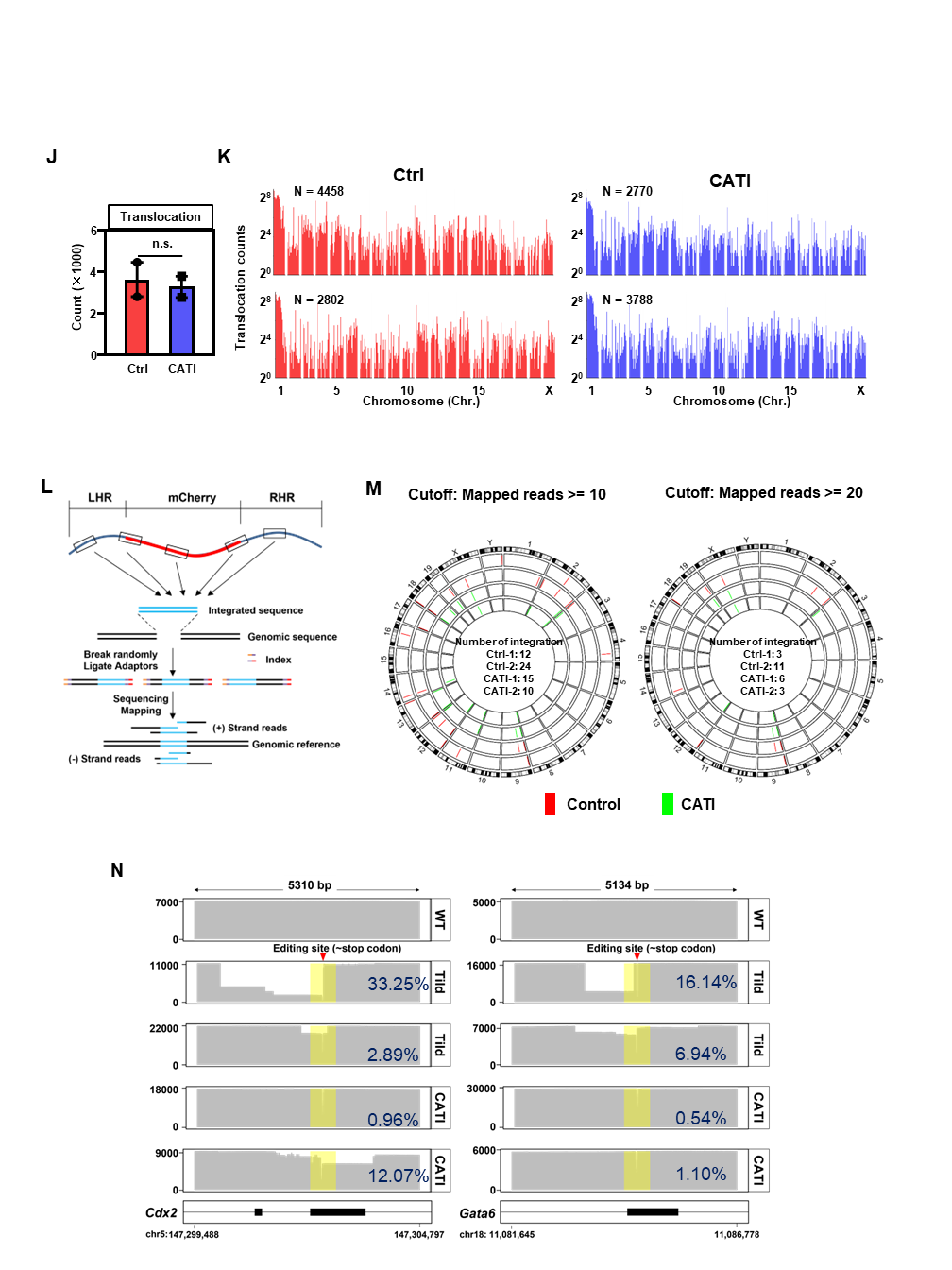


**Figure S4. CATI application in nucleotide replacement of monkey *CDKL5* locus and GOTI experiment for off-target analysis.**

1. This figure illustrates the CDKL5 locus in monkeys, highlighting the nucleotide replacement in red. It should be noted that G-to-A mutation cannot be generated using BE, ABE, or CBE.
2. The rate of successful editing in monkey embryos for R178Q mutations was determined using both the baseline and CATI strategies.
3. Proportion of HDR and MMEJ events of edited monkey embryos at *CDKL5* locus.
4. he workflow for conducting an off-target analysis using the Genome-wide Off-target analysis by Two-cell embryo Injection (GOTI) experiment.
5. The on-target efficiency of RPF-positive and GFP-positive cells was determined for both the control and CATI groups using Sanger sequencing.
6. The On-target efficiency of RPF-positive and GFP-positive cells was evaluated for the control and CATI groups based on whole-genome sequencing (WGS), with ***p < 0.001 considered significant
7. A comparison was made between the total number of detected de novo SNPs in the control and CATI groups, with “n.s.” indicating no significant difference.
8. A comparison was made between the total number of detected *de novo* indels in control and CATI groups, with “n.s.” indicating no significant difference.
9. Distribution of mutation types of control and CATI groups. The number in each cell indicates the proportion of a certain type of mutation among all mutations. Ctrl-1 and Ctrl-2 indicates two independent samples of control group. CATI-1 and CATI-2 indicates two independent samples of CATI group.
10. Comparison of the total number of translocation events in control and CATI groups. n.s. indicates no significant difference.
11. Distribution of total translocation junctions across whole chromosomes (Chr.). Number of counts are listed on the top.
12. Illustration of random integration analysis. Any fragments of donor ligated to reference genome can be recognized as integration counts. After excluding counts mapped to KI site, the remaining counts represent random integration.
13. Location distribution of random integration across mouse genome. Red bars represent control. Green bars represent CATI. Two different results derived from different cutoff have been shown together. The numbers of integration event have been shown in the center of circus.
14. The large deletion analysis of different gene Knock-in strategy on Cdx2 and Gata6 loci. Coverage of PacBio reads at the loci. The loci were PCR-amplified from a pool of embryos (5 embryos per sample), and the resulting products were sequenced using the PacBio platform. The red triangles indicate cut site. WT: unedited embryos, Tild: embryo edited by Tild method, CATI: embryos edited by CATI method. Gene location were shown below. Black rectangles indicate exons.
