# Supplementary material for: CATI: an efficient gene integration method for rodent and primate embryos by MMEJ suppression
Source: Genome Biol. 2023 Jun 23;24:146. doi: 10.1186/s13059-023-02987-w (PMC10288798; doi:10.1186/s13059-023-02987-w)
Supplement: Supplementary file 3 — Additional file 3: Table S2. Summary of mice line generated by CATI method, Related to Fig. 2. [file 13059_2023_2987_MOESM3_ESM.docx]

**Table S2. Summary of mice line generated by CATI method**

| **Line** | **Method** | **Embryos transferred** | **Pups born** | **Positive founders** | **Founder tested** | **Germline transmission** |
| --- | --- | --- | --- | --- | --- | --- |
| ***Calcr-Cre*** | **CATI** | **40** | **16 (40%)** | **4 (25%)** | **#1** | **2/9 (22.22%)** |
|  |  |  |  |  | **#2** | **3/7 (42.86%)** |
|  |  |  |  |  | **#3** | **4/8 (50%)** |
| ***Lypd1-Cre*** | **CATI** | **40** | **18 (45%)** | **5 (27.78%)** | **#1** | **2/6 (33.33%)** |
|  |  |  |  |  | **#2** | **5/13 (38.5%)** |
| ***Mllt3-LoxP*** | **CATI** | **40** | **19 (47.50%)** | **7 (36.84%)** | **#1** | **4/11 (36.36%)** |
|  |  |  |  |  | **#2** | **1/7 (14.29%)** |
