# Supplementary material for: CATI: an efficient gene integration method for rodent and primate embryos by MMEJ suppression
Source: Genome Biol. 2023 Jun 23;24:146. doi: 10.1186/s13059-023-02987-w (PMC10288798; doi:10.1186/s13059-023-02987-w)
Supplement: Supplementary file 4 — Additional file 4: Table S3. Primers/Oligos used in this study. [file 13059_2023_2987_MOESM4_ESM.docx]

**Table S3. Oligos used in this study**

| **Type** | **Name** | **Primer sequence (5'-3')** |
| --- | --- | --- |
| **Primers used for 88 sgRNAs in mouse embryos** | Cas9-sgRNA-R | TTGTGAAAAGCACCGACTCGGTGCCACTTTTTCAAGTTGATAACGGACTAGCCTTATTTTAACTTGCTATTTCTAGCTCTAAAAC |
|  | Actb-sg1-F | GAAATTAATACGACTCACTATAGGgcaccgcaagtgcttctaggGTTTTAGAGCTAGAAATAGC |
|  | Actb-sg2-F | GAAATTAATACGACTCACTATAGGgttaggttttgtcaaagaaaGTTTTAGAGCTAGAAATAGC |
|  | Actb-sg3-F | GAAATTAATACGACTCACTATAGGgaagcacttgcggtgcacgaGTTTTAGAGCTAGAAATAGC |
|  | Actb-sg4-F | GAAATTAATACGACTCACTATAGGgcggtgcacgatggaggggcGTTTTAGAGCTAGAAATAGC |
|  | Actb-sg5-F | GAAATTAATACGACTCACTATAGGaagcaggagtacgatgagtcGTTTTAGAGCTAGAAATAGC |
|  | Actb-sg6-F | GAAATTAATACGACTCACTATAGGtgtccaccttccagcagatgGTTTTAGAGCTAGAAATAGC |
|  | Calcr-sg1-F | GAAATTAATACGACTCACTATAGGgtcttgctggatgacgttcaGTTTTAGAGCTAGAAATAGC |
|  | Calcr-sg2-F | GAAATTAATACGACTCACTATAGGttcaagcggatgcgtcttgcGTTTTAGAGCTAGAAATAGC |
|  | Calcr-sg3-F | GAAATTAATACGACTCACTATAGGggggtggcttcacattcaagGTTTTAGAGCTAGAAATAGC |
|  | Calcr-sg4-F | GAAATTAATACGACTCACTATAGGgtggatcacaatgcttggggGTTTTAGAGCTAGAAATAGC |
|  | Calcr-sg5-F | GAAATTAATACGACTCACTATAGGtccactgagccttcatttccGTTTTAGAGCTAGAAATAGC |
|  | Cck-sg1-F | GAAATTAATACGACTCACTATAGGactacgaatacccatcgtagGTTTTAGAGCTAGAAATAGC |
|  | Cck-sg2-F | GAAATTAATACGACTCACTATAGGatcgtagtgggccagcgtctGTTTTAGAGCTAGAAATAGC |
|  | Cck-sg3-F | GAAATTAATACGACTCACTATAGGcaagacgctggcccactacgaGTTTTAGAGCTAGAAATAGC |
|  | Cck-sg4-F | GAAATTAATACGACTCACTATAGGtccaagcagggccaagacgcGTTTTAGAGCTAGAAATAGC |
|  | Cdk4-sg1-F | GAAATTAATACGACTCACTATAGGaagcgacgcagagtgagaagGTTTTAGAGCTAGAAATAGC |
|  | Cdk4-sg2-F | GAAATTAATACGACTCACTATAGGttctccaccaagactgggaaGTTTTAGAGCTAGAAATAGC |
|  | Cdk4-sg3-F | GAAATTAATACGACTCACTATAGGgcagcactcctacctgcacaGTTTTAGAGCTAGAAATAGC |
|  | Cdk4-sg4-F | GAAATTAATACGACTCACTATAGGgagggtttctccaccaagacGTTTTAGAGCTAGAAATAGC |
|  | Cdk4-sg5-F | GAAATTAATACGACTCACTATAGGtggagaaaccctcgctgaagGTTTTAGAGCTAGAAATAGC |
|  | Cdx2-sg1-F | GAAATTAATACGACTCACTATAGGgggttctggggccagctggaGTTTTAGAGCTAGAAATAGC |
|  | Cdx2-sg2-F | GAAATTAATACGACTCACTATAGGgaggggtcactgggtgacagGTTTTAGAGCTAGAAATAGC |
|  | Cdx2-sg3-F | GAAATTAATACGACTCACTATAGGccacgaacagcatctactgaGTTTTAGAGCTAGAAATAGC |
|  | Cdx2-sg4-F | GAAATTAATACGACTCACTATAGGggcggcggcacagcaatcccGTTTTAGAGCTAGAAATAGC |
|  | Cdx2-sg5-F | GAAATTAATACGACTCACTATAGGagtggaattatggacctcagGTTTTAGAGCTAGAAATAGC |
|  | Cfl1-sg6-F | GAAATTAATACGACTCACTATAGGgggctggaggtggctcacaaGTTTTAGAGCTAGAAATAGC |
|  | Cfl1-sg2-F | GAAATTAATACGACTCACTATAGGtccaggcagggggctggaggGTTTTAGAGCTAGAAATAGC |
|  | Cfl1-sg3-F | GAAATTAATACGACTCACTATAGGgccacctccagccccctgccGTTTTAGAGCTAGAAATAGC |
|  | Cfl1-sg4-F | GAAATTAATACGACTCACTATAGGagatgctccaggcagggggcGTTTTAGAGCTAGAAATAGC |
|  | Ctcf-sg1-F | GAAATTAATACGACTCACTATAGGggtgatgctggggccttgctGTTTTAGAGCTAGAAATAGC |
|  | Ctcf-sg2-F | GAAATTAATACGACTCACTATAGGtgctcggcaccaggactattGTTTTAGAGCTAGAAATAGC |
|  | Ctcf-sg3-F | GAAATTAATACGACTCACTATAGGatcatgctgaggatcatctcGTTTTAGAGCTAGAAATAGC |
|  | Ctcf-sg4-F | GAAATTAATACGACTCACTATAGGgccaccacagacgcccccaaGTTTTAGAGCTAGAAATAGC |
|  | Dppa3-sg1-F | GAAATTAATACGACTCACTATAGGtgcgaaaatcgggaagaattGTTTTAGAGCTAGAAATAGC |
|  | Dppa3-sg2-F | GAAATTAATACGACTCACTATAGGgtgcggcatcgtcgacagccGTTTTAGAGCTAGAAATAGC |
|  | Dppa3-sg3-F | GAAATTAATACGACTCACTATAGGgggacagtgagccattcagaGTTTTAGAGCTAGAAATAGC |
|  | Dppa3-sg4-F | GAAATTAATACGACTCACTATAGGagggatcccatctttgataaGTTTTAGAGCTAGAAATAGC |
|  | Gata6-sg1-F | GAAATTAATACGACTCACTATAGGcctcttggtagcaccagctcGTTTTAGAGCTAGAAATAGC |
|  | Gata6-sg2-F | GAAATTAATACGACTCACTATAGGgctctggccctggcctgagcGTTTTAGAGCTAGAAATAGC |
|  | Gata6-sg3-F | GAAATTAATACGACTCACTATAGGttactgaagtaagaagagatGTTTTAGAGCTAGAAATAGC |
|  | Gata6-sg4-F | GAAATTAATACGACTCACTATAGGggtagcaccagctcaggccaGTTTTAGAGCTAGAAATAGC |
|  | Gata6-sg5-F | GAAATTAATACGACTCACTATAGGgaccggggccttgtctgctaGTTTTAGAGCTAGAAATAGC |
|  | Dppa5-sg1-F | GAAATTAATACGACTCACTATAGGactggcttcactcgatacacGTTTTAGAGCTAGAAATAGC |
|  | Dppa5-sg2-F | GAAATTAATACGACTCACTATAGGgagacacaaggactggaaacGTTTTAGAGCTAGAAATAGC |
|  | Dppa5-sg3-F | GAAATTAATACGACTCACTATAGGcaggtcggagacacaaggacGTTTTAGAGCTAGAAATAGC |
|  | Dppa5-sg4-F | GAAATTAATACGACTCACTATAGGgcatccaggtcggagacacaGTTTTAGAGCTAGAAATAGC |
|  | Grp1-sg1-F | GAAATTAATACGACTCACTATAGGcaggttctcaaggaaaagggGTTTTAGAGCTAGAAATAGC |
|  | Grp1-sg2-F | GAAATTAATACGACTCACTATAGGgggaggaactgccagctgaaGTTTTAGAGCTAGAAATAGC |
|  | Grp1-sg3-F | GAAATTAATACGACTCACTATAGGcttgtcgttgtcccttcagcGTTTTAGAGCTAGAAATAGC |
|  | Grp1-sg4-F | GAAATTAATACGACTCACTATAGGagctgaagggacaacgacaaGTTTTAGAGCTAGAAATAGC |
|  | Grp1-sg5-F | GAAATTAATACGACTCACTATAGGaacgacaagggcggcttccaGTTTTAGAGCTAGAAATAGC |
|  | H2afz-sg1-F | GAAATTAATACGACTCACTATAGGggacaacagaagactgtttaGTTTTAGAGCTAGAAATAGC |
|  | H2afz-sg2-F | GAAATTAATACGACTCACTATAGGgaagactgtttaaggatgccGTTTTAGAGCTAGAAATAGC |
|  | H2afz-sg3-F | GAAATTAATACGACTCACTATAGGcctgagataataaggaatccGTTTTAGAGCTAGAAATAGC |
|  | H2afz-sg4-F | GAAATTAATACGACTCACTATAGGtttagagtcctgagataataGTTTTAGAGCTAGAAATAGC |
|  | H3.3b-sg1-F | GAAATTAATACGACTCACTATAGGaagctctctccccccgtatcGTTTTAGAGCTAGAAATAGC |
|  | H3.3b-sg2-F | GAAATTAATACGACTCACTATAGGagttggctcgccggatacggGTTTTAGAGCTAGAAATAGC |
|  | H3.3b-sg3-F | GAAATTAATACGACTCACTATAGGcatgcccaaagacatccagtGTTTTAGAGCTAGAAATAGC |
|  | H3.3b-sg4-F | GAAATTAATACGACTCACTATAGGgtaaattctgtaaaatacttGTTTTAGAGCTAGAAATAGC |
|  | LMNA-sg1-F | GAAATTAATACGACTCACTATAGGaactgcagcatcatgtaatcGTTTTAGAGCTAGAAATAGC |
|  | LMNA-sg2-F | GAAATTAATACGACTCACTATAGGaatctgggacctgccaggcaGTTTTAGAGCTAGAAATAGC |
|  | Lypd1-sg1-F | GAAATTAATACGACTCACTATAGGcttccacttagccctctgctGTTTTAGAGCTAGAAATAGC |
|  | Lypd1-sg2-F | GAAATTAATACGACTCACTATAGGttcagcagtgtgccaagcagGTTTTAGAGCTAGAAATAGC |
|  | Lypd1-sg3-F | GAAATTAATACGACTCACTATAGGcccctgctgcctcacctgtcGTTTTAGAGCTAGAAATAGC |
|  | Lypd1-sg4-F | GAAATTAATACGACTCACTATAGGacaggtgaggcagcaggggtGTTTTAGAGCTAGAAATAGC |
|  | Lypd1-sg5-F | GAAATTAATACGACTCACTATAGGtggcacactgctgaagctaaGTTTTAGAGCTAGAAATAGC |
|  | Nanog-sg1-F | GAAATTAATACGACTCACTATAGGagatcacaagaaagagtgcgGTTTTAGAGCTAGAAATAGC |
|  | Nanog-sg2-F | GAAATTAATACGACTCACTATAGGtatgagacttacgcaacatcGTTTTAGAGCTAGAAATAGC |
|  | Nanog-sg3-F | GAAATTAATACGACTCACTATAGGcgtaagtctcatatttcaccGTTTTAGAGCTAGAAATAGC |
|  | Nanog-sg4-F | GAAATTAATACGACTCACTATAGGcttaaagtcagggcaaagccGTTTTAGAGCTAGAAATAGC |
|  | Oct4-sg2-F | GAAATTAATACGACTCACTATAGGctctgttcccgtcactgctcGTTTTAGAGCTAGAAATAGC |
|  | Oct4-sg3-F | GAAATTAATACGACTCACTATAGGggtgcctcagtttgaatgcaGTTTTAGAGCTAGAAATAGC |
|  | Oct4-sg4-F | GAAATTAATACGACTCACTATAGGgacaagagaacctggagcttGTTTTAGAGCTAGAAATAGC |
|  | Oct4-sg1-F | GAAATTAATACGACTCACTATAGGggttggagcccaacctatag GTTTTAGAGCTAGAAATAGC |
|  | Pv-sg1-F | GAAATTAATACGACTCACTATAGGtctggtggctgaaagctaagGTTTTAGAGCTAGAAATAGC |
|  | Pv-sg3-F | GAAATTAATACGACTCACTATAGGcgttggggatggagaggtggGTTTTAGAGCTAGAAATAGC |
|  | Pv-sg3-F | GAAATTAATACGACTCACTATAGGttagctttcagccaccagagGTTTTAGAGCTAGAAATAGC |
|  | Sox2-sg1-F | GAAATTAATACGACTCACTATAGGggtacgttaggcgcttcgcaGTTTTAGAGCTAGAAATAGC |
|  | Sox2-sg2-F | GAAATTAATACGACTCACTATAGGtaatggccgtgccgggcacc GTTTTAGAGCTAGAAATAGC |
|  | Sox2-sg3-F | GAAATTAATACGACTCACTATAGGccagcactaccagagcggccGTTTTAGAGCTAGAAATAGC |
|  | Sox2-sg4-F | GAAATTAATACGACTCACTATAGGcaggggcagtgtgccgttaaGTTTTAGAGCTAGAAATAGC |
|  | Sox2-sg5-F | GAAATTAATACGACTCACTATAGGccgcagcgaaacgacagctgGTTTTAGAGCTAGAAATAGC |
|  | Tubb5-sg1-F | GAAATTAATACGACTCACTATAGGgaggcagaagaggaggcctaGTTTTAGAGCTAGAAATAGC |
|  | Tubb5-sg2-F | GAAATTAATACGACTCACTATAGGgatgcagggctctctgccttGTTTTAGAGCTAGAAATAGC |
|  | Tubb5-sg3-F | GAAATTAATACGACTCACTATAGGtttcggagaggaggcagaagGTTTTAGAGCTAGAAATAGC |
|  | Tubb5-sg4-F | GAAATTAATACGACTCACTATAGGcggagaggaggcagaagaggGTTTTAGAGCTAGAAATAGC |
|  | Vip-sg1-F | GAAATTAATACGACTCACTATAGGttgaagagctggagaaatgaGTTTTAGAGCTAGAAATAGC |
|  | Vip-sg2-F | GAAATTAATACGACTCACTATAGGgctggagaaatgatgggaagGTTTTAGAGCTAGAAATAGC |
|  | Vip-sg3-F | GAAATTAATACGACTCACTATAGGctgatttcagctctgcccagGTTTTAGAGCTAGAAATAGC |
| **Type** | **Name** | **siRNA sequence (5'-3')** |
| **Oligos used for gene silence in mouse embryo** | si K70 #1 | cactgtgccttactctgtgaata |
|  | si K70 #2 | aggaactgctagatgctcttatc |
|  | si K70 #3 | acgtctccccgtattttgtggct |
|  | si Rad52 #1 | agaggtggcagccaagcatgcgg |
|  | si Rad52 #2 | acagcgtcccacatatccattgc |
|  | si Rad52 #3 | tgatgtggatttaactaaaacaa |
|  | si polq #1 | tggcgcttgctttaaagggaatg |
|  | si polq #2 | cgtggctgttagaaaatgagttc |
|  | si polq #3 | gctgcttcctacattgactcttt |
| **Type** | **Name** | **Primer sequence (5'-3')** |
| **Oligos used for gene knock-down by CasRX system** | CasRX-sgRNA-F | TAATACGACTCACTATAGGAACCCCTACCAACTGGTCGGGGTTTGAAAC |
|  | mouse-K70-sg1-R | cactgtgccttactctgtgaataGTTTCAAACCCCGACCAGTT |
|  | mouse-K70-sg2-R | aggaactgctagatgctcttatcGTTTCAAACCCCGACCAGTT |
|  | mouse-K70-sg3-R | acgtctccccgtattttgtggctGTTTCAAACCCCGACCAGTT |
|  | mouse-K80-sg1-R | acttgcggcaatacatgttttccGTTTCAAACCCCGACCAGTT |
|  | mouse-K80-sg2-R | ctgtgcgtctttaagaagattgaGTTTCAAACCCCGACCAGTT |
|  | mouse-K80-sg3-R | ctgagcgctattgatgatctgatGTTTCAAACCCCGACCAGTT |
|  | mouse-Parp1-sg1-R | ccgattggcttaatactgctgggGTTTCAAACCCCGACCAGTT |
|  | mouse-Parp1-sg2-R | atggtgtccaaaagtgcaaactaGTTTCAAACCCCGACCAGTT |
|  | mouse-Parp1-sg3-R | gcatgcttcacatatcagcaagtGTTTCAAACCCCGACCAGTT |
|  | mouse-Rad52-sg1-R | agaggtggcagccaagcatgcggGTTTCAAACCCCGACCAGTT |
|  | mouse-Rad52-sg2-R | acagcgtcccacatatccattgcGTTTCAAACCCCGACCAGTT |
|  | mouse-Rad52-sg3-R | tgatgtggatttaactaaaacaaGTTTCAAACCCCGACCAGTT |
|  | mouse-Polq-sg1-R | tggcgcttgctttaaagggaatgGTTTCAAACCCCGACCAGTT |
|  | mouse-Polq-sg2-R | cgtggctgttagaaaatgagttcGTTTCAAACCCCGACCAGTT |
|  | mouse-Polq-sg3-R | gctgcttcctacattgactctttGTTTCAAACCCCGACCAGTT |
|  | monkey-POLQ-sg1-R | gtggaggtgattctgaaaagtgcGTTTCAAACCCCGACCAGTT |
|  | monkey-POLQ-sg2-R | atgctgcctgcacatttttggctGTTTCAAACCCCGACCAGTT |
|  | monkey-POLQ-sg3-R | tgtggctgctagaaaatgaattcGTTTCAAACCCCGACCAGTT |
|  | monkey-POLQ-sg4-R | tggtggtcgacctttagatattcGTTTCAAACCCCGACCAGTT |
| **Type** | **Name** | **Primer sequence (5'-3')** |
| **Oligos used for the indentification of sgRNA editing efficiency in mouse and monkey embryo** | Actb-F | agccttccttcttgggtaa |
|  | Actb-R | caactgctgtcgccttca |
|  | Calcr-F | tagttagtgctcctcgggct |
|  | Calcr-R | aacaccttgcctgctttcct |
|  | Cck-F | tctcctgtctccaccttt |
|  | Cck-R | ccatttctgaagccatcta |
|  | Cdk4-F | gaaacagccatgttgggtgg |
|  | Cdk4-R | aaggagaggtggggacttgt |
|  | Cdx2-F | tctaggttaaaatttggtttc |
|  | Cdx2-R | aaatctaaaccatgtcttccc |
|  | Cfl1-F | cagatagaactccaccccacc |
|  | Cfl1-R | ttagaagttggcagcatggga |
|  | Ctcf-F | acagccatcattcaggtcgaa |
|  | Ctcf-R | tctcagtatgtttggtaaaat |
|  | Dppa3-F | aaaattctcagcccccaggaa |
|  | Dppa3-R | tctggatcgttgtgcatccta |
|  | Gata6-F | gcacaccccattcagcatagc |
|  | Gata6-R | ataggtttccatatgatatcc |
|  | Dppa5-F | cactgcttgcatccctctga |
|  | Dppa5-R | ctcacaagtcccacttcccc |
|  | Grp-F | agggctcctcaccagagttt |
|  | Grp-R | tggtagcaaattggagccct |
|  | H2afZ-F | ggtggtggtatgtcatccctaaa |
|  | H2afZ-R | aaatttggttggttggaaggct |
|  | H3.3b(sg1)-F | tcgcctcggtctcagcaa |
|  | H3.3b(sg1)-R | ttcccagccctctgcctac |
|  | H3.3b(sg2/3)-F | gagatccgtcgttaccag |
|  | H3.3b(sg2/3)-R | cagtcactcttcccattca |
|  | LMNA-F | tagtcacccgctcctacctc |
|  | LMNA-R | tgagggtaaagccaaggcag |
|  | Lypd1-F | agcagcctgtctcattgctt |
|  | Lypd1-R | gagtcaccacagactggctc |
|  | Nanog(sg1)-F | tgtggctgacctatggctgtt |
|  | Nanog(sg1)-R | tggccgttccaggactga |
|  | Nanog(sg2/3/4)-F | ggctatctggtgaacgcatct |
|  | Nanog(sg2/3/4)-R | cgccctcttctggagtgtct |
|  | Oct4-F | ttgagtattcccaacgaga |
|  | Oct4-R | caatggctatgaggtgatg |
|  | Pv-F | ccattccctcatccacag |
|  | Pv-R | acttgccaaaccaacacc |
|  | Sox2-F | gatcagcatgtacctccccg |
|  | Sox2-R | cccagcaagaaccctttcct |
|  | Tubb5-F | agtttacggctatgttccgc |
|  | Tubb5-R | cagaaatctaagtttgtcagagtg |
|  | Vip-F | gcagggaccattgttgat |
|  | Vip-R | gaagggtaagtagaccagaagt |
|  | Mllt3-F | ctgttgttctcatcttcc |
|  | Mllt3-R | aatggcaatcactcttca |
|  | Pnoc-F | gctgcagaaaaggtttgggg |
|  | Pnoc-R | atgccagtccacagtagtgc |
|  | Sod1(G93A)-F | acctttgctaactcaggagc |
|  | Sod1(G93A)-R | tgcatctcccgagggtaactt |
|  | Sod1(A4V)-F | aatagcgactttccccgctc |
|  | Sod1(A4V)-R | agccccagaaggataacgga |
|  | CDX2-F | tttccctggcatcttcac |
|  | CDX2-R | atcccacttgtcttactccc |
|  | H3.3B-F | gcgctgcaggtaagacaaagg |
|  | H3.3B-R | atcacccatcccttctgcatatt |
|  | CDKL5-F | tgggatgacttcgaaaacacg |
|  | CDKL5-R | agccagaatgtcaaactgtgt |
| **Type** | **Name** | **Primer sequence (5'-3')** |
| **Oligos used for SSODN** | CDKL5 | a*g*aaggcaataatgctaattacacagagtacgttgccaccagCtggtatcAgtcccTGgagcCTttacttgggtgagttaccatcccaaaatagaatgac*a* |
|  | Ctcf | cagcatgatggaccggtgatgctggggccttgctcggcaccaggactattGAATTCgggctgtgtttaaacggcccaaatcttaatttttctcttttttttctttg |
|  | Oct4 | gggatgctgtgagccaaggcaagggaggtagacaagagaacctggagcttGAATTCtggggttaaattcttttactgaggagggattaaaagcacaacaggggtgg |
|  | Sod1(A4V) | ccctccggaggaggccgccgcgcgtctcccggggaagcatggcgatgaaagtagtgtgcgtgctgaaaggcgacggtccggtgcagggaaccatccacttcga |
|  | Sod1(G93A) | ttaatgttaggcatgttggagacctgggcaatgtgactgctggaaaggacgccgtggcaaatgtgtccattgaagatcgtgtgatctcactctcaggagagca |
| **Type** | **Name** | **Primer sequence (5'-3')** |
| **Oligos used for Linearized donor amplification (mouse)** | Tild- Actb-F | ccgggacctgacagactacct |
|  | Tild- Actb-R | agccctcccactagataccat |
|  | Biotin-Tild- Actb-F | ccgggacctgacagactacct (with 5'-Biotin modification) |
|  | Biotin-Tild- Actb-R | agccctcccactagataccat (with 5'-Biotin modification) |
|  | TCSC-Tild- Actb-F | TGGCGGGACTAGTGGCTCATCGCCTAGAAGCACTTGCGGccgggacctgacagactacct |
|  | TCSC-Tild- Actb-R | TGGCGGGACTAGTGGCTCATCGCCTAGAAGCACTTGCGGagccctcccactagataccat |
|  | HITI-Tild- Actb-F | gcctagaagcacttgcggaagcttggcgtaatcatggtca |
|  | HITI-Tild- Actb-R | caagtgcttctaggcggactTTACTTGTACAGCTCGTCCAtgcc |
|  | Tild- Dppa3-F | gtaaaccaggctggccttaaa |
|  | Tild- Dppa3-R | tgaaaggggttgaggcaaaca |
|  | Biotin-Tild- Dppa3-F | gtaaaccaggctggccttaaa (with 5'-Biotin modification) |
|  | Biotin-Tild- Dppa3-R | Tgaaaggggttgaggcaaaca (with 5'-Biotin modification) |
|  | TCSC-Tild- Dppa3-F | TCCGCCCTAGAGTCCTAACTaaaatcgggaagaattagggatcttcctgcccctgctttc |
|  | TCSC-Tild- Dppa3-R | TCCGCCCTAGAGTCCTAACTaaaatcgggaagaattaggtaacctgccaattagtgcttg |
|  | Tild- H3.3b-F | gtttccggccgcccgtgga |
|  | Tild- H3.3b-R | ttagtaatagatgatgctggt |
|  | Tild- LMNA-F | gacagaggtcaccttcctgc |
|  | Tild- LMNA-R | gaaaaagcaagcacggcaga |
|  | Tild- Gata6-F | cagaaccccagcacacccc |
|  | Tild- Gata6-R | ggaacacagaagtgggctgt |
|  | HITI-Tild- Gata6-F | agcaccagctcaggccagggaagcttggcgtaatcatggtca |
|  | HITI-Tild- Gata6-R | gcctgagctggtgctaccTTACTTGTACAGCTCGTCCAtgcc |
|  | Tild -Cfl1-F | gggggtgccagggttgtaa |
|  | Tild- Cfl1-R | aacagtgcaccccaactcaa |
|  | Tild- Cdk4-F | ataccgtggcctggggaactg |
|  | Tild- Cdk4-R | actggtggttggacttatgtc |
|  | Tild- Cdx2-F | acactcttgcagaggatcgga |
|  | Tild- Cdx2-R | tcccgacttcccttcaccata |
|  | Tild- H2afZ-F | ggcgagagaggaggaaagtg |
|  | Tild- H2afZ-R | tcggtggaggtactttcgttg |
|  | Tild- Tubb5-F | gtaccctcaagctcaccacg |
|  | Tild- Tubb5-R | gagaaccaatacagggaagacact |
|  | Tild- Dppa5-F | acggctctcaaaacaacaaga |
|  | Tild- Dppa5-R | agtgaggtggatggaggtca |
|  | Tild- -Calcr-F | aaggcagcaggtaagttttct |
|  | Tild- Calcr-R | gacattgtatggatggtaaca |
|  | Tild- Lypd1-F | tgaggggagactttgatttta |
|  | Tild- Lypd1-R | tcagagaccaaaccagctggg |
|  | Tild- Mllt3-F | gcgtgggtttcaggtctatc |
|  | Tild- Mllt3-R | acacagactccttctgacgac |
|  | Tild- Pnoc-F | atctgctggtatcagagatct |
|  | Tild- Pnoc-R | ataaagtctctgaggaaaggt |
| **Oligos used for Linearized donor amplification (monkey)** | Tild- CDX2-F | gccaggggtgttagtttaatc |
|  | Tild- CDX2-R | tcatcagccccaagattgcga |
|  | Tild- H3.3B-F | gtgattcagaggtcccgacg |
|  | Tild- H3.3B-R | atcccccagttagtgtttgcat |
| **Type** | **Name** | **Primer sequence (5'-3')** |
| **Oligos used for large deletion detection** | Cdx2-F | CACGGACCATCTTTGCGTTC |
|  | Cdx2-R | TCCAGCTGGCTTTACCGATG |
|  | Gata6-F | GGCTCTCACTCACACTGCTT |
|  | Gata6-R | CAGTGCCCGGATGATCACTT |
| **Type** | **Name** | **Primer sequence (5'-3')** |
| **Oligos used for DDRNA sequence(mouse)** | Actb-DDRNA1 | tctaggcggactgttactgag |
|  | Actb-DDRNA2 | tttgacaaaacctaacttgcg |
|  | Actb-DDRNA3 | tcatcgtactcctgcttgctg |
|  | Actb-DDRNA4 | tctgctggaaggtggacagtg |
|  | H3.3b-DDRNA1 | taaattctgtaaaatactttg |
|  | H3.3b-DDRNA2 | agcggtttttatggcattttg |
|  | H3.3b-DDRNA3 | tgtctttgggcatgatggtga |
|  | H3.3b-DDRNA4 | ttggcgtggatggcacacaga |
