# Supplementary material for: CATI: an efficient gene integration method for rodent and primate embryos by MMEJ suppression
Source: Genome Biol. 2023 Jun 23;24:146. doi: 10.1186/s13059-023-02987-w (PMC10288798; doi:10.1186/s13059-023-02987-w)
Supplement: Supplementary file 5 — Additional file 5: Table S4. Donor DNA used in this study, related to Method. [file 13059_2023_2987_MOESM5_ESM.docx]

**Table S4. Donor DNA used in this study**

| yellow:20bp sgRNA sequence; green:PAM sequence; red:stop; gray:homologous arm; pink:P2A sequence; red:mCherry sequence; cyan:Cre sequence |
| --- |
| **1, Mouse-Actb-mCherry-donor** |
| **Actb site (before insertion)**  ……tcattgctcctcctgagcgcaagtactctgtgtggatcggtggctccatcctggcctcactgtccaccttccagcagatgtggatcagcaagcaggagtacgatgagtccggcccctccatcgtgcaccgcaagtgcttctaggcggactgttactgagctgcgttttacaccctttctttgacaaaacctaacttgcgcagaaaaaaaaaaaataagagacaacattggcatggctttgtttttttaaattttttttaaagtttttttttttttttttttttttttttttaagtttttttgttttgttttggcgcttttgactcaggat……  **Actb site (after insertion)**  ……gacctgacagactacctcatgaagatcctgaccgagcgtggctacagcttcaccaccacagctgagagggaaatcgtgcgtgacatcaaagagaagctgtgctatgttgctctagacttcgagcaggagatggccactgccgcatcctcttcctccctggagaagagctatgagctgcctgacggccaggtcatcactattggcaacgagcggttccgatgccctgaggctcttttccagccttccttcttgggtaagttgtagcctagtcctttctccatctaaaggtgacaaaactcctgaggccatagtacaagttaagtctgatttctgtcactcttctcttaggtatggaatcctgtggcatccatgaaactacattcaattccatcatgaagtgtgacgttgacatccgtaaagacctctatgccaacacagtgctgtctggtggtaccaccatgtacccaggcattgctgacaggatgcagaaggagattactgctctggctcctagcaccatgaagatcaaggtaagctaagcatccttagcttggtgagggtgggccctgtggttgtcagagcaacTttctaggtttaaggggaatcccagcacccagagagctcaccattcaccatcttgtcttgctttcttcagatcattgctcctcctgagcgcaagtactctgtgtggatcggtggctccatcctggcctcactgtccaccttccagcagatgtggatcagcaagcaggagtacgatgagtccggcccctccatcgtgcaccgcaagtgcttcGCCACGAACTTCTCTCTGTTAAAGCAAGCAGGAGACGTGGAAGAAAACCCCGGTCCTatggtgagcaagggcgaggaggataacatggccatcatcaaggagttcatgcgcttcaaggtgcacatggagggctccgtgaacggccacgagttcgagatcgagggcgagggcgagggccgcccctacgagggcacccagaccgccaagctgaaggtgaccaagggtggccccctgcccttcgcctgggacatcctgtcccctcagttcatgtacggctccaaggcctacgtgaagcaccccgccgacatccccgactacttgaagctgtccttccccgagggcttcaagtgggagcgcgtgatgaacttcgaggacggcggcgtggtgaccgtgacccaggactcctccctgcaggacggcgagttcatctacaaggtgaagctgcgcggcaccaacttcccctccgacggccccgtaatgcagaagaagaccatgggctgggaggcctcctccgagcggatgtaccccgaggacggcgccctgaagggcgagatcaagcagaggctgaagctgaaggacggcggccactacgacgctgaggtcaagaccacctacaaggccaagaagcccgtgcagctgcccggcgcctacaacgtcaacatcaagttggacatcacctcccacaacgaggactacaccatcgtggaacagtacgaacgcgccgagggccgccactccaccggcggcatggacgagctgtacaagtgagcggactgttactgagctgcgttttacacTctttctttgacaaaacctaacttgcgcagaaaaaaaaaaaataagagacaacattggcatggctttgtttttttaaattttttttaaagtttttttttttttttttttttttttttttaagtttttttgttttgttttggcgcttttgactcaggatttaaaaactggaacggtgaaggcgacagcagttggttggagcaaacatcccccaaagttctacaaatgtggctgaggactttgtacattgttttgtttttttttttttttggttttgtctttttttaatagtcattccaagtatccatgaaataagtggttacaggaagtccctcaccctcccaaaagccacccccactcctaagaggaggatggtcgcgtccatgccctgagtccaccccggggaaggtgacagcattgcttctgtgtaaattatgtactgcaaaaatttttttaaatcttccgccttaatacttcatttttgtttttaatttctgaatggcccaggtctgaggcctcccttttttttgtccccccaacttgatgtatgaaggctttggtctccctgggagggggttgaggtgttgaggcagccagggctggcctgtacactgacttgagaccaataaaagtgcacaccttaccttacacaaacagcttgtggctctgtggctttgctgggtgtggggagcaggttgggtgggtgtggagctctattggggggggcatctagggtgggctaggccttgctgatggtatctagtg…… |
| **2, Mouse-Gata6-mCherry-donor** |
| **Gata6 site (before insertion)**  ……cctcaagtattcaggtcaagacggcctctacataggtgtcagtctgtcctcccctgccgaagtcacatcctccgtgcgacaggattcttggtgtgctctggccctggcctgagctggtgctaccaagaggcaaggagggctctgaaggcctcataccacttgtgtctgatattgtccagcagtccagatggcagcaaaaatgcagacataacattccttcgatgcgtgatttctgtgcctttgttttgaaagagatatatttctcaagaagcttactgaagtaagaagagatgggcttttgcaggaagggccagcaccgtgggcatgtgg……  **Gata6 site (after insertion)**  ……caggtcctgcttggggaaaggagtccctgggacacacagaaccccagcacaccccattcagcatagctgagtctggtgccttggggttcttcaaaataatggccagagcatcaaaatggatgtttgaggaatttaacagagtgatgtttccaagcattatttgaaaaattttaaggaatcataagctgtttcaaacaaatacccggtatggaatggagccctggaggctggggagaacttagggtgcttgagtcttgcgaggaaaaaggtatgattagagaatgaacagctgcggctgaagtttcgcggaagccttgcaagtgtgactggtcccttaaacgctaagcagatgccagttacttgggtttggtcctacagcgtttgcagtgagaaccctgacagcgtggtttgtttctgctttccactcttaagtttgacaaattggagtcggtctttcggaaccctttcctgtctacctctgctttgtgctgggtggtcactgggttccgaaggtaacaacactgttctaagaatgttgccagaaccaggaggatggcactggatgacagtggtcagcacagtgtgtgccccttgggcgaggaagagtaaggctggctttagttttgctgactatgtcatttgatccctgatgttgaggcaggaggattggaccagctaacaacatgatgtataaccatctctccccctctgttctcttcttccatcaggtaggggcatcagtgatgtctgcagtgggagaaaacgccaaccccgagaacagtgacctcaagtattcaggtcaagacggcctctacataggtgtcagtctgtcctcccctgccgaagtcacatcctccgtgcgacaggattcttggtgtgctctggccctggccGCCACGAACTTCTCTCTGTTAAAGCAAGCAGGAGACGTGGAAGAAAACCCCGGTCCTatggtgagcaagggcgaggaggataacatggccatcatcaaggagttcatgcgcttcaaggtgcacatggagggctccgtgaacggccacgagttcgagatcgagggcgagggcgagggccgcccctacgagggcacccagaccgccaagctgaaggtgaccaagggtggccccctgcccttcgcctgggacatcctgtcccctcagttcatgtacggctccaaggcctacgtgaagcaccccgccgacatccccgactacttgaagctgtccttccccgagggcttcaagtgggagcgcgtgatgaacttcgaggacggcggcgtggtgaccgtgacccaggactcctccctgcaggacggcgagttcatctacaaggtgaagctgcgcggcaccaacttcccctccgacggccccgtaatgcagaagaagaccatgggctgggaggcctcctccgagcggatgtaccccgaggacggcgccctgaagggcgagatcaagcagaggctgaagctgaaggacggcggccactacgacgctgaggtcaagaccacctacaaggccaagaagcccgtgcagctgcccggcgcctacaacgtcaacatcaagttggacatcacctcccacaacgaggactacaccatcgtggaacagtacgaacgcgccgagggccgccactccaccggcggcatggacgagctgtacaagtgagctggtgctaccaagaggcaaggagggctctgaaggcctcataccacttgtgtctgatattgtccagcagtccagatggcagcaaaaatgcagacataacattccttcgatgcgtgatttctgtgcctttgttttgaaagagatatatttctcaagaagcttactgaagtaagaagagatgggcttttgcaggaagggccagcaccgtgggcatgtggcctgctcctgccagcctgggctgcttcctgcctctgactctgccccataccagtgggagaaactgtgacaatgaccggggccttgtctgctaaggaagattgagagatttaagagaaaatgtttgtgtattgctccaaatcatgtgcttcttgtgatcaacctcggttatcccagaacccattcatccccgaccaccgtgcacatttcacaagcgttcgtggagaggagcactgggagccatttggtctatcctggaggcggagtgcattcctggggtctcaacaagaatattaatttgcaagattgcatcatgacagacactgactgacttatctcaacgttcatcgtaacgtggctgatctgaggtcacttggaatttgtaaacagggtagcaaacaagatatttttcttccatgtacacaataatttttttaagtgcaatttgcgttgcagcaatcagtgttaaatcatttgcataagatttaacagcatttttataatgaatgtaaacattttaacttaatggtacttaaaataatttaaaaaaaagttaactttagacatatgcttcttacactcacagcccacttctgtgttcccaattgtttaaaagaaaaaaaaaaagatttcaagaacaaatcttctctcaggaaattgccttttctccatttatgaatttttatacaagaacaccaacacagtccccgttctt…… |
| **3, Mouse-H3.3-mCherry-donor** |
| **H3.3 site (before insertion)**  ……acctggtggggttgtttgaagataccaatctgtgtgccatccacgccaagagagtcaccatcatgcccaaagacatccagttggctcgccggatacggggggagagagcttaagttgaagcggtttttatggcattttgtagtaaattctgtaaaatactttggtttaatttgtgactttttttgtaagaaattgtttataatatgttgcatttgtacttaagtcattccatctttcactcaggatgaatgggaagagtgactgactgttcacagacctcagtgatgtgagcactgtggctcaggagtgacaagttgctaatacgcagaa……  **H3.3 site (after insertion)**  ……gtttccggccgcccgtggagcgggaagttgctcctccccgcgctccccctcccgggggaggccgcagcccggcgcagcctgagtcattaggggagggggaggaggcggcggccgccatctgctgcggcgaggaggggcgcctggcagccggagcccggtgacctggccttgaacgtcgcttgtctcgcaTgtgaaaaaaaatggcccgaaccaagcagaccgctaggaagtccaccggtgggaaagccccccgcaaacagctggccaccaaggcggctcggaaaagcgcgccctctaccggcggggtgaagaagcctcaccgctacaggtaggcagagggctgggaacaatgacttggccgccggcttgcgggcgggcgctctctcccttctccctgaccgttgacgccttccttcttctggtgactgcaggccagggaccgtggctctgagagagatccgtcgttaccagaaatcgactgagctgctcatccggaagctgccattccagagattggtgagggagatcgcccaggatttcaaaaccgacttgaggtttcaaagtgcagccatcggtgcccttcaggtaaagggacaaaaggcttggtggcactcggggcggcggcttgattgctccagtaggagtgttaatgctgttgctcttgtcctcaacaggaggctagcgaagcatacctggtggggttgtttgaagataccaatctgtgtgccatccacgccaagagagtcaccatcatgcccaaagacatccagttggctcgccggatacggggAgagagagctGCCACGAACTTCTCTCTGTTAAAGCAAGCAGGAGACGTGGAAGAAAACCCCGGTCCTatggtgagcaagggcgaggaggataacatggccatcatcaaggagttcatgcgcttcaaggtgcacatggagggctccgtgaacggccacgagttcgagatcgagggcgagggcgagggccgcccctacgagggcacccagaccgccaagctgaaggtgaccaagggtggccccctgcccttcgcctgggacatcctgtcccctcagttcatgtacggctccaaggcctacgtgaagcaccccgccgacatccccgactacttgaagctgtccttccccgagggcttcaagtgggagcgcgtgatgaacttcgaggacggcggcgtggtgaccgtgacccaggactcctccctgcaggacggcgagttcatctacaaggtgaagctgcgcggcaccaacttcccctccgacggccccgtaatgcagaagaagaccatgggctgggaggcctcctccgagcggatgtaccccgaggacggcgccctgaagggcgagatcaagcagaggctgaagctgaaggacggcggccactacgacgctgaggtcaagaccacctacaaggccaagaagcccgtgcagctgcccggcgcctacaacgtcaacatcaagttggacatcacctcccacaacgaggactacaccatcgtggaacagtacgaacgcgccgagggccgccactccaccggcggcatggacgagctgtacaagtgagttgaagcggtttttatggcattttgtagtaaattctgtaaaatactttggtttaatttgtgactttttttgtaagaaattgtttataatatgttgcatttgtacttaagtcattccatctttcactcaggatgaatgggaagagtgactgactgttcacagacctcagtgatgtgagcactgtggctcaggagtgacaagttgctaatacgcagaagggatgggtgatacttcttgcttttcatgatgcatgtttctgtatgttaatgacttgttgggtagctattaaggtactagaattgataaatgtgtacagggtccttttgcaataaaactggttatgacttgatccaagtgtttaaccatacatcactgtgatagaatgtgggctttttcaaaggttgaagatacaagttttagccacagtgtaacagtttcctttaaaaaaaaaaaagtaaacctggcagctatagaatacactatgtgcatttataatagctattttatatattgtagtgttcaacatttttaaattaaatgttttacattcacaagtggtggggagtcttgtcattaaggtgtgtgtaatttagtccagttggtatttcctgactagactgcatttgtttttaacagtagaaaaatgctatgcgtattaaaccttgcataagtcctcattctaccacatgttcactaacccctgaccctctggctggtaacacaacactaacggggattttatttataagggctctagagtaaaataacaagctattcacaccagcatcatctattactaa…… |
| **4, Mouse-Dppa3-mCherry-donor** |
| **Dppa3 site (before insertion)**  ……taaaacaatagataaatgggatttgtttcattgggtttacaatatcctgattaacctattttgttatttatttcagtttgaacgggacagtgagccattTagatgCctctgcacAttctgTcattatcaaagatgggatccctctgagaatgcgaaaatcgggaagaattaggagcttacattgtacgctgccctggctgtcgacgatgccgcacagcagatgtgaaagctattttttgtttaagattaaactttttctggtgctgggaaatcttaacttgttaacctttaaattgtagataggatgcacaacgatccagatttatgtga……  **Dppa3 site (after insertion)**  tttattatgagtacactgtagctgtcttcagacacaccagaagagggcgtcagatctcgttacggatggttgtgagccaccatgtggttgctgggatttgaactcaggacctttggaagagcagtcagtgctcttaacctctaagccatcactccagtcccaataaatactcttaatgcatgggtgggttgggtgtctgtttcattgtttgatttagactaggaatgtgtctggggagtatgtggaaaaaattctcagcccccaggaagtctggttattgaagcagtaattaaaacaatagataaatgggatttgtttcattgggtttacaatatcctgattaacctattttgttatttatttcagtttgaacgggacagtgagccattTagatgCctctgcacAttctgTcattatcaaagatgggatccctctgagaatgcgaaaatcgggaagaatGCCACGAACTTCTCTCTGTTAAAGCAAGCAGGAGACGTGGAAGAAAACCCCGGTCCTatggtgagcaagggcgaggaggataacatggccatcatcaaggagttcatgcgcttcaaggtgcacatggagggctccgtgaacggccacgagttcgagatcgagggcgagggcgagggccgcccctacgagggcacccagaccgccaagctgaaggtgaccaagggtggccccctgcccttcgcctgggacatcctgtcccctcagttcatgtacggctccaaggcctacgtgaagcaccccgccgacatccccgactacttgaagctgtccttccccgagggcttcaagtgggagcgcgtgatgaacttcgaggacggcggcgtggtgaccgtgacccaggactcctccctgcaggacggcgagttcatctacaaggtgaagctgcgcggcaccaacttcccctccgacggccccgtaatgcagaagaagaccatgggctgggaggcctcctccgagcggatgtaccccgaggacggcgccctgaagggcgagatcaagcagaggctgaagctgaaggacggcggccactacgacgctgaggtcaagaccacctacaaggccaagaagcccgtgcagctgcccggcgcctacaacgtcaacatcaagttggacatcacctcccacaacgaggactacaccatcgtggaacagtacgaacgcgccgagggccgccactccaccggcggcatggacgagctgtacaagtgagagcttacattgtacgctgccctggctgtcgacgatgccgcacagcagatgtgaaagctattttttgtttaagattaaactttttctggtgctgggaaatcttaacttgttaacctttaaattgtagataggatgcacaacgatccagatttatgtgaagtttagaagcctcaagctgtgaggcccagggctgaggaataaagtaaatagaatttggagtatgtacgttctaatttccagaaatttgtaataaaagcatttttgttagctcgactctttgtaatttacacaaacagctaggggctactgtaa |
| **5, Mouse-LMNA-mCherry-donor** |
| **LMNA site (before insertion)**  ……tgtctgaaccccagactcgaggtcagggcaaggcccagagtgtgagggttggggagacaaccccctttggggtcagggagggagaggaagggccagccactgctgctcacacctctgccttctcttctctcttagagctcccagaactgcagcatcatgtaatctgggacctgccaggcagggctgggggcagaggccacctgctcccccctcaccacatgccacctcctgtctgctccttaggagagcaggcctgaagccaaagaaaaatttatcccctgcctttggttttttttttttttcttctattttttttttctttttctaaga……  **LMNA site (after insertion)**  tttcccccatcatgcccttcctcccagccacaggtctcccaagtccccatcacttggttgtctgggtacagacagaggtcaccttcctgcccaatggccaggaagctccaagagcccacagcctaggtgccggtcctaagaagtcagtcccaaactcgctgtccctcctgagccttgtctcccttcccagggttcccactgcagcggctcgggggaccccgctgagtacaacctgcgctcacgcaccgtgctgtgcgggacgtgtgggcagcctgctgacaaggctgccggtggagcgggagcccaggtgggcggatccatctcctctggctcttctgcctccagtgtcacagtcactcgaagcttccgcagtgtggggggcagtgggggtggcagcttcggggacaacctagtcacccgctcctacctcctgggcaactccagtccccggagccaggtgagtcatctctgccctacagcaggacactgctcactgagcagcagggcagggcagcccaagggagtggggtccccctccttgcagtccctcttgcatcctgcccctcctgtctgaaccccagactcgaggtcagggcaaggcccagagtgtgagggttggggagacaaccccctttggggtcagggagggagaggaagggccagccactgctgctcacacctctgccttctcttctctcttagagctcccagaactgcagcatcatgGCCACGAACTTCTCTCTGTTAAAGCAAGCAGGAGACGTGGAAGAAAACCCCGGTCCTatggtgagcaagggcgaggaggataacatggccatcatcaaggagttcatgcgcttcaaggtgcacatggagggctccgtgaacggccacgagttcgagatcgagggcgagggcgagggccgcccctacgagggcacccagaccgccaagctgaaggtgaccaagggtggccccctgcccttcgcctgggacatcctgtcccctcagttcatgtacggctccaaggcctacgtgaagcaccccgccgacatccccgactacttgaagctgtccttccccgagggcttcaagtgggagcgcgtgatgaacttcgaggacggcggcgtggtgaccgtgacccaggactcctccctgcaggacggcgagttcatctacaaggtgaagctgcgcggcaccaacttcccctccgacggccccgtaatgcagaagaagaccatgggctgggaggcctcctccgagcggatgtaccccgaggacggcgccctgaagggcgagatcaagcagaggctgaagctgaaggacggcggccactacgacgctgaggtcaagaccacctacaaggccaagaagcccgtgcagctgcccggcgcctacaacgtcaacatcaagttggacatcacctcccacaacgaggactacaccatcgtggaacagtacgaacgcgccgagggccgccactccaccggcggcatggacgagctgtacaagtgatctgggacctgccaAgcagAgctgggggcagaggccacctgctcccccctcaccacatgccacctcctgtctgctccttaggagagcaggcctgaagccaaagaaaaatttatcccctgcctttggttttttttttttttcttctattttttttttctttttctaagagaagttattttctacagtggttttatactgaaggaaaaactcaagcaaaaaaaaaaaaaatctttatctcaatcctaagtccttcccctttctttccttgtatctgccttaaaaccaaagggcttctctaggagcccagggaaaggactgctttttatagagtctagatttttgtcctgctgccttggctttaccctcatcccaggaccctgtgacaatggtgcctgagaggcaggcatggagttctcttcaccagcctcctccaacagctggcccactgccacgccagctgcagagaaatggggcgcagagaggatgactgagaaggtcaagcccctccccggcactacacgaggccgaggctcctctgcctgccttaccttcttcctgcccttccctagcctggggcgagtggattcccagaggcaaatctgccgtgcttgctttttctatattttatttagacaagagatgggaatgacgggga |
| **6, Mouse-Dppa5a-mCherry-donor** |
| **Dppa5a site (before insertion)**  ……agaggtccacaaaaacatgcttaaagagctggtgaatcatgcggccttcctcctgttccatgacacttaatcctgtttggtgtttctcacgcaggagtgctgaagctggaggaatccatgaagaccctggagctaggccagtgtatcgagtgaagccagtttccagtccttgtgtctccgacctggatgcaggttaagctgtggccagtgtttggttctggcgggatttttagctttgttacatcctagcaagatattcctggatccctgctgcgcatt……  **Dppa5a site (after insertion)**  tcgaggtcttcatttacggctctcaaaacaacaagattcgggctaaatggatgcttcagtccatggctgagaggtaccacctgcgccagcaaaaaggtgagagtctctaacaggtgccctgaaccaaggtgcggtggagtagcctcctgcctccaccgagtttacctcccttttcacatctccactgcttgcatccctctgaggatgccttacttttgggcatcacctgggactcctccttggtctctaggccgtctctgacaaccttttgggagttcgggggcccagacgtaagtcttaaggtgagaaggggtagggacttatccctctgggcagggttatctgaggtaaccctgccccaccccactcccatctccaggtttctaggagaacgttagaactggcccagtggagccctggttctaattagaggtccacaaaaacatgcttaaagagctggtgaatcatgcggccttcctcctgttccatgacacttaatcctgtttggtgtttctcacgcaggagtgctgaagctggaggaatccatgaagaccctggagctaggTcagtgtatcgagGCCACGAACTTCTCTCTGTTAAAGCAAGCAGGAGACGTGGAAGAAAACCCCGGTCCTatggtgagcaagggcgaggaggataacatggccatcatcaaggagttcatgcgcttcaaggtgcacatggagggctccgtgaacggccacgagttcgagatcgagggcgagggcgagggccgcccctacgagggcacccagaccgccaagctgaaggtgaccaagggtggccccctgcccttcgcctgggacatcctgtcccctcagttcatgtacggctccaaggcctacgtgaagcaccccgccgacatccccgactacttgaagctgtccttccccgagggcttcaagtgggagcgcgtgatgaacttcgaggacggcggcgtggtgaccgtgacccaggactcctccctgcaggacggcgagttcatctacaaggtgaagctgcgcggcaccaacttcccctccgacggccccgtaatgcagaagaagaccatgggctgggaggcctcctccgagcggatgtaccccgaggacggcgccctgaagggcgagatcaagcagaggctgaagctgaaggacggcggccactacgacgctgaggtcaagaccacctacaaggccaagaagcccgtgcagctgcccggcgcctacaacgtcaacatcaagttggacatcacctcccacaacgaggactacaccatcgtggaacagtacgaacgcgccgagggccgccactccaccggcggcatggacgagctgtacaagtgaagcAagtttcAagtcAttgtgtctccgacctggatgcaggttaagctgtggccagtgtttggttctggcgggatttttagctttgttacatcctagcaagatattcctggatccctgctgcgcattctgatgtgaatcccaaggttaccactctaaataaaaaataaaattgaagtgattaaaatgtgcgtcgtcattaaaatatgaaaagagccagtacccttttcccggtgtccgtttgaatccttgtaaatgcctgaacagattatagcctgtggaatcttaagcttaggcatccaccctgttgtgaaacactgtctagtttgtaacatggaatgtgaattgctgccttaggggaagtgggacttgtgagggttgaagagccaccaaagtaggttttcagtttaccctaattgagaccagtttgatagttaacgtggaataaatcccatgtggggagctttctggctttatgacctaaggcaaattgtttaatccctgttcctggtccatcttcttgggaggcaggaggatatctggagttcatgaccagtcttaactacaaagcaagttctaggccagtttgggctaagtgggaccctatctccaaacaaagctttgtatgtgattctatacatttaacacctacgtgtaggagacttaagaacaagtacgtgtagcaagtgctattgacagcagtgggagaaaaccctcgtctgtggagagaagtctgtgtttccagcgcataactgctctcccttttgtgagcagtaaggaaaccgcgatactgtggtctagctaaggctgacctccatccacctcactatcctctccccaccacttcc |
| **7, Mouse-Cfl1-mCherry-donor** |
| **Cfl1 site (before insertion)**  ……ctccttcccagatagaactccaccccaccccagtgcttcctgctcactgctctcttttcaccctgtaggaatcaagcatgaattacaagctaactgctacgaggaggtcaaggaccgctgcaccctggcagagaaactaggtggcagcgccgtcatttccctggagggcaagcctttgtgagccacctccagccccctgcctggagcatcttaacagccccagacctgctcttgggtgttgcaggctgcccctttcctgccagaccggaggggctggggggatcccagcagggggagggctatcccttcaccccagttgccaaacatccc……  **Cfl1 site (after insertion)**  ttctgcctgcctctggttccctttctcagagcttaacggaaattattccaggtgtggggggtgccagggttgtaacaacccttccagtgaggagctaggtgtggtggtacccacacctttagtcccagcttgagggaggcaaaggcaggcagatgcctgtgagtttgagcctaacctggtctacagaaagaccctgtgtttcaaaaacaaaaactagtgaggaacttggggaaaagaaaatccctgctgttagagtatcctaaaagtttcttttcttgagtcattattccctggatgtagctgcagccttttctttgctttgtctttagggcccccgagaatgcacccctcaagagcaaaatgatctatgccagctccaaggatgccatcaagaagaagctgacaggtaaggatccctccttctgcaaaagtcatgggcgcagtctcctgctagctgctaccttcctcctctcatctgctccgccggctccttcccagatagaactccaccccaccccagtgcttcctgctcactgctctcttttcaccctgtaggaatcaagcatgaattacaagctaactgctacgaggaggtcaaggaccgctgcaccctggcagagaaactaggtggcagcgccgtcatttccctggagggcaagcctttgGCCACGAACTTCTCTCTGTTAAAGCAAGCAGGAGACGTGGAAGAAAACCCCGGTCCTatggtgagcaagggcgaggaggataacatggccatcatcaaggagttcatgcgcttcaaggtgcacatggagggctccgtgaacggccacgagttcgagatcgagggcgagggcgagggccgcccctacgagggcacccagaccgccaagctgaaggtgaccaagggtggccccctgcccttcgcctgggacatcctgtcccctcagttcatgtacggctccaaggcctacgtgaagcaccccgccgacatccccgactacttgaagctgtccttccccgagggcttcaagtgggagcgcgtgatgaacttcgaggacggcggcgtggtgaccgtgacccaggactcctccctgcaggacggcgagttcatctacaaggtgaagctgcgcggcaccaacttcccctccgacggccccgtaatgcagaagaagaccatgggctgggaggcctcctccgagcggatgtaccccgaggacggcgccctgaagggcgagatcaagcagaggctgaagctgaaggacggcggccactacgacgctgaggtcaagaccacctacaaggccaagaagcccgtgcagctgcccggcgcctacaacgtcaacatcaagttggacatcacctcccacaacgaggactacaccatcgtggaacagtacgaacgcgccgagggccgccactccaccggcggcatggacgagctgtacaagtgagcTacctcTagccccctgcctAgagcatcttaacagccccagacctgctcttgggtgttgcaggctgcccctttcctgccagaccggaggggctggggggatcccagcagggggagggctatcccttcaccccagttgccaaacatccctcccaccccctggaccgtccttctccctccatccctgacggttctggccttcccaaactgcttttgatcttctgattcctcttgggttgacgcagaccaagtcccgtcctaggcacccagtttggggggagcctgtatttttttttttaacgacacccctactccgtatccctccccatcccatgctgccaacttctaaccacaatagtgactctgtgcttgtctgtttagttctgtgtgtaaatgaaatgtggaaatgaccctccctgccccagctggctgccctcccctttcctttgatcttgaccactcatggaagcaggaccagtaagggaccttcaatttaaaacaaaacaaaacaaaaaaacaataaaaaggctaattaacaacatggcttttgggtttgggattttggggaccctgtggtgggagatggatgggtggtttccctgtgcttcacctctagttcagtccctagtaagcggcatctccttcttgagttggggtgcactgttccaaggcagggcagttgggctccgagtgtgtcacacctgaaacttcttacctttatagtggacagtgaggtaccctgagcccctttccacatgtgggctaaatacagtttacttgggtgacactgctttctaaggttctcttcagctctcccctctcttcc |
| **8, Mouse-Cdx2-mCherry-donor** |
| **Cdx2 site (before insertion)**  ……cagcctcagccgggtgccctgcggagcgtgcccgagcccttgagtcctgtgacctccttgcaaggctcagtgcctggttctgtccctggggttctggggccagctggaggggttttaaactccactgtcacccagtgacccctcccgtggtctgaagcggcggcggcacagcaatcccaggctgagccatgaggagtatggacgctgcgagaatcctcagaagagattcctctcctcctacccacgaacagcatctactgatggagattgaggacagaagatgagtggaattatggacctcaggggaagacatggtttagattttttttt……  **Cdx2 site (after insertion)**  acactcttgcagaggatcggagttctgtttccagcatccacgtcaggtggctcacagtgtcacagtgtcggctaactctgattgcaggggacacccagtgcctctggcttctgtggatatatatgaatgtatacagacatacacatatcaaagatttaaaaatccttgaactttttttttcattaacacaaaagaggccctacttgcacaccaacactttctgtagtctagggaaaagggaaggaacaaacaagagaaattcatagccaggaagttttggacaaagtttcattgtctgtgccaaggtgaagaacatcgcagtaccaatgagctgtgggagcccaagggtgacccacgaggcacactgagctggccaaccatttgtgtagggaatagctttcgtcatggttccgttccctggttctgaggttctgttgctagtaaagggttttttgtttgtttgttttttgttttttgaaagcaacttggggagggggcaggtaagtaaagaggacgaggctggcctggctttgggaacatttcccaaactcagtgaaagaaggacgctcccggggtggcgccatttctctgttcttatcttcttctaccttctattttgtcatctctaggttaaaatttggtttcagaaccgcagagccaaggagaggaaaatcaagaagaagcagcagcagcaacagcagcagcagcaacaacagcctccacagccgccgccacaaccttcccagcctcagccgggtgccctgcggagcgtgcccgagcccttgagtcctgtgacctccttgcaaggctcagtgcctggttctgtccctggggttctggggccagctggaggggttttaaactccactgtcacccagGCCACGAACTTCTCTCTGTTAAAGCAAGCAGGAGACGTGGAAGAAAACCCCGGTCCTatggtgagcaagggcgaggaggataacatggccatcatcaaggagttcatgcgcttcaaggtgcacatggagggctccgtgaacggccacgagttcgagatcgagggcgagggcgagggccgcccctacgagggcacccagaccgccaagctgaaggtgaccaagggtggccccctgcccttcgcctgggacatcctgtcccctcagttcatgtacggctccaaggcctacgtgaagcaccccgccgacatccccgactacttgaagctgtccttccccgagggcttcaagtgggagcgcgtgatgaacttcgaggacggcggcgtggtgaccgtgacccaggactcctccctgcaggacggcgagttcatctacaaggtgaagctgcgcggcaccaacttcccctccgacggccccgtaatgcagaagaagaccatgggctgggaggcctcctccgagcggatgtaccccgaggacggcgccctgaagggcgagatcaagcagaggctgaagctgaaggacggcggccactacgacgctgaggtcaagaccacctacaaggccaagaagcccgtgcagctgcccggcgcctacaacgtcaacatcaagttggacatcacctcccacaacgaggactacaccatcgtggaacagtacgaacgcgccgagggccgccactccaccggcggcatggacgagctgtacaagtgacccctcccgtggtctgaagcggcggcggcacagcaatcccaTgctgagccatgaggagtatggacgctgcgagaatcctcagaagagattcctctcctcctacccacgaacagcatctactgatggagattgaggacagaagatgagtggaattatggacctcaggggaagacatggtttagattttttttttctttttaacttttcccattccgactcttcctgccagcaacgacaaacgaagtgattcctggggcttcttcgttcatgctctttgccaggactgactaccgacatgaagctatcagcctcttttgccccagctctttgcctctctgtatttctgtgtggagctgaggagagagtgagactggatggggtgggggtagcaatacttgagccaaggtggctgtttcctgctgactgctttctgagaaccagctggccgtcctgcctccgggccagggactattcaaactacaggagccagaggcagctaagatagctggactgaccgaagtctgcagaacctcccccaccaggtggtctgggctttcttctccacaaatcaggaaggggtggtgggttcaggggctgcggtgagagggggttggttagccaacgccaggcccctgcgacaagggcttgtttagaaagcctgtcaccagagctgctgtaggcggaatgtatgtctgtgttgtaaatgccagagccaacctggacttcctgtcccttccctcgtctttggctgaagaagaccggaattgtttgctgctgttcgagtcactgatctgtgtaacgagccaaacaagccttttaaaaagccttcttgatccatgggtagagaagttgtatggtgaagggaagtcggga |
| **9, Mouse-H2afZ-mCherry-donor** |
| **H2afZ site (before insertion)**  ……ttcactttgtatatgaattttacctgctttgagttcatgtattgtttttttaaatgtttatccagaaggtagacttaatctgtgtccctctcctaggtgtcatcccacacatccacaaatcgctgatcgggaagaaaggacaacagaagactgtttaaggatgcctggattccttattatctcaggactctaaatattcctaacagctgtccagtgttggtgattccagtggactgtatctctgtgaaaaacacaattttgcctttttgtaattctatttgagcaagttggaggcttaattagccttccaaccaaccaaatttctgcatt……  **H2afZ site (after insertion)**  ggagtacgcctgtgatctggaaaagctctgggggggcgggggaaagtggcgagagaggaggaaagtgatgcaagaaaactcgcaggccctaagagcggcgcgcagaccgagggagcgctagagggagctcgcgttcagccgaggaggctgctgggccagagtgtgctgctcctgcaaatgcaattgcgtgccccttcggggttttttgggtggttttttttttttttttgcataattctgcataattttatcattcaggtagtagggaatgacaacagttggtaaataacaccattgaggttcaaagatcttgattgaaagtaagttggaatattgatcatcagatttttatttattttttgccttttaggtacttgagttggcaggaaatgcgtcaaaagacttaaaggtaaagcgtatcacccctcgtcacttgcagcttgctatacgtggagatgaagaattggattctctgatcaaagctaccattgctggtggtggtatgtcatccctaaactcctaacattctatttaagaagaaaattcctacactcccattgtttctagaagagatttcagtacagggtgctttcatggggtttggctttccacttagtttttgctaagatcattgttcatccctcaagttttcactttgtatatgaattttacctgctttgagttcatgtattgtttttttaaatgtttatccagaaggtagacttaatctgtgtccctctcctaggtgtcatcccacacatccacaaatcgctgatcgggaagaaaggacaacagaagactgttGCCACGAACTTCTCTCTGTTAAAGCAAGCAGGAGACGTGGAAGAAAACCCCGGTCCTatggtgagcaagggcgaggaggataacatggccatcatcaaggagttcatgcgcttcaaggtgcacatggagggctccgtgaacggccacgagttcgagatcgagggcgagggcgagggccgcccctacgagggcacccagaccgccaagctgaaggtgaccaagggtggccccctgcccttcgcctgggacatcctgtcccctcagttcatgtacggctccaaggcctacgtgaagcaccccgccgacatccccgactacttgaagctgtccttccccgagggcttcaagtgggagcgcgtgatgaacttcgaggacggcggcgtggtgaccgtgacccaggactcctccctgcaggacggcgagttcatctacaaggtgaagctgcgcggcaccaacttcccctccgacggccccgtaatgcagaagaagaccatgggctgggaggcctcctccgagcggatgtaccccgaggacggcgccctgaagggcgagatcaagcagaggctgaagctgaaggacggcggccactacgacgctgaggtcaagaccacctacaaggccaagaagcccgtgcagctgcccggcgcctacaacgtcaacatcaagttggacatcacctcccacaacgaggactacaccatcgtggaacagtacgaacgcgccgagggccgccactccaccggcggcatggacgagctgtacaagtgaggatgcTtggattcTttattatctcaggactctaaatattcctaacagctgtccagtgttggtgattccagtggactgtatctctgtgaaaaacacaattttgcctttttgtaattctatttgagcaagttggaggcttaattagccttccaaccaaccaaatttctgcattcgagtcttaaccatatttaagtgttactgtggcttcaaagaagctattgattctgaagtagtgggttttgattgagttgactgtttttaaaaaactgtttggattttaattgtgatgcagaagttatagtaacaagcatttggttttgtacagacattgtttccactctggtggataagctcaataaaggtcatatcccaaactagctttaaacttgcttaataatcgggacttaccttagatctcactcagcaacaagtacattctctgcttactaattaaacagtgcatctgtagtcataaccccgtggtacttttgttgtttctttgtatcatatgacctcttagggtcttaaaacttgggaagaagctggaggtaactgcttttggcgtcccccccccccccaattgacagttaactaatgtgaaagggcctgtatcctaaggtgggacacaagtacagagaccctcagggtacaacgaaagtacctccaccgaatgaacttcgga |
| **10, Mouse-Tubb5-mCherry-donor** |
| **Tubb5 site (before insertion)**  ……cgggtgagggcatggacgagatggagttcaccgaagctgagagcaacatgaacgacctcgtctctgagtaccagcagtaccaggatgccaccgcggaagaggaagaggatttcggagaggaggcagaagaggaggcctaaggcagagagccctgcatcagctcaggctgcttagatccctcagctttctccaactgccctttgtcctccagtttctttctgctgcctctgtcttgtatttgttttgttctgttttctcattgggggtaaatggtgcctggcacatggcaggcactcaataaatatttgtttgtggaatgtctcctttctc……  **Tubb5 site (after insertion)**  acaatgccaccctgtctgtccatcagttggttgagaacacggatgagacctactgcatcgacaacgaggccctctacgacatctgcttccgtaccctcaagctcaccacgccaacctacggagacctgaaccatctcgtctcggccaccatgagcggcgtcaccacctgcctccgtttcccgggccagcttaatgctgaccttcgaaagctggctgtcaacatggtgccattcccacgtctccacttcttcatgcctggctttgcccctctcaccagccgtggaagccagcagtaccgggccctcactgtgcctgaacttacccagcaggtcttcgatgccaagaacatgatggccgcctgcgacccgcgccacggccggtacctcacagttgccgccgtcttccgtggacggatgtccatgaaggaggtggatgagcagatgctcaacgtgcagaacaagaatagcagctacttcgtggaatggatccccaacaatgtcaagacagctgtctgtgacatcccaccgcgtggcctcaagatggcagtcaccttcattggaaacagcacagccatccaggagctcttcaagcgcatctctgagcagtttacggctatgttccgccggaaggctttcctccactggtatacgggtgagggcatggacgagatggagttcaccgaagctgagagcaacatgaacgacctcgtctctgagtaccagcagtaccaggatgccaccgcggaagaggaagaggatttcggagaggaggcagaagaAgaAgccGCCACGAACTTCTCTCTGTTAAAGCAAGCAGGAGACGTGGAAGAAAACCCCGGTCCTatggtgagcaagggcgaggaggataacatggccatcatcaaggagttcatgcgcttcaaggtgcacatggagggctccgtgaacggccacgagttcgagatcgagggcgagggcgagggccgcccctacgagggcacccagaccgccaagctgaaggtgaccaagggtggccccctgcccttcgcctgggacatcctgtcccctcagttcatgtacggctccaaggcctacgtgaagcaccccgccgacatccccgactacttgaagctgtccttccccgagggcttcaagtgggagcgcgtgatgaacttcgaggacggcggcgtggtgaccgtgacccaggactcctccctgcaggacggcgagttcatctacaaggtgaagctgcgcggcaccaacttcccctccgacggccccgtaatgcagaagaagaccatgggctgggaggcctcctccgagcggatgtaccccgaggacggcgccctgaagggcgagatcaagcagaggctgaagctgaaggacggcggccactacgacgctgaggtcaagaccacctacaaggccaagaagcccgtgcagctgcccggcgcctacaacgtcaacatcaagttggacatcacctcccacaacgaggactacaccatcgtggaacagtacgaacgcgccgagggccgccactccaccggcggcatggacgagctgtacaagtgaggcgcgccggcagagagccctgcatcagctcaggctgcttagatccctcagctttctccaactgccctttgtcctccagtttctttctgctgcctctgtcttgtatttgttttgttctgttttctcattgggggtaaatggtgcctggcacatggcaggcactcaataaatatttgtttgtggaatgtctcctttctctttccactctgacaaacttagatttctgacattctggttgttaccctgtgcttcctactggtatcttttttttttttttttttttttttttggtttttccagacagggtttctctgtatatatagcccaggctgtcctggaactcagaaatccgcctgcctctgcctcccaagtgttgggattaaaggcgtgtgccactatcacccaacaagtatccatttttttaaattattatttgactgtccagttaatattccagaatattctccagaaacctgaggtctgctccagatcctgtgtagaacccagcccaattctaagaactcggatagtaaccatcatcttaaagcaaagtagtggatcagggatgctaggtagagaccaccaggaagagaagggggtggggttttccagtcagggccatttagaatccacctatgctttcagtcagcagggctttgttttgtttttctcctgcctcatctctcagcctcaggagaggtattaacagtattatctccatttatatcctcccagctgtcctgagccaaatctgccattggaagtgtcttccctgtattggttctcctttctcggagagatgggggttgggggtgcggcaaggtcttggtcttggtctctgaacactcccaattcccgc |
| **11, Mouse-Cdk4-mCherry-donor** |
| **Cdk4 site (before insertion)**  ……ggccagccagagtgggacaaaggaaacagccatgttgggtgggcatcagcatcggctcctgagctgttttctgacctttgtcctctccctttaggaaatgctgacctttaacccacataagcgaatctctgccttccgagccctgcagcactcctacctgcacaaggaggaaagcgacgcagagtgagaagaggggctgcctttcccagtcttggtggagaaaccctcgctgaagcGgcagcctctgtttccccccaaggctgtggagaatcctccagttttttacagagaatattttaagccttaaataacaagtccccacctctcctt……  **Cdk4 site (after insertion)**  ggttgctgggaattgactcaggagctctggaatagtggtcagtgctcttagctgctgagccatctctccagtcctgtagtgccactcggggcgagatggacagatccctggtaatacagcgctgacagagtctccaatgagatggaaggcaaggactggcagcccagactcttgacctctgcatgtatacacgcacatgtgcgtgcccgcatttcacacatgagcacgtgcacgcagagatgaaacagacgggcccagaccagcctgaagctaagatgtagctgtgggcaggctgtagttgtcactgctgttgggaatggccagccagagtgggacaaaggaaacagccatgttgggtgggcatcagcatcggctcctgagctgttttctgacctttgtcctctccctttaggaaatgctgacctttaacccacataagcgaatctctgccttccgagccctgcagcactcctacctgcacaaagaggaaagcgacgcagagGCCACGAACTTCTCTCTGTTAAAGCAAGCAGGAGACGTGGAAGAAAACCCCGGTCCTatggtgagcaagggcgaggaggataacatggccatcatcaaggagttcatgcgcttcaaggtgcacatggagggctccgtgaacggccacgagttcgagatcgagggcgagggcgagggccgcccctacgagggcacccagaccgccaagctgaaggtgaccaagggtggccccctgcccttcgcctgggacatcctgtcccctcagttcatgtacggctccaaggcctacgtgaagcaccccgccgacatccccgactacttgaagctgtccttccccgagggcttcaagtgggagcgcgtgatgaacttcgaggacggcggcgtggtgaccgtgacccaggactcctccctgcaggacggcgagttcatctacaaggtgaagctgcgcggcaccaacttcccctccgacggccccgtaatgcagaagaagaccatgggctgggaggcctcctccgagcggatgtaccccgaggacggcgccctgaagggcgagatcaagcagaggctgaagctgaaggacggcggccactacgacgctgaggtcaagaccacctacaaggccaagaagcccgtgcagctgcccggcgcctacaacgtcaacatcaagttggacatcacctcccacaacgaggactacaccatcgtggaacagtacgaacgcgccgagggccgccactccaccggcggcatggacgagctgtacaagtgagaagaggggctgcctttcccagtcttggtggagaaaccctcgctgaagcTgcagcctctgtttccccccaaggctgtggagaatcctccagttttttacagagaatattttaagccttaaataacaagtccccacctctccttacgaggttcacccccattaccctcccctagctctacactaaagggcaggtgtatctgtcttcttccctccctgatttatactgggatcttttttatacaggaaaacaagacaagacaaagagtatggtctttatttttctttaatgttttttccaattggctttgccactgggagacttggggaaa |
| **12, Monkey-CDX2-mCherry-donor** |
| **CDX2 site (before insertion)**  ……tgcagcagcaacagcagcagcagccgccgcagccgcctccgccgccaccacagcctccccagcctcagccaggtcctctgagaagtgtccaggagcccttgagtccggtgtcttccctgcaagcctcagtgcctggctctgtccctggggttctggggccaactgggggggtgctaaaccccactgtcacccagtgacccacaggagtctgcagcagcagagcaattccaggctgagccatgaggagcgtggactccgcaaaactcctcaggagagacccctcccctcccacccacagccgtagacctacaaacctggctctcagaggaa……  **CDX2 site (after insertion)**  gccaggggtgttagtttaatccagaattccatttgggtctataaagacatattaaagacctattctctttgtttctagcactgtacctaagaaaataaactaaataaaatactcagtctctcataaaaactagagggacaagacttggttcatgaacattttatttatatcgtctagaaaaacaggccaacttcacagccaggaagccacacaattaaatctcattttcagctctaaggtgaggaaaattacattttagtttagccttgaggggtcaaaggttgccatttgaagtacaccacccagcactggcctccctttgctgtgtagacagggccttgcagtttctgttttccttggttctgagactttacacttgtccagtaaatgcgtagataaaagttgctttgaaagcaaacccagggagcaaggaaggaggaagactcttccccatgaggctgaattggacctgagaacggatccagggctgggctgttatggggacgcccaaagaaagcttctagaaggacttggagttctgtagttggagcggaagatgttgatgatactgctgggtactattcctcttttccctggcatcttcaccaccatgtgcttttctccacctttccatttctaggttaaaatttggtttcagaaccgcagagcaaaggaaaggaaaatcaacaagaagaagttgcagcagcaacagcagcagcagccgccgcagccgcctccgccgccaccacagcctccccagcctcagccaggtcctctgagaagtgtccaggagcccttgagtccggtgtcttccctgcaagcctcagtgcctggctctgtccctggggttctggggccaactgggggggtgctaaaccccactgtcacccagGCCACGAACTTCTCTCTGTTAAAGCAAGCAGGAGACGTGGAAGAAAACCCCGGTCCTatggtgagcaagggcgaggaggataacatggccatcatcaaggagttcatgcgcttcaaggtgcacatggagggctccgtgaacggccacgagttcgagatcgagggcgagggcgagggccgcccctacgagggcacccagaccgccaagctgaaggtgaccaagggtggccccctgcccttcgcctgggacatcctgtcccctcagttcatgtacggctccaaggcctacgtgaagcaccccgccgacatccccgactacttgaagctgtccttccccgagggcttcaagtgggagcgcgtgatgaacttcgaggacggcggcgtggtgaccgtgacccaggactcctccctgcaggacggcgagttcatctacaaggtgaagctgcgcggcaccaacttcccctccgacggccccgtaatgcagaagaagaccatgggctgggaggcctcctccgagcggatgtaccccgaggacggcgccctgaagggcgagatcaagcagaggctgaagctgaaggacggcggccactacgacgctgaggtcaagaccacctacaaggccaagaagcccgtgcagctgcccggcgcctacaacgtcaacatcaagttggacatcacctcccacaacgaggactacaccatcgtggaacagtacgaacgcgccgagggccgccactccaccggcggcatggacgagctgtacaagtgaggcgcgcccccacaggagtctgcagcagcagagcaattccaggctgagccatgaggagcgtggactccgcaaaactcctcaggagagacccctcccctcccacccacagccgtagacctacaaacctggctctcagaggaaaaatgggagccgggagtaagacaagtgggatttggggcctcaagaaatatactctcccagattttttactttttcccgtccggctctctctgccactgaggagacagagagccaccactgggcttcattcaggactggcagaagcattgcctggactgaccacaccaacgaggccttcagcctcctccccagctcttctcatcctagatctgcaggctgcacctctggctagagctgaggggagagagggacttaggggaacagcaagcttgagaccagcctgctcatggccctcggaggtccagctgggcctcctgcctccgggcaggcaaggtttacactgcagaagccaaaaaggcagctaagatagaaagttggactgaccaaagactgcagaacccccaggtggcctgtgtcttttttctcttcccttcccagaccaggaaaggcttggctggtgtatgcacagggtgtggtatgaggggtggttattggactccaggcctgaccagggggcccaaacagggacttgtttagagaacctgtcaccagagcttctctgggctgaatgtatgtcagtgctataaatgccagagccagcctggacttcctgtcattttcgcaatcttggggctgatga |
| **13, Monkey-H3.3B-mCherry-donor** |
| **H3.3B site (before insertion)**  ……tccccgagggggattaatagtgtggctcttgtcctcaacaggaggctagtgaagcgtacctggtgggtctgttcgaagatactaatctgtgtgccatccacgctaagagagtcaccatcatgcccaaagacatccagttggctcgccggatacggggagagagagcttaagtgaaggcagtttttatggcgttttgtagtaaattctgtaaaatactttggtttaatttgtgactttttttgtaagaaattgtttataatatgttgcatttgtacttaagtcattccatctttcactcaggatgaatgcgaaaagtgactgttcacagac……  **H3.3B site (after insertion)**  gtatggggtcgggaggtttccccttggggctttgttccggaaccgcgtccccgccttttcctggcggtgattcagaggtcccgacgccggattccgggcagtcgggcctccgcccgtcaaggaagggaagtgactcctccctgtactccccctcccgggggaggctgccgctccttgcagcctgagtcattaggggagggggaggaggttggcggccctcggccatctgccgccgcgaggtggggcacggggaggcgggaggcccggcggaggggccagggcctgttcccccgcagccccggggtgcggcccgggtgagcgcggccgccttatcttcggggcgtctttcttaggtgaaagaaaatggcccgaaccaagcagactgctcgtaaatccaccggtgggaaagcgccccgcaaacagctggccacaaaagccgccaggaaaagcgctccctctaccggcggggtgaagaagcctcatcgctacaggtaggccgggtgggggaacagtggcccgccggtggacggctttgtgcggcggcgtctgctcacccctcccctgctcgctgcaggcccgggaccgtggcgcttcgagagattcgccgttatcagaagtcgaccgagctgctcattcggaagctgcccttccagaggttggtgagggagatcgctcaggatttcaaaaccgacctgaggtttcagagcgcggccattggtgcgctgcaggtaagacaaaggcctggagtcgggggagggctgggcggtttccgctccccgagggggattaatagtgtggctcttgtcctcaacaggaggctagtgaagcgtacctggtgggtctgttcgaagatactaatctgtgtgccatccacgctaagagagtcaccatcatgcccaaagacatccagttggctcgccggatacggggagagagagctGCCACGAACTTCTCTCTGTTAAAGCAAGCAGGAGACGTGGAAGAAAACCCCGGTCCTatgcctaaaaagaaaagaaaggtgggttctggtgtgagcaagggcgaggaggataacatggccatcatcaaggagttcatgcgcttcaaggtgcacatggagggctccgtgaacggccacgagttcgagatcgagggcgagggcgagggccgcccctacgagggcacccagaccgccaagctgaaggtgaccaagggtggccccctgcccttcgcctgggacatcctgtcccctcagttcatgtacggctccaaggcctacgtgaagcaccccgccgacatccccgactacttgaagctgtccttccccgagggcttcaagtgggagcgcgtgatgaacttcgaggacggcggcgtggtgaccgtgacccaggactcctccctgcaggacggcgagttcatctacaaggtgaagctgcgcggcaccaacttcccctccgacggccccgtaatgcagaagaagaccatgggctgggaggcctcctccgagcggatgtaccccgaggacggcgccctgaagggcgagatcaagcagaggctgaagctgaaggacggcggccactacgacgctgaggtcaagaccacctacaaggccaagaagcccgtgcagctgcccggcgcctacaacgtcaacatcaagttggacatcacctcccacaacgaggactacaccatcgtggaacagtacgaacgcgccgagggccgccactccaccggcggcatggacgagctgtacaagaaaaggccggcggccacgaaaaaggccggccaggcaaaaaagaaaaagtgagtgaaggcagtttttatggcgttttgtagtaaattctgtaaaatactttggtttaatttgtgactttttttgtaagaaattgtttataatatgttgcatttgtacttaagtcattccatctttcactcaggatgaatgcgaaaagtgactgttcacagacctcagtgatgtgagcactgttgctcaggagtgacaagttgctaatatgcagaagggatgggtgatatttcttgcttctcatgatgcatgtttctgtatgttaatgacttgttgggtagctattaaggtactagaattgataaatgtgtacagggtccttttgcaataaaactggttatgacttgatccaagtgtttaacaattggggctgttaagtctgaccatacatcactgtgatagaatgtgggctttttcaagggtgaagatacaagtcttaaccacagtgtaacttacagtttcctttaaaaaaaaaaaaaaaaaaagtaaacctggcagctatagaatacactatgtgcatttataatagctattttatatattgtagtgtcaacatttttaaattaaatgttttacattcacaagtggtggggagtcttgtcattaaggtgtgtgtaatttagagtccagttggttttcttctgactgcacttgttctcatagtagtaaaatgctatgcgcatttataccttgcataagtcctcattctaccacatgttatctaaccctctagctgataatgcaaacactaactgggggat |
| **14, Mouse-Calcr-Cre-donor** |
| **Calcr site (before insertion)**  ……cagttcaagatccagtggagccaacgctggggaaggcgccgccgccccaccaaccgcgtagttagtgctcctcgggctgtagccttcgctgagccagatggcctccccatttacatctgccatcaggaaccacggaatcctccaatcagcaacaacgaaggcgaggagagtactgaaatgatccccatgaacgtcatccagcaagacgcatccgcttgaatgtgaagccaccccaagcattgtgatccactgagccttcatttcctggggaaagacagaccatgtgtttcaagtgattcccatcctcccaggagctgaccatatcatttg……  **Calcr site (after insertion)**  atcattttgattaaaacatgaaaggcagcaggtaagttttcttaactctttctacttactctctcttgttacctaaacaagtaactcgatatcttgattagcatattgatattcctagaaggtgtatttctttgcctcagtataaactcggttcttagatcagatctacaatgtaacctccattcgcctctgatgagccccaaaagtcatgtacataaggtttgctggggtcattgcccacagtctatggaaacaggctctctttgctcttgcaggtgcaagtcaccctgaagcgccagtggacgcagttcaagatccagtggagccaacgctggggaaggcgccgccgccccaccaaccgcgtagttagtgctcctcgggctgtagccttcgctgagccagatggcctccccatttacatctgccatcaggaaccacggaatcctccaatcagcaacaacgaaggcgaggagagtactgaaatgatcccAatgaacgtcatTcagcaagacgcatcTgctGCTACTAATTTCTCCTTGCTTAAGCAAGCTGGTGATGTTGAAGAAAATCCTGGTCCTatggccaatttactgaccgtacaccaaaatttgcctgcattaccggtcgatgcaacgagtgatgaggttcgcaagaacctgatggacatgttcagggatcgccaggcgttttctgagcatacctggaaaatgcttctgtccgtttgccggtcgtgggcggcatggtgcaagttgaataaccggaaatggtttcccgcagaacctgaagatgttcgcgattatcttctatatcttcaggcgcgcggtctggcagtaaaaactatccagcaacatttgggccagctaaacatgcttcatcgtcggtccgggctgccacgaccaagtgacagcaatgctgtttcactggttatgcggcggatccgaaaagaaaacgttgatgccggtgaacgtgcaaaacaggctctagcgttcgaacgcactgatttcgaccaggttcgttcactcatggaaaatagcgatcgctgccaggatatacgtaatctggcatttctggggattgcttataacaccctgttacgtatagccgaaattgccaggatcagggttaaagatatctcacgtactgacggtgggagaatgttaatccatattggcagaacgaaaacgctggttagcaccgcaggtgtagagaaggcacttagcctgggggtaactaaactggtcgagcgatggatttccgtctctggtgtagctgatgatccgaataactacctgttttgccgggtcagaaaaaatggtgttgccgcgccatctgccaccagccagctatcaactcgcgccctggaagggatttttgaagcaactcatcgattgatttacggcgctaaggatgactctggtcagagatacctggcctggtctggacacagtgcccgtgtcggagccgcgcgagatatggcccgcgctggagtttcaataccggagatcatgcaagctggtggctggaccaatgtaaatattgtcatgaactatatccgtaacctggatagtgaaacaggggcaatggtgcgcctgctggaagatggcgattgatgaatgtgaagTcaccccaagcattgtgatccactgagccttcatttcctCCggaaagacagaccatgtgtttcaagtgattcccatcctcccaggagctgaccatatcatttgtgaagaagtgttaagtgaatttgtccatagtgaatttgaagaaagtgattcttggtactattgctttgggagtcagtctaggaatagagtctcccattgcaacttgtgaactccatcattcatcctggactgagatgactgtgttcgtaggaaagcaggcaaggtgttcaaaagatgtcacactattgacctagttcagatacagggtgctccttgtgaattttgagccatttgtaccgttgagaaattaaaatcactctcaatgtttttaaatttgacactggattttgaattagactatttctgtatttgactacagatctggtttttaatgtttttatttcagtcagttcctatattacacatgttaccatccatacaatgtc |
| **15, Mouse-Lypd1-Cre-donor** |
| **Lypd1 site (before insertion)**  ……tgtgcatcagctgctgcaacacccctctttgcaatgggccgaggcccaagaagagaggcagctctgcctcggccatcaggccagggcttctcaccactctcctgttcttccacttagccctctgcttggcacactgctgaagctaaaggagatgccaacccctgctgcctcacctgtctggcccttcgtctctcaccttcccgagtctcttctgggtgtccttttattctgggtagacaagggagtctttttgttccctcctttcaagtaacgcaagattgccgtgcacaaatacttttgtaagctctgaaccaattcattctgaatttc……  **Lypd1 site (after insertion)**  ccacttattaggtgataatgaaaataatatctggatatgttgtctgtgctgctagcgtgagatggtttaaacagtaacaaaggaaagaaaaaccttgttgtctagtataaattaggagctgactttaaaatagacattgttgctggagacatggctgcatcggtaatgtgtttgccattgcaaccatgagggcttaactttctttgatccttagaacccatattaaaaagctgagtgtaggggcaggagagatggttacaaggatgcagaggacttgagtttagctcccaacacctccttaagacagctcacaactgcctgtaactccaattccaaacgaatccaattccctcttctggaatctgaggatactgcactcacatatacacatacattgcatacatgaacatatatacatacattgcatgtatgaacatatacacatgcattgcacacacagagtggtgaggggagactttgattttaatagctctttattcatggacaccagcaactaaaatctctgttggaacaacattcaccataagttctgtgtgtttggtgacctccatgcccacccaaaagaactcccagactccaataagtctgagaattcttatcccctgaaccatctcccagatcaggagataagagccctttctgtgggaggagttaaggaaaggttatgcactcagatttgggcacaaagagatagctggactgcagatcacatatgctaaccccacttctctgggcctgtgttatagggatcatgtaccggaagtcgtgtgcatcgtcagcagcctgtctcattgcttcagctgggtaccagtccttctgttcccctgggaaactgaactccgtgtgcatcagctgctgcaacacccctctttgcaatgggccgaggcccaagaagagaggcagctctgcctcggccatcaggccagggcttctcaccactctcctgttcttccacttagccctctgcttAgcacactgcGGATCCGGAGCTACTAATTTCTCCTTGCTTAAGCAAGCTGGTGATGTTGAAGAAAATCCTGGTCCTatggccaatttactgaccgtacaccaaaatttgcctgcattaccggtcgatgcaacgagtgatgaggttcgcaagaacctgatggacatgttcagggatcgccaggcgttttctgagcatacctggaaaatgcttctgtccgtttgccggtcgtgggcggcatggtgcaagttgaataaccggaaatggtttcccgcagaacctgaagatgttcgcgattatcttctatatcttcaggcgcgcggtctggcagtaaaaactatccagcaacatttgggccagctaaacatgcttcatcgtcggtccgggctgccacgaccaagtgacagcaatgctgtttcactggttatgcggcggatccgaaaagaaaacgttgatgccggtgaacgtgcaaaacaggctctagcgttcgaacgcactgatttcgaccaggttcgttcactcatggaaaatagcgatcgctgccaggatatacgtaatctggcatttctggggattgcttataacaccctgttacgtatagccgaaattgccaggatcagggttaaagatatctcacgtactgacggtgggagaatgttaatccatattggcagaacgaaaacgctggttagcaccgcaggtgtagagaaggcacttagcctgggggtaactaaactggtcgagcgatggatttccgtctctggtgtagctgatgatccgaataactacctgttttgccgggtcagaaaaaatggtgttgccgcgccatctgccaccagccagctatcaactcgcgccctggaagggatttttgaagcaactcatcgattgatttacggcgctaaggatgactctggtcagagatacctggcctggtctggacacagtgcccgtgtcggagccgcgcgagatatggcccgcgctggagtttcaataccggagatcatgcaagctggtggctggaccaatgtaaatattgtcatgaactatatccgtaacctggatagtgaaacaggggcaatggtgcgcctgctggaagatggcgattgatgaagctaaaggagatgccaacccctgctgcctcacctgtctAgcccttcgtctctcaccttcccgagtctcttctgggtgtccttttattctgggtagacaagggagtctttttgttccctcctttcaagtaacgcaagattgccgtgcacaaatacttttgtaagctctgaaccaattcattctgaatttctgtgtgtagttgaagaaaaaagcatggagcagaaagtccagaccctcccatcccaatctggttaaccaccgccaaggctagcctggaagaaccagcccttagaagtcattgagatacgcatctgcctttcccaaagccttgagcttccattctgtcccagtaggagtcacagtctattcagagactgctgctgcgtgaaggtaactttgcttttgcgggaggggagagccagtttcggctcaaggcttctgaacttgccattcatacttcctgctcctgtaaactattttctggggtggacccagctggtttggtctctgagccagtctgtggtgactcaggactcaagggctggggcttagcctctccaggcttggcctcagtctgaaaagtgcttaagaaaaccttgttagttctcctggaggaagagttactgcgccgggaggctaggaagatgagggggctgcgggctgagctggtgctgtccttggtggagatgaagcgggcacgctggcgtttctcttggttggcatgctgcagagtcaggcggcagcagagcacctgccagaacaccttccggaactgctgagaggacacgttgtagaggagagggttgaccacagagctgaggtagaagaaggtatcagagaagggcaggaggatcatgtatgccctgaagtacgttctggtccagtcatgtttgggttttgctgcagccatgatccgtcggatctgattgggcatccaacacacggccaacgtcaccacaatcagtcctggcaggcaagaacaggagagaaaagg |
| **16, Mouse-Mllt3-LoxP-donor** |
| **Mllt3 site (before insertion)**  ……gggctgaaaattgggcctgatctagatggtagaatgtttatgtttttaacatctttctgtctgttgttctcatcttccctgttttttcttttaacattgcagtgtgcaaagacccaccttacaaggtagaagagtccgggtacgctggcttcatcttgccaattgaagtttattttaaaaacaaggtatgtaatctttacctgctattccttcagaatcctgtgttaaccagataatgcttaatataatccattaataattctaatgctatatagtgctaaagctaagcttgaattcttagaatgttggttatcacaacttttaa……  **Mllt3 site (after insertion)**  **ctcgag:**XhoI  **ggatcc:**BamHI  ataacttcgtatagcatacattatacgaagttat:LoxP  tggatgctggatactggaacttaggtctcttgcaagagcaagtgttcttaacttccctgcaggctttccagcccacaagacccagtgagattcatttggcttgtgattgttttactatagtagaaggtttagaagaatgaaattttagtccccaaggactctgattctctaagaccttcgtttattttaagaaaaaaaataactatttggttgttttttaaaaatgtgttgttgtgggatctcattaaacctcattttgtggcatcagaaagtgggagcgatcaattctactgtgcctgagaagtgacagagcgtgggtttcaggtctatcaagcatctaggacatcttagaggacagaggcctaggcagcgaggaagtttcagtaactcccagtttggggtgtttcttttgttaggaaactctgtagttcttttgttcccgtggctctcaagcttcctgactctttaagatagttccccatgtggtggtgacccccaaccataaaattgcactgttacttcataactgtaattttgctagttgttatgaattataatgtaaatatctgctctacaggatgatcttaggcaatccctgtgagagggccattcaaccccagagggatcgtaacccacagggagagaaccaatatttacttttcttgaggttggatcacagactctgtcctgtttctgagcataattaagtccctcactgaccataactgatggtagtgagctacactccactcagattttaatttaaaaatatagatttggttttagataggaaaatattgctatgggtcccagagaagaagaaataaagaaagcagaatctcgactgtgaaatggtcagtggggctgaaaattgggcctgatctagatggtagaatgtttatgtttttaacatctttctgtctgttgttctcatcttcc**ctcgag**ataacttcgtatagcatacattatacgaagttatctgttttttcttttaacattgcagtCtgTaaagaTccTccGtaTaaAgtGgaGgaAAGCggCtaTgcCggGttTatTCtgccGatCgaGgtCtaCttCaaGaaTaaAgtatgtaatctttacctataacttcgtatagcatacattatacgaagttat**ggatcc**gctattccttcagaatcctgtgttaaccagataatgcttaatataatccattaataattctaatgctatatagtgctaaagctaagcttgaattcttagaatgttggttatcacaacttttaataatagtggctataaaaagataatttcaatcatattgtatctgtttatataaaattcactaaatactcctttgaagagtgattgccattctgaaaaagatggagatgtttgtaaagtagaaaagagaaattagagttttccaagaaatgtttctattcctgctttatctctagaacttttcaaaaataaaatcctgtattacctttagctgatgaacatacgtggccagtatataaagttatttcagcctgaaatagtttggattctattgcacttaaaagatgccactatgtttagggcagactctacagactgcaaggcatgggtgatggttgatggtctctgcgcttacttcattcatggcacggtgaggtaggagtgtccttctcagaaagtcgtcagaaggagtctgtgtactgaccatatgtgggatattcggagcattttttaagtaaacatttcttaagtgctgaacttcttttcagcattattgttccagggttttgtttataaacactatagactcttactttttatcttagctctatacattttgggcacaagaaggtaccatattgacacaataaactggagtggtctgtgcgtgagtgatgaagttagtacccttaccaccaagttcccatgccctccgctgccaaagctattgcttgctgctttttctctgtattcattggatcagtgcttttgcttacccacagcaaagcattcaa |
