# Supplementary material for: CATI: an efficient gene integration method for rodent and primate embryos by MMEJ suppression
Source: Genome Biol. 2023 Jun 23;24:146. doi: 10.1186/s13059-023-02987-w (PMC10288798; doi:10.1186/s13059-023-02987-w)
Supplement: Supplementary file 6 — Additional file 6. Review history. [file 13059_2023_2987_MOESM6_ESM.docx]

Review History

**First round of review**

**Reviewer 1**

**Were you able to assess all statistics in the manuscript, including the appropriateness of statistical tests used?** Yes, and I have assessed the statistics in my report.

**Comments to author:**

In this work, Chen et al. developed CasRX-assisted targeted integration (CATI) that enabled HDR efficiency improvement in mouse and monkey embryos by down-regulating the expression of a MMEJ critical factor polymerase Q. Several studies focusing on HDR efficiency improvement have been by NHEJ inhibition, but the elevation of efficiency is limited. The authors found that MMEJ inhibition could significantly improve HDR efficiency. This novel finding provided a better approach to achieve knock in animal models. Overall, this study is well-executed and the manuscript is well-written the data are sound. Thus, I think this work is suitable for publication in Genome Biology after proper revision. Below I provide more specific comments:

1. The authors should introduce the current methods used to improve HDR efficiency in more detail and illustrate their advantages and disadvantage in the introduction section.

2. To characterize gene expression profile of DSB repair-related genes during embryo gene editing, the authors chose Actb gene to performed RNA-seq analysis of embryos. Why chose Actb, rather than Calcr, which showed highly MMEJ-based repair?

3. In Figure S1B, more proper controls for this experiment should be used, including Cas9 only, mock (non-targeting sgRNA) and PBS. This will allow exclusion of the influence of gene editing system.

4. In Figure S1B, does a dot represent an embryo? How many embryos in each group?

5. Are the embryos injected with Cas9/sgRNA/donor DNA (for mCherry knock-in at Actb) mCherry-positive? RNAseq analysis of mCherry-positive and mCherry-negative embryos should be performed separately.

6. Line 15 of page 8, "Figure S1G" should be "Figure S1H".

7. In Figure S1H, all embryos were able to develop to blastocyst, it's unbelievable. The authors should explain the possibility.

8. In Figure 2A, statistical analyses should be performed.

9. Line 11 of page 9, the reference of using donor with biotin modification is incorrect.

10. The authors mentioned that HDR efficiency in 4-cell injection group is the lowest among zygote, 2-cell and 4-cell injection groups in monkey embryos. Can the authors further explain it in the text?

11. The figures of introducing clinically relevant mutation in monkey embryos are missing.

12. Statistical analyses should be performed in Figure S4C.

13. In the last result, the figures and text don't match.

**Reviewer 2**

**Were you able to assess all statistics in the manuscript, including the appropriateness of statistical tests used?** Yes, and I have assessed the statistics in my report.

**Comments to author:**

In this manuscript entitled "An Efficient Gene Integration Method in Rodent and Primate Embryos by MMEJ suppression", Dr. Zhen Liu and colleagues found dominant MMEJ occurs in embryos editing and developed CasRx-based Polq knock down method (CATI) to increase the HDR efficiency using either dsDNA or ssODNs donors. The manuscript provides an improved and efficient HDR editing method in both mouse and monkey embryos. However, there are still several major concerns needed to be addressed before consideration for publication in Genome Biology.

Majors:

1. Most previous publications have investigated DNA damage repair pathways using in vitro culture systems. In particular, the dynamics and competition of CRISPR-mediated NHEJ, MMEJ and HDR editing have been detailed previously. They have reported predominant repair by NHEJ instead of MMEJ in almost all the cases. This discrepancy should be discussed. This reviewer speculates that embryo injection is associated with delivery of hyper quantities of CRISPR components, which leads to further recognition and cleavage of target DNAs with small indels. At the same time, delivery of hyper quantities of donor DNA may need to random integration in the genome. As such, it is advisable to investigate this possibility in offspring.

2. CRISPR‒Cas9-mediated DSBs in human embryos may lead to chromosome loss (Ref: Allele-Specific Chromosome Removal after Cas9 Cleavage in Human Embryos), which limits embryo gene editing safety for clinical use. The authors also need to provide data to address this concern. In addition, delivery of hyper quantities of CRISPR components can also lead to off-target cleavage and large deletions. The levels of large deletions after double-strand breaks in different cell types appear to be different. It is curious to know the levels of large deletions in embryo editing.

Minors:

1. MMEJ was designated when microhomologies were observed surrounding the break site. The length of microhomologies is usually more than 2 bp (Ref: Decoding non-random mutational signatures at Cas9 targeted sites). In the Introduction session, the authors mentioned MMEJ and cited a reference (Ref: MMEJ repair of double-strand breaks (director's cut): deleted sequences and alternative endings). In this reference, the length of microhomologies was 5-25 bp. This reviewer encourages the authors to examine multiple relevant papers and carefully define MMEJ.

2. There are multiple citation format inconsistencies: We believe the indel reduction will benefit to genome stability and integrity (Hsu et al., 2014; Wen et al., 2021). embryo injection and culturing, has been mentioned in previous publication (Liu et al., 2018). reliability of which has been confirmed by several studies (Wilde et al., 2021).

3. Dozens of grammar errors or typos have been spotted.

**Reviewer 3**

**Were you able to assess all statistics in the manuscript, including the appropriateness of statistical tests used?** No, I do not feel adequately qualified to assess the statistics.

**Comments to author:**

Chen et al demonstrate three major things in their study. (1) They show that MMEJ is the predominant DNA repair process, but not NHEJ, in mouse zygotes. (2) They identify that PolQ is important for this process and (3) they test their idea of increasing the donor DNA insertion efficiency by down-regulating PolQ (using RNA editor CasRX) at a few loci.

Below are review comments and suggestions.

One major question that is perplexing is that most of their donors contain several hundreds of bases of homology arms. If MMEJ relies on micro homologies of only a few bases of complementarity, the question is how can authors attribute MMEJ helps enhance the HDR (of donors containing hundreds of bases of homology arms).

The authors used linear double stranded DNA donors for HDR experiments containing ~800 base homology arms. What is the rational or reason for choosing longer homology arms? Considering that MMEJ requires only short homologies, have authors tested shorter homology arms? Do longer or shorter homology arms matter? Related to this, long ssDNAs (with short homology arms) have been popularly used as donors for creating conditional knockout and Cre- or reporter- knock-in mouse models. Have authors tested how lssDNA donors perform in their CATI method. In other words, can MMEJ inhibition further enhance knock-in efficiency of lssDNA donors?

The authors conclude that off target effects are not increased when PolQ is suppressed. However, the analysis is performed for only one locus/guideRNA. If authors claim this as one of the conclusions of their study, they should do this analysis for all guideRNAs used in the study to know if it is really the case. In general, off target analysis is not a major concern in case of mouse model generation because (a) it is well established that they do not occur at frequencies that one needs to spend time and resources in finding them (b) even if off target effects are present in founder mice they can be segregated by breeding.

On the other hand, what is more important, specifically for this work, is to find out the rate of off target donor insertions (i.e., random insertions of exogenously supplied DNA templates). Testing off target insertions (not just off target Cas9 effects) would be important particularly because DNA repair pathway is perturbed (by suppressing PolQ).

This reviewer suggests that full sequences of all donor DNAs (dsDNAs and ssODNs) along with their annotations (demarcating homology arms, location of guide sequences with respect to new sequences added) and genotyping PCR primers should be provided as schematics. Such information can be best presented as schematics with color coded sequences for different regions.

Instead of 'marked elevation' in this sentence in the abstract (By down-regulating the expression of a MMEJ critical factor Polymerase Q (Polq) via RNA editor CasRX, we achieved a marked elevation in the targeted integration efficiency of linearized DNA fragments and single-strand

oligonucleotides (ssODN) in mouse embryos and offspring, without increasing offtarget

effects), the authors should indicate the increase in quantitative terms (for example 'fold increase'). Also, if the phrases 'without increasing offtarget effects' is retained, such analysis should be done for all loci/guides including off target insertions.

The authors note that "We speculated that the latter was caused by the presence of large amounts of linearized donor DNA, which may be recognized by embryos as DNA damage." In regard to the experiment S1B and S1C, an important control would be NOT to create double stranded break in the genome. This can be done by excluding Cas9 and only inject guide and the donor DNA (?).

Minor comments:

Introduction and discussion sections mention a few DNA repair pathway papers. Those papers are predominantly related to cell culture experiments (and the discussion of animal model generation methods/papers is somewhat low in these sections). Considering that all experiments (of testing of the new method of modulating MMEJ pathway) pertain to generation of animal models, the introduction and discussion sections can be expanded a bit more to include some of the commonly used methods of creating knock-in mouse models and discuss the challenges of those methods. The authors indeed mention this challenge in the cover letter, which can be added to the manuscript and expanded a bit more.

Figure 1C; If I understand correctly, the sequence used (to describe the concepts of three types of DNA repair) is hypothetical as I could not find a classical PAM sequence close to the repaired nucleotides (shown in red). Perhaps authors can use one of their actual examples (guide and the repair outcomes) to describe the idea?

Figure S2D. Number of zygotes transferred to each surrogate mother is indicated (which is 20 for all mice) but number of zygotes injected need to be included.

The authors have manually calculated NHEJ/MMEJ efficiencies using ICE data (described in pages 20-21 of merged pdf file). They present this data as charts in figure 1 and S1, and this data forms the basis for their main conclusion. Could the authors also provide details how they did the manual analysis and include the intermediary files of information as supplemental files?

A few sentences containing typos/grammatical errors are copied below, as examples but the manuscript needs English editing throughout.

We found that MMEJ was a predominate form in nuclease-mediated DSB repair (predominant)

Thus, the CATI method could be a prior option for developing gene-edited monkey models and human germ-cell gene therapies (did author mean 'preferred option', not 'prior option'?)

Although it is reasonable that transiently knockdown of Polq might induce limited harmful effect, we still performed Polq knockdown test to examine whether embryos develop normally (transient)

As results shown, embryos with…. ('As shown in results' could be better?)

Overall, all targets showed an increased gene integration efficiency in varying degree (Fig. 2B). (….varying degrees)

**Authors Response**

**Point-by-point responses to the reviewers’ comments:**

**Reviewer #1**: In this work, Chen et al. developed CasRX-assisted targeted integration (CATI) that enabled HDR efficiency improvement in mouse and monkey embryos by down-regulating the expression of a MMEJ critical factor polymerase Q. Several studies focusing on HDR efficiency improvement have been by NHEJ inhibition, but the elevation of efficiency is limited. The authors found that MMEJ inhibition could significantly improve HDR efficiency. This novel finding provided a better approach to achieve knock in animal models. Overall, this study is well-executed and the manuscript is well-written the data are sound. Thus, I think this work is suitable for publication in Genome Biology after proper revision. Below I provide more specific comments:

**Responses**: We are grateful that the reviewer acknowledges the novelty, rigor, and clarity of our study. Additionally, we extend our sincere thanks for the insightful feedback and inquiries, which have significantly contributed to the enhancement of our manuscript.

1. The authors should introduce the current methods used to improve HDR efficiency in more detail and illustrate their advantages and disadvantage in the introduction section.

Response: We express our gratitude to the reviewer for their constructive recommendations. In accordance with these suggestions, we have expanded the introduction and discussion sections within the revised manuscript to provide a more comprehensive context.

Revised text: “Despite the advances in genome editing, the efficiency of precise DNA fragment integration through HDR remains suboptimal. Recently, several methods have been reported to enhance gene knock-in efficiency in zygotes via Cas9 (mRNA or ribonucleoprotein) injection. These approaches include the utilization of transgene templates cleavable into 800 bp homology arms in vivo (HMEJ strategy) [6], linearized templates derived from PCR amplification (Tild-CRISPR) [7], long single-stranded DNA (ssDNA) templates (Easi-CRISPR) [8], short ssODN templates[9], templates that activate the MMEJ or NHEJ pathways [10-12], and templates modified with Biotin-avidin system (CAB system) [13, 14]. Furthermore, chemically modified single guide RNAs (sgRNA) [15] and commercially available, chemically modified tracrRNAs and crRNAs [16, 17] have also been employed to augment gene knock-in efficiency. However, only a limited number of successfully targeted loci have been documented thus far [8, 12, 18]. Certain methods, such as Easi-CRISPR and ssODN, are constrained by the size limitations of DNA fragments for insertion (typically <1 kb). Other approaches, including those based on MMEJ or NHEJ templates, are restricted by the generation of imprecise junctions at editing sites. Additionally, methods involving modified templates or sgRNAs may be hindered by cytotoxicity. Furthermore, techniques employing long ssDNA-based Easi-CRISPR are constrained by challenges in obtaining templates and elevated costs.” Line 46 to Line 64 of the revised manuscript.

2. To characterize gene expression profile of DSB repair-related genes during embryo gene editing, the authors chose *Actb* gene to performed RNA-seq analysis of embryos. Why chose *Actb*, rather than *Calcr*, which showed highly MMEJ-based repair?

Response: We express our gratitude to the reviewer for their insightful inquiry. The primary rationale behind selecting the *Actb* gene is its widespread recognition and representation, owing to its robust expression levels. This characteristic allows for facile observation and manipulation. Notably, numerous studies have also employed the *Actb* site as a reporter in the assessment of various knock-in (KI) methodologies. (*ref: In vivo genome editing restores haemostasis in a mouse model of hemophilia; ref: RS-1 enhances CRISPR/Cas9- and TALEN-mediated knock-in efficiency; ref: Increasing the efficiency of precise genome editing with CRISPR-Cas9 by inhibition of nonhomologous end joining*). Regarding the *Calcr* site, it exhibits the highest MMEJ repair ratio, rendering it an atypical example for illustrative purposes. Simultaneously, the CATI method remains effective at the *Actb* site, despite the corresponding guide RNA not displaying a substantial MMEJ bias. These findings demonstrate the broad applicability of the CATI method, and as such, the selection of the *Actb* site as the experimental reference point is more appropriate in comparison to the *Calcr* site.

3. In Figure S1B, more proper controls for this experiment should be used, including Cas9 only, mock (non-targeting sgRNA) and PBS. This will allow exclusion of the influence of gene editing system.

Response: We express our gratitude to the reviewer for their thoughtful suggestion and concur with the notion that incorporating more appropriate controls will enable the exclusion of potential influences from the gene-editing system. In accordance with the reviewer's recommendation, we have designed and executed a new series of RNA-seq and RT-qPCR experiments to assess the expression levels of DSB repair-related genes. For each experiment, we established seven distinct conditions as follows: (1) blank; (2) dilution solvent; (3) Cas9 only; (4) Cas9+scramble sgRNA; (5) Cas9+sgRNA; (6) donor only; (7) Cas9+sgRNA+donor. The injection procedure and sample preparation were consistent with those described in the original manuscript.

In the RNA-seq results, we observed that the expression levels of most canonical DNA damage repair-related genes did not exhibit significant alterations, which aligns with previous findings (see revised Figure 1A and 1B). The “blank” group and “dilution” group displayed similar *polq* levels, approximately half that of the “Cas9+sgRNA+donor” group, which is consistent with the data presented in the original manuscript. However, we noted an upregulation of *polq* levels in the “Cas9+sgRNA” group in this experimental iteration, a finding not observed in the original manuscript's data. We speculate that the disparity between the two datasets may stem from the low expression of *polq* in embryos (see revised Figure 1C and 1D). A re-analysis of a published RNA-seq database (GSE66582) similarly revealed low *polq* expression levels (FPKM < 1) (see revised Figure 1E).

In the RT-qPCR experiment, which offers higher sensitivity for detecting low expression genes, we observed an upregulated *polq* level not only in group “(7) Cas9+sgRNA+donor” but also in group “(6) donor only” (see revised Figure 1F). The elevated *polq* level in the “Cas9+sgRNA+donor” group aligns with our previous data from the original manuscript. The increased *polq* level in the “donor only” group further corroborates our speculation in the main text, stating, “We speculated that the latter was induced by the presence of substantial quantities of linearized donor DNA, which may be recognized by embryos as DNA damage.” Overall, our results indicate that the editing system, encompassing Cas9, sgRNA, and dilution buffer, imparts minimal influence on the expression of the majority of DNA damage repair genes. We have now integrated the updated data into the revised manuscript as Fig. 1F and have modified the corresponding text from line 156 to line 160 to reflect these changes.

**Revised Figure 1.** DNA repair-related gene expression. (A) Principal component analysis of transcriptomes from seven group samples. Each dot represents one sample, and each sample contains five embryos. (B) The expression of analyzed DNA repair-related genes from seven groups. DNA repair pathway include HR, NHEJ, SSA and MMEJ. (C) and (D) The relative FPKM value of *polq* from seven groups. (E) The relative FPKM value from data GSE66582. (F) The relative expression level of indicated genes (*Rad52*, *Ku70* and *polq*). *p < 0.05, **p < 0.01.

4. In Figure S1B, does a dot represent an embryo? How many embryos in each group?

Response: We express our gratitude to the reviewer for raising these questions and apologize for any oversight in addressing the details. In Figure S1B, principal component analysis (PCA) was conducted on the transcriptomes from the three sample groups. Samples within the same group clustered together, with each dot representing a single sample comprising five embryos. In the revised manuscript, we have provided a more comprehensive description in the Figure legend accompanying Supplementary Material S1B.

5. Are the embryos injected with Cas9/sgRNA/donor DNA (for mCherry knock-in at Actb) mCherry-positive? RNAseq analysis of mCherry-positive and mCherry-negative embryos should be performed separately.

Response: We express our gratitude to the reviewer for their thoughtful suggestion and acknowledge the insight behind their recommendation. However, we sincerely apologize for the infeasibility of conducting the proposed experiment and for any imprecise descriptions that may have led to misunderstandings. As is well-known, DNA repair is a rapid response process that addresses DNA damage. In our experimental design, the injected embryos were collected 10 hours post-injection, as the majority of DNA damage repair responses occur within a 3~8 hours window following injection. Within such a short timeframe, mCherry fluorescence would not be observable, even if the knock-in (KI) process were completed. Consequently, the Cas9/sgRNA/donor DNA (for mCherry knock-in at *Actb*) merely represents the group of reagents fulfilling the requirements for gene knock-in at the *Actb* site. in order to mitigate the possibility of misinterpretation by readers, we have incorporated a clarifying statement within the text, specifically in lines 142 through 143.

6. Line 15 of page 8, "Figure S1G" should be "Figure S1H".

Response: We express our great gratitude to the reviewer for the careful review of our manuscript. We are sorry for the error and we have made the correction in the revised manuscript.

7. In Figure S1H, all embryos were able to develop to blastocyst, it's unbelievable. The authors should explain the possibility.

Response: We are grateful to the reviewer for bringing this matter to our attention and would like to provide an explanation. In our laboratory, we typically achieve an 85%-100% blastocyst rate when culturing superovulation and mating-derived B6/DBA F1 mouse zygotes. The remaining 0-15% of embryos often arrest before the 2-cell stage due to poor oocyte quality. To mitigate potential effects resulting from variations in low-quality embryos, we exclude zygotes exhibiting abnormal morphology, such as large perivitelline space, fragments, or premature cleavage. Only zygotes meeting stringent morphological standards are utilized for subsequent injections, which we believe enhances the reliability and repeatability of our results.

To further address this concern, we conducted a separate experiment to culture two types of embryos. As the figure demonstrates, the “normal” embryos exhibited a 98/98 blastocyst rate, whereas most of the “abnormal” embryos failed to progress to the blastocyst stage (see revised Figure 2). Consequently, it is reasonable for our procedure to yield a blastocyst proportion approaching ~100%.

**Revised Figure 2**. The picture shows the morphological characteristics and development status of normal and abnormal embryos.

8. In Figure 2A, statistical analyses should be performed.

Response: We express our gratitude to the reviewer for their thoughtful suggestion. In the original manuscript, we solely calculated the ratio of mCherry-positive embryos relative to the total number of embryos. We have now conducted further repetitive experiments and incorporated statistical analysis for these results (see revised Figure 3). Additionally, we have replaced Fig. 2A in the revised version of the manuscript.

**Revised Figure 3.** Statistical analysis was performed to compare the mCherry gene knocking efficiency among different experimental groups at Actb and Gata6 sites.

9. Line 11 of page 9, the reference of using donor with biotin modification is incorrect.

Response: We are grateful to the reviewer for their meticulous review and for identifying this imprecise citation. While the reference we cited, “Efficient generation of targeted large insertions by microinjection into two-cell-stage mouse embryos” did employ biotin-modified donor DNA in their research, it was not the pioneering study that introduced this method. We have now incorporated additional pertinent references in the revised manuscript “Efficient generation of mice carrying homozygous double-floxp alleles using the Cas9-Avidin/Biotin-donor DNA system.”, as shown in lines 739 to 741.

10. The authors mentioned that HDR efficiency in 4-cell injection group is the lowest among zygote, 2-cell and 4-cell injection groups in monkey embryos. Can the authors further explain it in the text?

Response: We express our gratitude to the reviewer for their insightful question and suggestion. Initially, we were quite surprised to find that the 4-cell injection exhibited the lowest efficiency, as we had anticipated that this stage would yield improved efficiency. One possible explanation is that the 4-cell monkey embryo might have a short G2 phase during which homology-directed repair (HDR) occurs. Furthermore, while zygotic genome activation in mice takes place at the 2-cell stage, it occurs at the 8-cell stage in primates. Consequently, the open chromatin accessibility in monkey 4-cell stage embryos may not yet be fully established. We have incorporated this speculation into the revised manuscript, as shown in lines 264 to 269.

11. The figures of introducing clinically relevant mutation in monkey embryos are missing.

Response: We appreciate the reviewer’s meticulous attention to detail and for identifying this oversight. We apologize for our negligence in providing such crucial information. We have now included the corrected image as Figure S4A, S4B, S4C in the revised manuscript (see in Revised Figure 4).

**Revised Figure 4.** Clinically relevant mutation in monkey embryos CDKL5 locus. (A)	Illustration of monkey CDKL5 locus for nucleotide replacement. Replaced nucleotide is shown in red. BE, ABE, CBE cannot be used for G-to-A mutation. (B) Positive rate of editing monkey embryos for R178Q mutations using baseline and CATI strategies. (C) Proportion of HDR and MMEJ events of edited monkey embryos at CDKL5 locus.

12. Statistical analyses should be performed in Figure S4C.

Response: We express our gratitude to the reviewer for their careful reading and for bringing this matter to our attention. We have now incorporated the statistical analysis in the revised version’s Figure S4F, which corresponds to the original manuscript’s Figure S4C.

13. In the last result, the figures and text don't match.

Response: We express our great gratitude to the reviewer for the careful reading. As per the reviewer suggestions, we have diligently revised the manuscript to ensure that the Figure and corresponding text are in alignment. We sincerely apologize for any inconvenience caused due to our inadvertent oversight.

**Reviewer #2:** In this manuscript entitled "An Efficient Gene Integration Method in Rodent and Primate Embryos by MMEJ suppression", Dr. Zhen Liu and colleagues found dominant MMEJ occurs in embryos editing and developed CasRX-based Polq knock down method (CATI) to increase the HDR efficiency using either dsDNA or ssODNs donors. The manuscript provides an improved and efficient HDR editing method in both mouse and monkey embryos. However, there are still several major concerns needed to be addressed before consideration for publication in Genome Biology.

**Responses**: We are grateful that the reviewer recognizes our study as an improvement in providing an efficient HDR editing method for both mouse and monkey embryos. We also express our appreciation for the reviewer's valuable comments and questions, which have enabled us to further enhance our manuscript.

**Majors:**

1. Most previous publications have investigated DNA damage repair pathways using in vitro culture systems. In particular, the dynamics and competition of CRISPR-mediated NHEJ, MMEJ and HDR editing have been detailed previously. They have reported predominant repair by NHEJ instead of MMEJ in almost all the cases. This discrepancy should be discussed.

Response: We thank the reviewer for the kind suggestion. In our prior manuscript, we categorized MMEJ events as those accompanied by microhomology sequences ranging from 1 to 25 nucleotides (1~25 nt). Through this classification, we found 61/88 (69.3%) sgRNA-guided editing showed MMEJ-biased repair with a NHEJ/MMEJ frequency ratio less than 1(see in revised Figure 5A). As a result, we concluded that MMEJ is dominant in embryo gene editing.

In the revised manuscript, we reclassified the MMEJ and NHEJ repair pattern. multiple methods currently exist to define MMEJ repair, and a definitive standard has yet to be established. We have integrated the relevant MMEJ classification definitions from some of existing literature and restructured the updated manuscript (*Ref: Decoding non-random mutational signatures at Cas9 targeted sites; Ref: Dynamics and competition of CRISPR–Cas9 ribonucleoproteins and AAV donor-mediated NHEJ, MMEJ and HDR editing; Ref: MMEJ repair of double-strand breaks (director's cut): deleted sequences and alternative endings; Ref: Synthesis-dependent microhomology-mediated end joining accounts for multiple types of repair junctions; Ref: Microhomology-mediated end joining: a back-up survival mechanism or dedicated pathway?*). In accordance with prior research, we reclassified the DNA repair pattern as NHEJ when insertion occurred, or in cases where microhomology sequence were not detected in vicinity of the deletion segment, or when the deletion involved only a single base. Conversely, deletion events accompanied by microhomology sequences (2~25 nt) were classified as MMEJ repair pattern (see in Revised Figure 8A and 8B). In the data analyzed according to the new classification method, we discovered that 18 out of 88 (20.45%) sgRNA-guided modification exhibited a MMEJ-biased repair pattern, characterized by a NHEJ/MMEJ frequency ratio less than 0.5. Among other 70 sgRNA, 44.29% (31/70) showed a 0.5~2 NHEJ/MMEJ ratio, indicating that MMEJ also plays a significant role in the repair of CRISPR-mediated DSBs damage in embryos (see in Revised Figure 5B). Furthermore, we also observed that higher sgRNA editing efficiency corresponds to a larger proportion of MMEJ repair patterns. When sgRNA editing efficiency is less than 66.7%, 16% (8/50) sgRNA-guided modification show a preference for the MMEJ repair pattern. Conversely, when sgRNA editing efficiency exceeds 66.7%, 26% (10/38) sgRNA-guided modification display a preference for the MMEJ repair pattern. (see in Revised Figure 5B). These findings imply that, while we cannot conclude that MMEJ plays a dominant role in embryonic gene editing, it may still have a significant function in the repair of nuclease-mediated DSBs in embryos, particularly in cases involving high-efficiency sgRNA-guided editing. Therefore, in the revised text, we have modified the corresponding description In lines 80 to 82 as follows: “We discovered that MMEJ constitutes a substantial proportion of DNA repair during CRISPR-mediated embryonic gene editing, and in some loci, its efficiency even surpasses that of NHEJ repair.”. In the revised manuscript, we have correspondingly updated other pertinent descriptions, specifically from lines 108 to 133, from lines 117 to 131, and from lines 377 to 381.

**Revised Figure 5:** The relationship between sgRNA editing efficiency and the NHEJ/MMEJ ratio. (A) The MMEJ repair patterns are defined by a microhomology sequence of 1~25 bp. (B) The MMEJ repair patterns are defined by a microhomology sequence of 2~25 bp. Each circle corresponds to a specific sgRNA, with *Calcr*-sg2 highlighted. Data are presented as mean ± standard error of the mean (s.e.m.).

2. This reviewer speculates that embryo injection is associated with delivery of hyper quantities of CRISPR components, which leads to further recognition and cleavage of target DNAs with small indels. At the same time, delivery of hyper quantities of donor DNA may need to random integration in the genome. As such, it is advisable to investigate this possibility in offspring.

Response: We express our gratitude to the reviewer for their thoughtful suggestion. The matter of random insertions of exogenously supplied DNA templates is, in fact, the principal concern under investigation in our study. Our approach involves the examination of random integration via the monitoring of T-DNA analysis in plants. (*Ref: Distinct Patterns of HBV Integration and TERT Alterations between in Tumor and Non-Tumor Tissue in Patients with Hepatocellular Carcinoma*). We identify random insertion events when we detect any sequences belong to exogenous donor, including left/right-homology and mCherry, are inserted in none-targeted locus (Revised Figure 6A). We carefully analyzed WGS data derived from our GOTI experiment. After mapping NGS reads to exogenous donor and reference genome, we found out the discordant reads, which are defined as one reads of a pair successfully mapped to part of the exogenous donor and a same pair mapped to the reference genome (Revised Figure 6B). For the accuracy of the results, we set strict cut-off value that discordant reads are mapped more than 10 times. As the results shown in Revised Figure 6B, we found that the number of integration events in CATI samples is similar to that in control samples. These data indicate that, as compared to canonical knock-in method, CATI method would not introduce more random integration in our tested samples. In the revised version, we have incorporated additional descriptive context between line 424 and line 428, as well as from line 352 to line 360. Additionally, to augment the visual representation of our findings, we have included Figure S4L and Figure S4M.

**Revised Figure 6**. Analysis of donor DNA genome random insertion. (A) Illustration of random integration analysis. Any fragments of donor ligated to reference genome can be recognized as integration counts. After excluding counts mapped to KI site, the remaining counts represent random integration. (B) Location distribution of random integration across mouse genome. Red bars represent control. Green bars represent CATI. Two different results derived from different cutoff have been shown together. The numbers of integration event have been shown in the center of circus.

3. CRISPR‒Cas9-mediated DSBs in human embryos may lead to chromosome loss (Ref: Allele-Specific Chromosome Removal after Cas9 Cleavage in Human Embryos), which limits embryo gene editing safety for clinical use. The authors also need to provide data to address this concern. In addition, delivery of hyper quantities of CRISPR components can also lead to off-target cleavage and large deletions. The levels of large deletions after double-strand breaks in different cell types appear to be different. It is curious to know the levels of large deletions in embryo editing.

Response: We express our gratitude to the reviewer for their valuable suggestions, which are of great significance to this field. In order to elucidate the genetic changes underlying the CATI treatment, we amplified a ~5 kb region surrounding the stop codon of *Cdx2* and *Gata6* loci from pools of five embryos that were edited using Tild and CATI methods, respectively. Subsequently, we sequenced the PCR products using the PacBio platform and observed large deletions of read coverage around the cut site (stop codon) at both loci. Notably, we found a lower lost coverage ratio in the CATI group at both loci (*Cdx2*: 33.25% and 2.89% vs. 0.96% and 12.07%; *Gata6*: 16.14% and 6.94% vs. 0.54% and 1.10%) (see in Revised Figure 7). These results suggest that the CATI method does not induce more severe large deletions than Tild at our tested loci. Please refer to Revised Figure 6 for further details. In the revised version, we have incorporated additional descriptive context between line 361 and line 370, as well as from line 424 to line 427. Additionally, to augment the visual representation of our findings, we have included Figure S4N.

**Revised Figure 7.** The large deletion analysis of different gene Knock-in strategy on *Cdx2* and *Gata6* loci. Coverage of PacBio reads at the loci. The loci were PCR-amplified from a pool of embryos (5 embryos per sample), and the resulting products were sequenced using the PacBio platform. The red triangles indicate cut site. WT: unedited embryos, Tild: embryo edited by Tild method, CATI: embryos edited by CATI method. Gene location were shown below. Black rectangles indicate exons.

**Minors:**

1. MMEJ was designated when microhomologies were observed surrounding the break site. The length of microhomologies is usually more than 2 bp (Ref: Decoding non-random mutational signatures at Cas9 targeted sites). In the Introduction session, the authors mentioned MMEJ and cited a reference (Ref: MMEJ repair of double-strand breaks (director's cut): deleted sequences and alternative endings). In this reference, the length of microhomologies was 5-25 bp. This reviewer encourages the authors to examine multiple relevant papers and carefully define MMEJ.

Response: We express our gratitude to the reviewer for their meticulous review and valuable suggestions. Indeed, multiple methods currently exist to define MMEJ repair, and a definitive standard has yet to be established. We have integrated the relevant MMEJ classification definitions from some of existing literature and restructured the updated manuscript (*Ref: Decoding non-random mutational signatures at Cas9 targeted sites; Ref: Dynamics and competition of CRISPR–Cas9 ribonucleoproteins and AAV donor-mediated NHEJ, MMEJ and HDR editing; Ref: MMEJ repair of double-strand breaks (director's cut): deleted sequences and alternative endings; Ref: Synthesis-dependent microhomology-mediated end joining accounts for multiple types of repair junctions; Ref: Microhomology-mediated end joining: a back-up survival mechanism or dedicated pathway?*). In accordance with prior research, we classified the DNA repair pattern as NHEJ when insertion occurred, or in cases where microhomology sequence were not detected in vicinity of the deletion segment, or when the deletion involved only a single base. Conversely, deletion events accompanied by microhomology sequences (2~25 nt) were classified as MMEJ repair pattern. (see in Revised Figure 8A and 8B). In the revised manuscript, we have included additional context between line 108 and line 113 and replaced Figures 1C, 1D, and 1E.

**Revised Figure 8. A method of classification for distinct patterns of DNA damage repair outcomes is demonstrated using *Calcr* as a paradigmatic example.** (A) CRISPR-Cas9 cleavage and its subsequent repair processes are depicted, where the PAM sequence is denoted by red bases, the insertion sequence by purple bases, the deletion sequence by a yellow dotted line, and the micro-homologous arm by a red underline. (B) The repair profile of *Calcr*-sgRNA2 is presented, wherein the PAM sequence is highlighted in red, the missing sequence is represented by a gray base region, and the micro-homologous sequence is underlined in red.

2. There are multiple citation format inconsistencies: We believe the indel reduction will benefit to genome stability and integrity (Hsu et al., 2014; Wen et al., 2021). embryo injection and culturing, has been mentioned in previous publication (Liu et al., 2018). reliability of which has been confirmed by several studies (Wilde et al., 2021).

Response: We thank the reviewer for careful reading and kind suggestions. We have unified the format of all references in the revised manuscript.

3. Dozens of grammar errors or typos have been spotted.

Response: We would like to express our gratitude to the reviewer for their meticulous review of our manuscript, and we deeply regret any inadequacies in our writing. In response to the reviewer’s constructive feedback, we have enlisted the assistance of a native English speaker who is a friend of ours to refine the language in our manuscript. The revised version has undergone extensive editing and we hope that it now meets the necessary standards for acceptance.

**Reviewer #3:** Chen et al demonstrate three major things in their study. (1) They show that MMEJ is the predominant DNA repair process, but not NHEJ, in mouse zygotes. (2) They identify that PolQ is important for this process and (3) they test their idea of increasing the donor DNA insertion efficiency by down-regulating PolQ (using RNA editor CasRX) at a few loci.

**Responses**: We would like to express our gratitude to the reviewer for their thorough review of our manuscript. We appreciate the valuable comments and insightful questions raised by the reviewer, which have greatly contributed to the enhancement of our manuscript.

**Below are review comments and suggestions.**

1. One major question that is perplexing is that most of their donors contain several hundreds of bases of homology arms. If MMEJ relies on micro homologies of only a few bases of complementarity, the question is how can authors attribute MMEJ helps enhance the HDR (of donors containing hundreds of bases of homology arms).

Response: We thank the reviewer for the question. We think there are some misunderstandings, and we would like to apologize for the confusion caused by our imprecise description in the manuscript. In our study, we achieved a HDR efficiency improvement by inhibiting the MMEJ via knocking down a MMEJ key regulator *polq*. So, we attribute our improvement to MMEJ inhibition, instead of prompting MMEJ.

2. The authors used linear double stranded DNA donors for HDR experiments containing ~800 base homology arms. What is the rational or reason for choosing longer homology arms? Considering that MMEJ requires only short homologies, have authors tested shorter homology arms? Do longer or shorter homology arms matter? Related to this, long ssDNAs (with short homology arms) have been popularly used as donors for creating conditional knockout and Cre- or reporter- knock-in mouse models. Have authors tested how lssDNA donors perform in their CATI method. In other words, can MMEJ inhibition further enhance knock-in efficiency of lssDNA donors?

Response: We thank the reviewer for the professional and insightful questions.

(1) “Considering that MMEJ requires only short homologies, have authors tested shorter homology arms?”

Response: We would like to apologize for the confusion caused by our imprecise description in the manuscript. The aim of this study was to promote HDR by inhibiting the MMEJ repair pathway. We used long homology arm sequences that are more suitable for HDR repair, rather than short homology arm sequences for MMEJ repair. Thus, we did not test shorter homology arms in our manuscript.

(2) “The authors used linear double stranded DNA donors for HDR experiments containing ~800 base homology arms. What is the rational or reason for choosing longer homology arms? Do longer or shorter homology arms matter?”

Response: Thanks to the reviewer for noticing this key detail. Our linear double stranded DNA donor strategy mainly refer to previous study “Tild-CRISPR Allows for Efficient and Precise Gene Knock-in in Mouse and Human Cells”. They describe Tild-CRISPR (targeted integration with linearized dsDNA-CRISPR)-based strategy, a targeting strategy use donor DNA by PCR-amplified with 800bp homology arms (HA), which efficiently achieved DNA-targeted integration in mouse embryos. They have tried different length of HA, and found that 800bp was suitable choice (see in Revised Figure 9). In our study, we used the Tild-CRISPR as our control baseline, and found that the Tild-CRISPR [strategy](javascript:;) works well. So, we choose DNA donors for HDR experiments containing ~800 base homology arms.

**Data from (Yao et al. Developmental Cell 45, 526–536)**

**Revised Figure 9. Tild-CRISPR-Mediated Targeted Integration in Mouse Embryos.** (A) Schematic overview of different gene-targeting donors using CRISPR/Cas9 at the Actb locus. Green parallel lines, left/right homology arm; gray parallel lines, junk sequence; triangles, sgRNA target sites; HR, a homologous recombination donor; HMEJ, a homology-mediated end-joining donor; ssDNA, a singlestranded DNA donor; Tild, a linearized donor with 800-bp HAs; JS200 to JS2000, a linearized donor with 200- to 2,000-bp junk sequences adjacent to HAs. (B) Experimental design. Cas9 mRNA, sgRNA, and different donor vectors were injected into mouse zygotes, and the injected zygotes were cultured into blastocyst stage for fluorescence observation and genotyping analysis. (C) Knockin efficiencies indicated by percentage of mCherry+ blastocysts. Number above each bar denotes total blastocysts counted. *p < 0.05, ***p < 0.001, c2 test.

(3) “Related to this, long ssDNAs (with short homology arms) have been popularly used as donors for creating conditional knockout and Cre- or reporter- knock-in mouse models. Have authors tested how lssDNA donors perform in their CATI method. In other words, can MMEJ inhibition further enhance knock-in efficiency of lssDNA donors?”

Response: We appreciate the reviewer’s thoughtful question. Utilizing long single-stranded DNA (lssDNA) donors has proven to be an effective strategy for improving gene knock-in efficiency in various studies. We believe that inhibiting MMEJ could further enhance lssDNA-mediated gene knock-in efficiency. However, generating lssDNA donors can involve multiple methods that may introduce sequence errors, yield poor results, and contain double-stranded DNA (dsDNA) contamination. Acquiring a pure lssDNA template is inherently difficult and expensive. This is the reason why we did not pursue this approach. We have incorporated additional descriptions in the revised manuscript, specifically from lines 61 to 64.

3. The authors conclude that off target effects are not increased when PolQ is suppressed. However, the analysis is performed for only one locus/guideRNA. If authors claim this as one of the conclusions of their study, they should do this analysis for all guideRNAs used in the study to know if it is really the case. In general, off target analysis is not a major concern in case of mouse model generation because it is well established that they do not occur at frequencies that one needs to spend time and resources in finding them even if off target effects are present in founder mice they can be segregated by breeding. On the other hand, what is more important, specifically for this work, is to find out the rate of off target donor insertions (i.e., random insertions of exogenously supplied DNA templates). Testing off target insertions (not just off target Cas9 effects) would be important particularly because DNA repair pathway is perturbed (by suppressing PolQ).

Response: Thanks for the suggestions. We agree with the reviewer’s opinion that off-target is not the major concern in our study for model generation. Hence, we have changed descriptions on the off-target section. In revised manuscript, we just apply GOTI technique to preliminarily investigate the off-target editing while Polq is down-regulated. Our results show no more significant off-target editing in tested samples instead of original conclusion that none off-targets were observed. Next, random insertions of exogenously supplied DNA templates is actually the more attractive concern in this study. We analyze random integration by following T-DNA analysis in plants (*Ref: Distinct Patterns of HBV Integration and TERT Alterations between in Tumor and Non-Tumor Tissue in Patients with Hepatocellular Carcinoma*). We identify random insertion events when we detect any sequences belong to exogenous donor, including left/right-homology and mCherry, are inserted in none-targeted locus (Revised Figure 10A). We carefully analyzed WGS data derived from our GOTI experiment. After mapping NGS reads to exogenous donor and reference genome, we found out the discordant reads, which are defined as one reads of a pair successfully mapped to part of the exogenous donor and a same pair mapped to the reference genome (Revised Figure 10B). For the accuracy of the results, we set strict cutoff value that discordant reads are mapped more than 10 times. As the results shown in Figure B, we found that the number of integration events in CATI samples is similar to that in control samples. These data indicate that, as compared to canonical knock-in method, CATI method would not introduce more random integration in our tested samples. We have incorporated additional descriptions in the revised manuscript, specifically from lines 361 to 370, and added Figure S4N.

**Revised Figure 10**. Analysis of donor DNA genome random insertion. (A) Illustration of random integration analysis. Any fragments of donor ligated to reference genome can be recognized as integration counts. After excluding counts mapped to KI site, the remaining counts represent random integration. (B) Location distribution of random integration across mouse genome. Red bars represent control. Green bars represent CATI. Two different results derived from different cutoff have been shown together. The numbers of integration event have been shown in the center of circus.

3. This reviewer suggests that full sequences of all donor DNAs (dsDNAs and ssODNs) along with their annotations (demarcating homology arms, location of guide sequences with respect to new sequences added) and genotyping PCR primers should be provided as schematics. Such information can be best presented as schematics with color coded sequences for different regions.

Response: We express our gratitude to the reviewer for their careful reading and for identifying this oversight. We apologize for our negligence in providing such crucial detail information. We have now included Table S3 and Table S4 in the “Summary of the Supplementary Information” section. Table S3 contains all relevant primers/oligos used in this study, while Table S4 provides all donor DNA sequence information utilized. In accordance with the reviewer's suggestion, we have highlighted characteristic sequences with distinct colors and fonts (such as homology arms, sgRNA target sequences, PAM sequences, etc.).

3. Instead of 'marked elevation' in this sentence in the abstract (By down-regulating the expression of a MMEJ critical factor Polymerase Q (Polq) via RNA editor CasRX, we achieved a marked elevation in the targeted integration efficiency of linearized DNA fragments and single-strand oligonucleotides (ssODN) in mouse embryos and offspring, without increasing offtarget effects), the authors should indicate the increase in quantitative terms (for example 'fold increase'). Also, if the phrases 'without increasing off target effects' is retained, such analysis should be done for all loci/guides including off target insertions.

Response: We would like to express our sincere gratitude to the reviewer for bringing this matter to our attention. We have changed the description “we achieved a marked elevation in the targeted integration efficiency of linearized DNA fragments and single-strand oligonucleotides (ssODN) in mouse embryos and offspring, without increasing off-target effects.” with “By down-regulating the expression of a key MMEJ factor, Polymerase Q (*Polq*), using the RNA editor CasRX, we significantly improved the targeted integration efficiency of linearized DNA fragments and single-strand oligonucleotides (ssODN) in mouse embryos and offspring” in revised manuscript, see in line 27 to line 31.

3. The authors note that "We speculated that the latter was caused by the presence of large amounts of linearized donor DNA, which may be recognized by embryos as DNA damage." In regard to the experiment S1B and S1C, an important control would be NOT to create double stranded break in the genome. This can be done by excluding Cas9 and only inject guide and the donor DNA (?).

Response: We thank the reviewer for the thoughtful suggestion and concur with the notion that incorporating more appropriate controls will enable the exclusion of potential influences from the gene-editing system. In accordance with the reviewer's recommendation, we have designed and executed a new series of RNA-seq and RT-qPCR experiments to assess the expression levels of DSB repair-related genes. For each experiment, we established seven distinct conditions as follows: (1) blank; (2) dilution solvent; (3) Cas9 only; (4) Cas9+scramble sgRNA; (5) Cas9+sgRNA; (6) donor only; (7) Cas9+sgRNA+donor. The injection procedure and sample preparation were consistent with those described in the original manuscript.

In the RNA-seq results, we observed that the expression levels of most canonical DNA damage repair-related genes did not exhibit significant alterations, which aligns with previous findings (see in revised Figure 11A and 11B). The “blank” group and “dilution” group displayed similar *polq* levels, approximately half that of the “Cas9+sgRNA+donor” group, which is consistent with the data presented in the original manuscript. However, we noted an upregulation of *polq* levels in the “Cas9+sgRNA” group in this experimental iteration, a finding not observed in the original manuscript's data. We hypothesize that the disparity between the two datasets may stem from the low expression of *polq* in embryos (see in revised Figure 11C and 11D). A re-analysis of a published RNA-seq database (GSE66582) similarly revealed low *polq* expression levels (FPKM < 1) (see in revised Figure 11E).

In the RT-qPCR experiment, which offers higher sensitivity for detecting low expression genes, we observed an upregulated polq level not only in group “(7) Cas9+sgRNA+donor” but also in group “(6) donor only” (see in revised Figure 11F). The elevated *polq* level in the “Cas9+sgRNA+donor” group aligns with our previous data from the original manuscript. The increased polq level in the “donor only” group further corroborates our speculation in the main text, stating, “We hypothesized that the latter was induced by the presence of substantial quantities of linearized donor DNA, which may be recognized by embryos as DNA damage.” Overall, our results indicate that the editing system, encompassing Cas9, sgRNA, and dilution buffer, imparts minimal influence on the expression of the majority of DNA damage repair genes. We have now integrated the updated data into the revised manuscript as Fig. 1F and have modified the corresponding text from line 153 to line 157 to reflect these changes.

**Revised Figure 11.** DNA repair-related gene expression. (A) Principal component analysis of transcriptomes from seven group samples. Each dot represents one sample, and each sample contains five embryos. (B) The expression of analyzed DNA repair-related genes from seven groups. DNA repair pathway include HR, NHEJ, SSA and MMEJ. (C) and (D) The relative FPKM value of *polq* from seven groups. (E) The relative FPKM value from data GSE66582. (F) The relative expression level of indicated genes (*Rad52*, *Ku70* and *polq*). *p < 0.05, **p < 0.01.

**Minor comments:**

3. Introduction and discussion sections mention a few DNA repair pathway papers. Those papers are predominantly related to cell culture experiments (and the discussion of animal model generation methods/papers is somewhat low in these sections). Considering that all experiments (of testing of the new method of modulating MMEJ pathway) pertain to generation of animal models, the introduction and discussion sections can be expanded a bit more to include some of the commonly used methods of creating knock-in mouse models and discuss the challenges of those methods. The authors indeed mention this challenge in the cover letter, which can be added to the manuscript and expanded a bit more.

Response: We express our gratitude to the reviewer for their constructive recommendations. In accordance with these suggestions, we have expanded the introduction and discussion sections within the revised manuscript to provide a more comprehensive context.

Revised text 1 added in the introduction section: “Despite the advances in genome editing, the efficiency of precise DNA fragment integration through HDR remains suboptimal. Recently, several methods have been reported to enhance gene knock-in efficiency in zygotes via Cas9 (mRNA or ribonucleoprotein) injection. These approaches include the utilization of transgene templates cleavable into 800 bp homology arms in vivo (HMEJ strategy) [6], linearized templates derived from PCR amplification (Tild-CRISPR) [7], long single-stranded DNA (ssDNA) templates (Easi-CRISPR) [8], short ssODN templates [9], templates that activate the MMEJ or NHEJ pathways [10-12], and templates modified with Biotin-avidin system (CAB system) [13, 14]. Furthermore, chemically modified single guide RNAs (sgRNA) [15] and commercially available, chemically modified tracrRNAs and crRNAs [16, 17] have also been employed to augment gene knock-in efficiency. However, only a limited number of successfully targeted loci have been documented thus far [8, 12, 18]. Certain methods, such as Easi-CRISPR and ssODN, are constrained by the size limitations of DNA fragments for insertion (typically <1 kb). Other approaches, including those based on MMEJ or NHEJ templates, are restricted by the generation of imprecise junctions at editing sites. Additionally, methods involving modified templates or sgRNAs may be hindered by cytotoxicity. Furthermore, techniques employing long ssDNA-based Easi-CRISPR are constrained by challenges in obtaining templates and elevated costs.” Line 46 to Line 64 of the revised manuscript.

Revised text 2 added in the discussion section: “Limitation of the study: We would like to emphasize that current methodologies, which rely on edited sequences for differentiating repair patterns, may not be sufficiently rigorous, as they are unable to distinguish uncut DNA from a flawless DNA lesion repaired via HR, NHEJ or MMEJ pathways. Nevertheless, our conclusions regarding MMEJ in embryos remain valid, as the ratio of NHEJ and MMEJ is unlikely to be significantly affected by a small number of uncut DNA events when using sgRNAs with high editing performances. However, more precise and user-friendly techniques for DNA repair identification are still desirable. Moreover, CATI offers an alternative, reproducible approach for gene integration in embryos. Yet, for some sites, gene integration frequencies have not seen substantial improvements, highlighting the complexity of the embryo DNA repair pathway. We anticipate the development of more advanced approaches for gene integration in the future.” Line 429 to line 441.

4. Figure 1C; If I understand correctly, the sequence used (to describe the concepts of three types of DNA repair) is hypothetical as I could not find a classical PAM sequence close to the repaired nucleotides (shown in red). Perhaps authors can use one of their actual examples (guide and the repair outcomes) to describe the idea?

Response: We would like to express our sincere gratitude to the reviewer for bringing this matter to our attention. We are so sorry for the negligence of such important detail information. We have changed the pattern diagram (see in Revised Figure 12) in the revised manuscript as Fig. 1C.

**Revised Figure 12.** Illustration showing the strategy for discriminating repair type through editing outcome. CRISPR-Cas9 cleavage and its subsequent repair processes are depicted, where the PAM sequence is denoted by red bases, the insertion sequence by purple bases, the deletion sequence by a yellow dotted line, and the micro-homologous arm by a red underline.

5. Figure S2D. Number of zygotes transferred to each surrogate mother is indicated (which is 20 for all mice) but number of zygotes injected need to be included.

Response: We express our sincere gratitude to the reviewer for their careful reading of our manuscript and for providing valuable suggestions. We deeply regret any oversight in the presentation of crucial details. In response to the reviewer's feedback, we have included Table S2 in the “Summary of the supplementary information” section. Table S2 provides a summary of the mice line generated by the CATI method. Furthermore, we have updated Figure S2D to include specific information on the number of zygotes injected. As a standard practice, we inject 28 embryos to ensure optimal embryonic development.

6. The authors have manually calculated NHEJ/MMEJ efficiencies using ICE data (described in pages 20-21 of merged pdf file). They present this data as charts in figure 1 and S1, and this data forms the basis for their main conclusion. Could the authors also provide details how they did the manual analysis and include the intermediary files of information as supplemental files?

Response: We wish to extend our sincere appreciation to the reviewer for drawing our attention to this issue. As per the reviewer's suggestion, we have incorporated the “Workflow diagram” (see in Revised Figure 13) in Figure S1A and added the intermediary files of Table S1 to the revised manuscript.

**Revised Figure 13**. Workflow diagram for sequence analysis using the ICE website. The number 1 represents the sgRNA sequence without the PAM region, number 2 represents the normal ab1 sequencing file of the target site prior to editing, number 3 represents the ab1 sequencing file after editing, number 4 represents the overall indel ratio at the target site, and number 5 represents the possible sequence types and their respective proportions at the target site after editing.

7. A few sentences containing typos/grammatical errors are copied below, as examples but the manuscript needs English editing throughout.

Response: We would like to express our sincere appreciation for your professional review of our article, and we deeply regret any errors or deficiencies in our writing and grammar. In response to your valuable suggestions, we have made the necessary corrections to our previous draft. In addition, we enlisted the assistance of a native English speaker to help edit the entire manuscript.

(1) We found that MMEJ was a predominate form in nuclease-mediated DSB repair (predominant)

We appreciate the reviewer’s suggestion, and in response to the reviewer’s feedback, we have reclassified MMEJ and NHEJ in the revised version. Furthermore, we have modified the inappropriate statement of “MMEJ repairing is the dominant role” to “MMEJ repair plays a crucial role in embryonic development, and in some loci, its contribution is even greater than that of NHEJ.” In the revised version, we have replaced “We found that MMEJ was a predominate form in nuclease-mediated DSB repair” with “We discovered that MMEJ constitutes a substantial proportion of DNA repair during CRISPR-mediated embryonic gene editing, and in some loci, even surpasses the efficiency of NHEJ repair.”

(2) Thus, the CATI method could be a prior option for developing gene-edited monkey models and human germ-cell gene therapies (did author mean 'preferred option', not 'prior option'?)

We are grateful for the reviewer's suggestion, and we apologize for any confusion caused by the incorrect wording. As advised, we have corrected the phrase “prior option” to “preferred option” in lines 88 to 89 of the revised manuscript.

(3) Although it is reasonable that transiently knockdown of Polq might induce limited harmful effect, we still performed Polq knockdown test to examine whether embryos develop normally (transient)

We express our gratitude to the reviewer for their helpful suggestion. As recommended, we have made the appropriate modification of “transiently” to “transient” in line 189 of the revised manuscript.

(4) As results shown, embryos with…. ('As shown in results' could be better?)

We would like to express our appreciation for the reviewer’s valuable suggestion. As a result of the comprehensive grammatical editing of the entire manuscript, we have revised the original sentence to read as follows: “The results demonstrated that embryos with transient *Polq* knockdown developed into blastocysts comparably to controls.” in lines 191 to 193 of the revised manuscript.

(5) Overall, all targets showed an increased gene integration efficiency in varying degree (Fig. 2B). (….varying degrees)

We express our gratitude to the reviewer for their suggestion. As recommended, we have made the appropriate modification of “varying degree” to “varying degrees” in lines 198 to 199 of the revised manuscript.

Furthermore, in addition to addressing the revisions suggested by the reviewer, we engaged a native English speaker. Thanks to our friend’s valuable input, the manuscript underwent extensive editing. We have taken care to highlight all instances of sentence modification in the revised text.

Overall, we have made concerted efforts to improve the manuscript without altering its core content and structure. While we have not provided a detailed list of the changes made, we have marked them in yellow in the revised paper. We express our sincere gratitude to the reviewers for their diligent work and hope that our revisions will be deemed satisfactory.

**Second round of review**

**Reviewer 1**

All of my comments have been addressed proper in the revised manuscript.

**Reviewer 2**

The authors have addressed all my concerns.

**Reviewer 3**

This reviewer is impressed with the revised version of the manuscript. The authors seem to have made exceptional effort in performing additional experiments, in presenting the data and in significantly improving the manuscript. This reviewer was confused with the previous version of the manuscript. The confusion was partly due to the presentation style, but the reviewer was also responsible for incorrect understanding of the central point of the paper, which was reflected in reviewer’s comments 1 and 2. The authors have provided explanation to clear reviewer’s misunderstanding of the paper’s findings. The reviewer would like to convey sincerely apologizes to authors for the error.

One minor suggestion to enhance the accessibility of the manuscript and to make it easily understandable (to general readers) is that if authors can provide a figure illustrating the central message of the paper, viz. inhibition of MMEJ pathway by knocking down PolQ (a key regulator of MMEJ) can enhance HDR. This can be a something like a graphical abstract or a concluding figure at the end of the manuscript, provided such a format is okay with editors or the journal.

Congratulations to authors for the great study
